# Pentafluoroethyl Sulfoximine Reagent for the Photocatalytic Pentafluoroethylation–Difunctionalization of Styrene Derivatives

**DOI:** 10.1021/acs.orglett.5c05212

**Published:** 2026-01-21

**Authors:** Lu Lin, Gabriel Goujon, Bruce Pégot, Guillaume Dagousset, Elsa Anselmi, Emmanuel Magnier, Gavin Chit Tsui

**Affiliations:** † Department of Chemistry, 26451 The Chinese University of Hong Kong, Shatin, New Territories, Hong Kong SAR 999077, China; ‡ Shanghai-Hong Kong Joint Laboratory in Chemical Synthesis, The Chinese University of Hong Kong, Shatin, New Territories, Hong Kong SAR 999077, China; § 511723 Université Paris-Saclay, UVSQ, CNRS, UMR 8180 Institut Lavoisier de Versailles, 78035 Cedex Versailles, France; ∥ Université de Tours, Faculté des Sciences et Techniques, 37200 Tours, France

## Abstract

We herein describe the synthesis of a pentafluoroethyl sulfoximine and its application in the pentafluoroethylation–difunctionalization of styrene derivatives. This sulfoximine reagent acts as a source of pentafluoroethyl radical to react with styrenes under mild photocatalytic conditions. By using simple nucleophiles, the pentafluoroethyl group can be introduced to styrenes with concomitant formation of C–O/C–N/C–S/C–C bonds, which significantly broadens the difunctionalization scope involving pentafluoroethylation compared to previous copper-based methods.

## Graphical abstract

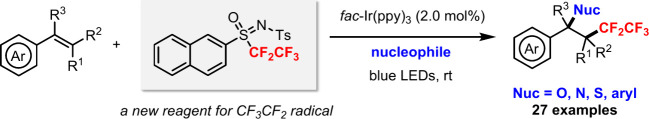

Perfluoroalkylation of alkenes is a direct and efficient way to introduce fluorine-containing groups into organic molecules due to the fact that alkenes are abundant and inexpensive feedstocks.^1^ Trifluoromethylation of alkenes with concomitant functionalization (i.e., difunctionalization) has been achieved with broad scope.^2^ By contrast, the difunctionalization of alkenes involving *pentafluoroethylation* is very limited. The pentafluoroethyl (−CF_2_CF_3_) group has been shown to be relevant for pharmaceutical applications.^3^ More recently, we have demonstrated that pentafluoroethylated compounds can serve as useful building blocks for emerging fluorinated motifs by C–F bond functionalization.^4^ Our group has a continuing interest in developing pentafluoroethylation reactions using alkenes. Previously, we have reported that unactivated alkenes and styrene derivatives can be pentafluoroethylated using a [CuCF_2_CF_3_] reagent derived from pentafluoroethane (Scheme 1).^5^ For instance, allylic pentafluoroethylated products were obtained from unactivated alkenes (Scheme 1a).^5a^ Bis-pentafluoroethylated cyclic compounds were obtained from 1,6-dienes (Scheme 1b).^5b^ Styrene derivatives afforded various pentafluoroethylated products, but only chloropentafluoroethylation was achieved satisfactorily for difunctionalization due to the presence of CuCl in the reaction mixture (Scheme 1c).^5c^

**Scheme 1:**
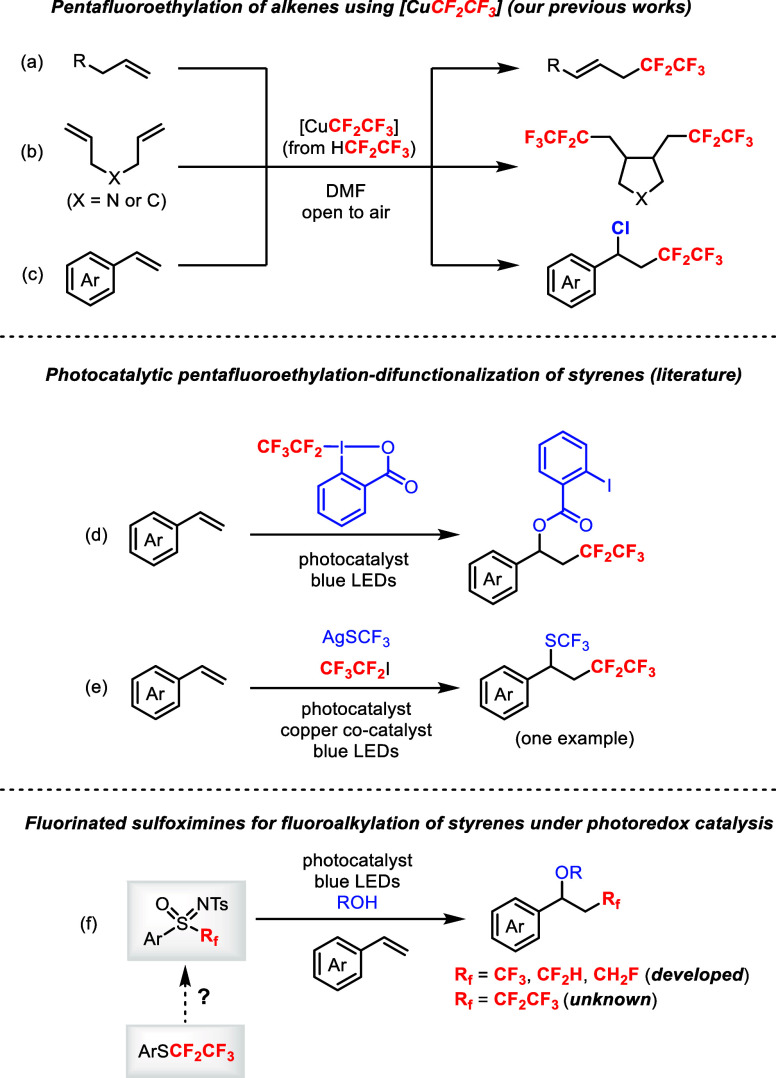
Pentafluoroethylation–Difunctionalization of Alkenes

In recent years, photoredox catalysis has emerged as a powerful tool for difunctionalization of styrenes involving perfluoroalkylation.^6^ We and others have reported a variety of photocatalytic trifluoromethylation,^7^ difluoromethylation,^8^ and monofluoroethylation^9^ reactions of styrene derivatives with concomitant construction of new C–C and C–heteroatom bonds. However, examples of photocatalytic pentafluoroethylation are extremely rare in the literature. Hong, An, and co-workers described an acyloxy pentafluoroethylation of styrenes using the Togni II reagent (Scheme 1d).^10a^ The C–O bond formation arises from the same fragment as the Togni reagent. The C–S bond formation was also possible but limited to the use of potassium *O*,*O*-diethyl phosphorothioate. Song and co-workers demonstrated one example of pentafluoroethylation of styrenes with trifluoromethylthiolation (Scheme 1e).^10b^ The use of AgSCF_3_ and copper cocatalyst was necessary. Thus, a general protocol for the pentafluoroethylation–difunctionalization of styrenes with broad scope is still lacking.

One viable solution to this problem is the development of new pentafluoroethylation reagents that are applicable in photoredox catalysis. Fluorinated *sulfoximines* are versatile reagents for introducing fluoroalkyl groups.^11^ We and others have shown that the fluorinated sulfoximines are useful sources of fluoroalkyl radicals under photoredox catalysis.^7–9^ In particular, the *N*-tosyl sulfoximines^12^ containing CF_3_, CF_2_H, and CH_2_F groups have been successfully prepared and employed in the photocatalytic oxyfluoroalkylation of styrenes with water or alcohols (Scheme 1f).^7h,8a,9^ However, to the best of our knowledge, the corresponding *pentafluoroethyl* sulfoximine remains unknown. We wish to report herein the efficient synthesis of this unprecedented pentafluoroethyl reagent on a large scale and its application in a wide range of new pentafluoroethylation–difunctionalizations of styrenes.

We first surmised that the targeted pentafluoroethyl sulfoximine could be prepared from the corresponding pentafluoroethyl sulfide. To test this hypothesis, we synthesized the pentafluoroethyl sulfide **1** from the corresponding thiosulfonate and TESCF_2_CF_3_ on a gram scale using our previous protocol (Scheme 2).^13^ The ultimate source of CF_2_CF_3_ is the inexpensive gas *pentafluoroethane* (HCF_2_CF_3_, HFC-125), which was used to generate TESCF_2_CF_3_.^14^ The choice of naphthyl substituent group was to avoid volatility of the compound (bp of PhSC_2_F_5_ = 53 °C, 28 mmHg). Next, one-pot synthesis of the *N*H-sulfoximine **2** from **1** was achieved using (diacetoxyiodo)­benzene (PIDA) and ammonium carbamate.^15^ Final tosylation of **2** afforded the *N*Ts-sulfoximine **3** on a gram-scale. The structure of **3** was unambiguously confirmed by X-ray crystallography.

**Scheme 2:**
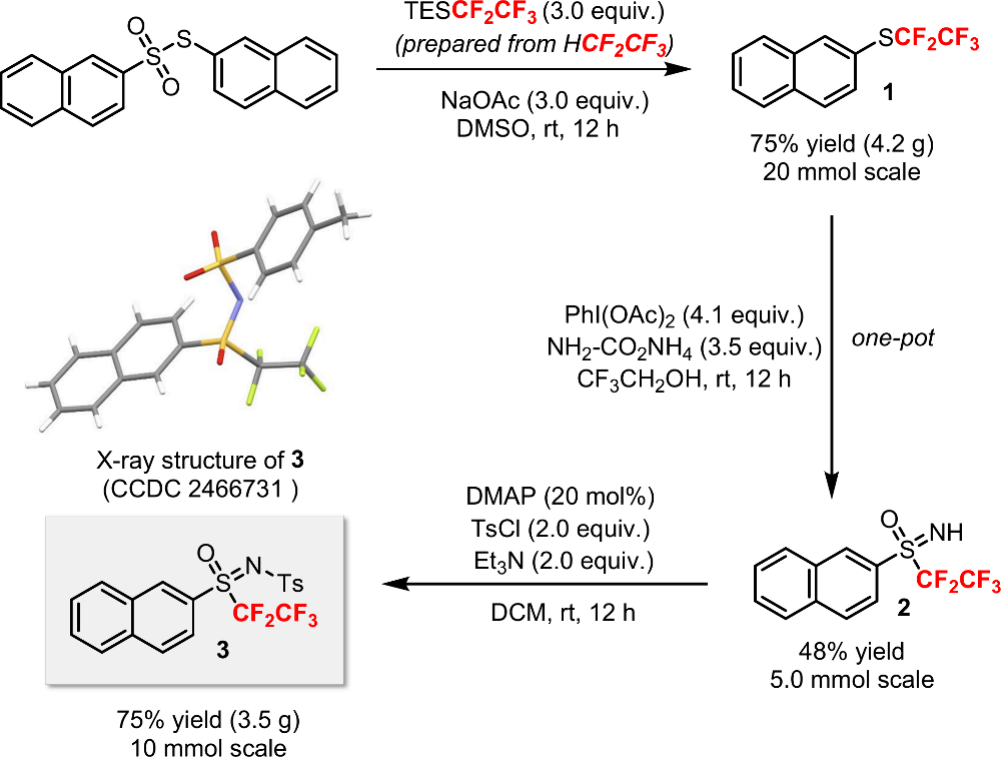
Preparation of a New Pentafluoroethyl *N*-Tosyl Sulfoximine

The capability of **3** to act as a source of a pentafluoroethyl radical under photoredox catalysis was uncertain at the outset. To this end, we tested **3** in the methoxypentafluoroethylation of styrene derivative **4a** using methanol under photocatalytic conditions (Table 1).^16^ Screening of various photocatalysts **PC-1** to **PC-5** under blue light irradiation revealed that *fac*-Ir­(ppy)_3_ (**PC-5**) was an efficient catalyst affording product **5a** in 71% yield (Table 1, entries 1–5). Further screening of solvents showed that MeCN could improve the yield to 90% (Table 1, entries 6–10). On the other hand, using MeOH as the solvent gave comparable yield (Table 1, entry 11). Using excess styrene **4a** led to a lower yield (Table 1, entry 12). Finally, control experiments showed that both irradiation and photocatalyst were required for the reaction (Table 1, entries 13–14).

**Table 1:**
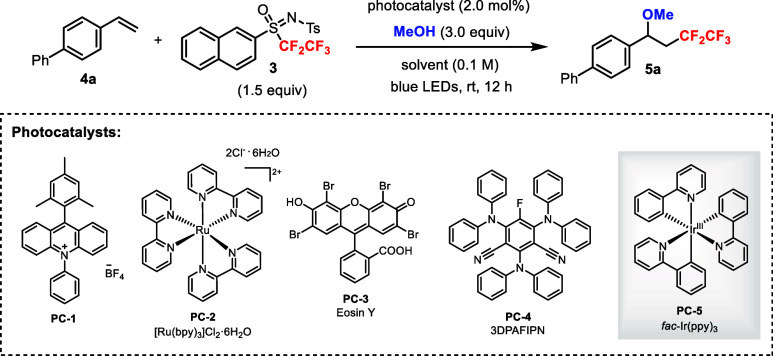
Optimization Studies for the Photocatalytic Methoxypentafluoroethylation of Styrene **4a** with Sulfoximine **3** ^a^

entry	photocatalyst	solvent	yield (%) of **5a** ^b^
1	**PC-1**	CH_2_Cl_2_	10
2	**PC-2**	CH_2_Cl_2_	<5
3	**PC-3**	CH_2_Cl_2_	<5
4	**PC-4**	CH_2_Cl_2_	<5
5	**PC-5**	CH_2_Cl_2_	71
6	**PC-5**	acetone	36
7	**PC-5**	DMF	<5
8	**PC-5**	THF	24
9	**PC-5**	EtOAc	10
**10**	**PC-5**	**MeCN**	**90**
**11** ^c^	**PC-5**	**MeOH**	**85**
12^c^ ^,^ ^d^	**PC-5**	MeOH	70
13^e^	**PC-5**	MeCN	<5
14	none	MeCN	<5

a Unless specified otherwise, reactions were carried out using **4a** (0.1 mmol) under argon.

b Determined by ^19^F NMR analysis of the crude mixture using benzotrifluoride as the internal standard.

c Used MeOH as solvent (0.1 M).

d Used 1.5 equiv of **4a**.

e In the dark.

The scope of the alkenes was subsequently investigated under the optimized photocatalytic conditions (cf. Table 1, entry 13) using MeOH as the solvent (Scheme 3). Various functional groups were tolerated in the styrene derivatives (**5a**–**g**), including electron-donating (**5d**), electron-withdrawing (**5e**), and halogen (**5f**–**g**) groups. Reaction was also performed on a 1.0 mmol scale in 80% yield (**5a**). 1,1-Disubstituted alkenes such as α-methylstyrene and 1,1-diphenylethylene smoothly afforded products **5h** and **5i**, respectively. In comparison, 1,2-disubstituted alkenes such as *trans-*β-methylstyrene and *trans*-stilbene gave products **5j** and **5k** in lower yields, respectively. Indene, a cyclic alkene, gave the desired product **5l** in a high yield. Only trace product was detected from an unactivated alkene.

**Scheme 3:**
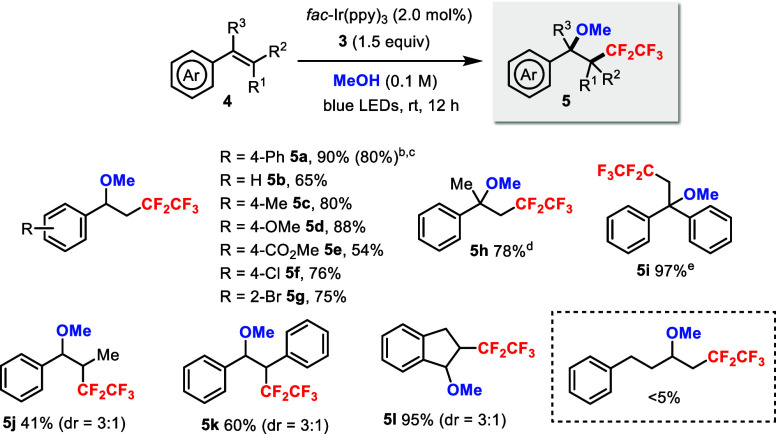
Photocatalytic Methoxypentafluoroethylation of Styrene Derivatives Using Sulfoximine **3** ^a^

a Unless specified otherwise, reactions were carried out using **4** (0.2 mmol) under argon, with isolated yields.
b 1.0 mmol scale.
c With naphthyl CF_3_ sulfoximine: 89% CF_3_ product; with phenyl CF_3_ sulfoximine: 93% CF_3_ product.
d With naphthyl CF_3_ sulfoximine: 76% CF_3_ product.
e With naphthyl CF_3_ sulfoximine: 64% CF_3_ product.

Besides methanol, other nucleophiles (Nuc-H) could also be employed using the optimized conditions (cf. Table 1, entry 10) to achieve the *pentafluoroethylation*–difunctionalization of the styrene derivative **4a** (Scheme 4). Common alcohols including ethanol (**6a**), isopropanol (**6b**), *tert*-butanol (**6c**), benzyl alcohol (**6d**), and cyclopentanol (**6e**) afforded the desired products in moderate yields. Phenol could also be used, albeit with a lower yield (**6f**). Even acetic acid promoted product formation (**6g**). Benzyl mercaptan (**6h**) and aniline (**6i**) were also suitable nucleophiles. By using a water/acetonitrile mixture, *hydroxypentafluoroethylation* was achieved in product **6j** in good yield. This result was quite remarkable since previously the hydroxypentafluoroethylation could only take place with α-methylstyrenes using excess [CuCF_2_CF_3_] reagent and B_2_pin_2_.^5c^ An electron-rich arene could also provide the products **6k**–**l** in high yields. Thus, the pentafluoroethylation of styrene derivatives with concomitant formation of C–O, C–S, C–N, and C–C bonds was successfully demonstrated.

**Scheme 4:**
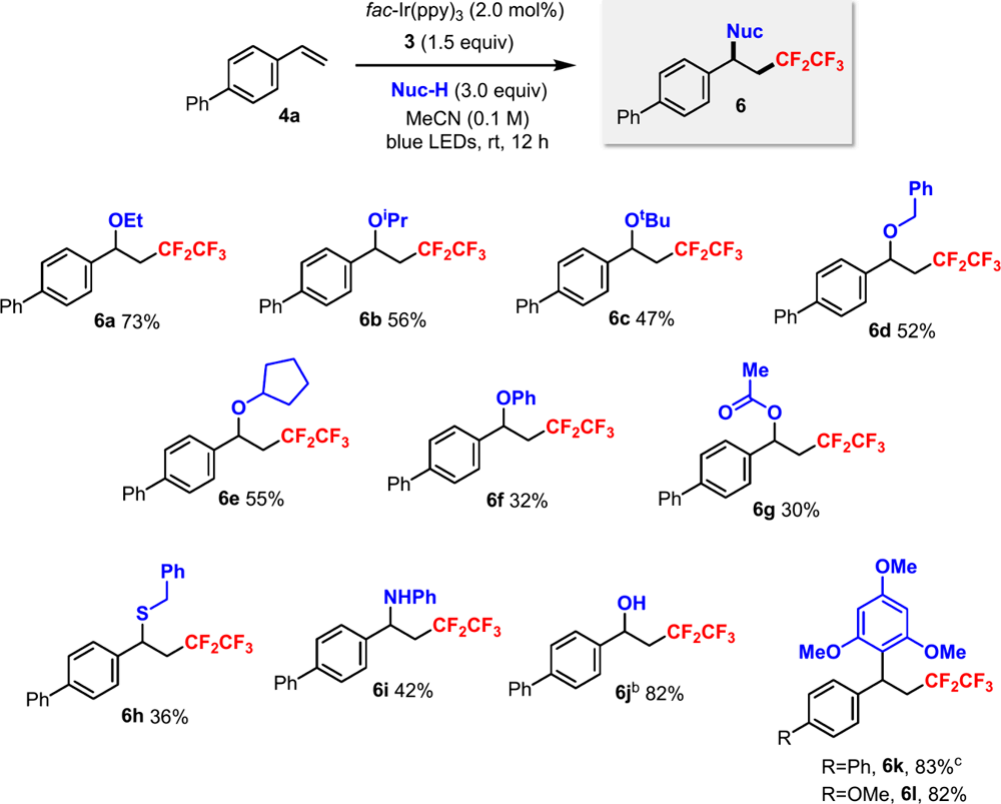
Photocatalytic Pentafluoroethylation–Difunctionalization of Styrene Derivatives Using Sulfoximine **3** ^a^

a Unless specified otherwise, reactions were carried out using **4a** (0.2 mmol) under argon, with isolated yields.
b H_2_O/MeCN = 1:9 (0.1 M).
c With naphthyl CF_3_ sulfoximine: 65% CF_3_ product; with phenyl CF_3_ sulfoximine: 73% CF_3_ product.

Furthermore, the challenging C–H pentafluoroethylation of arenes was achieved with sulfoximine **3** photocatalytically (Scheme 5). In our previous work, this was only possible when using excess amounts of the [Ph_4_P]^+^[Cu­(CF_2_CF_3_)_2_]^−^ reagent.^14a^

**Scheme 5:**
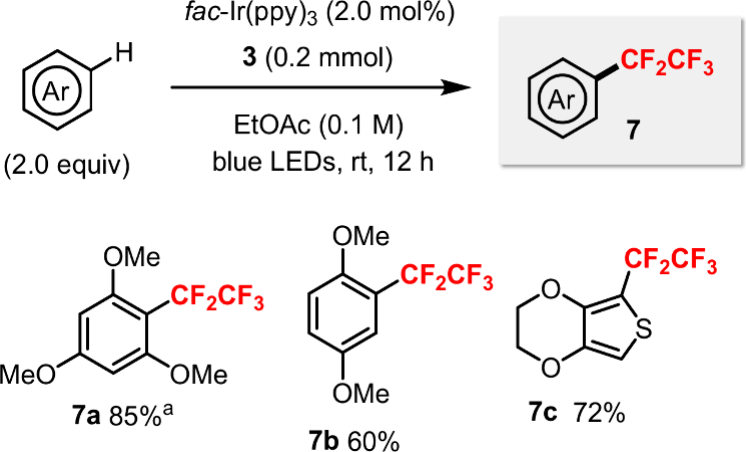
Photocatalytic Pentafluoroethylation of C–H Bonds Using Sulfoximine **3**

a With naphthyl CF_3_ sulfoximine: 77% CF_3_ product; with phenyl CF_3_ sulfoximine: 83% CF_3_ product.

We have also compared the reactivities between reagent **3** and *trifluoromethylated N*Ts-sulfoximine analogues for the trifluoromethylation–difunctionalization of styrene derivatives (cf. Schemes 3 and 4 footnotes) and C–H bond trifluoromethylation (cf. Scheme 5 footnotes). Under identical conditions, naphthyl and phenyl CF_3_ *N*Ts-sulfoximine reagents have shown similar yields for the trifluoromethylation products as compared to **3**, proving that the nature of the aryl and perfluoroalkyl groups of our sulfoximines has little impact on the reactivity (see SI for details).

Control experiments were carried out to gain more insight into the reaction pathway (Scheme 6). The radical clock experiment using cyclopropane **8** led to the ring-opened product **9** (Scheme 6a). Adding a radical scavenger TEMPO to the standard conditions completely inhibited the product formation (Scheme 6b), and the TEMPO–CF_2_CF_5_ species was detected by HRMS. These results strongly suggested a radical mechanism for the photocatalytic pentafluoroethylation–difunctionalization of styrenes.

**Scheme 6:**
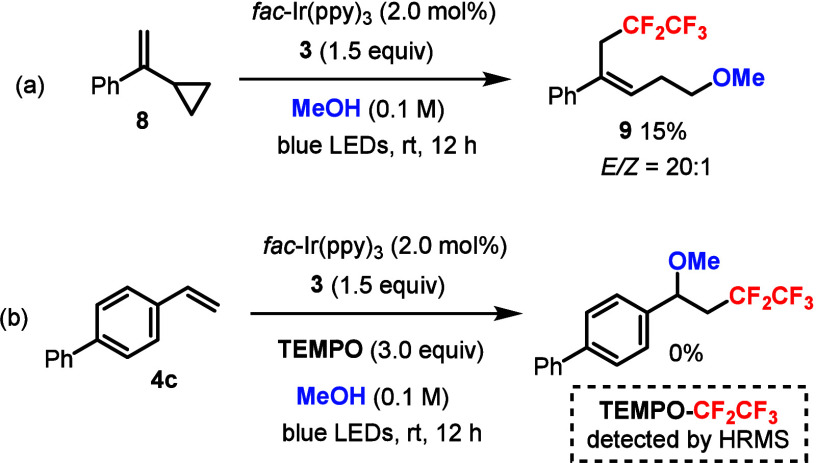
Control Experiments

In addition, the redox potential of reagent **3** was measured by cyclic voltammetry (see Supporting Information). Its reduction potential value was measured at *E*_1_ = −1.61 V relative to the SCE (saturated calomel electrode). It is worth noting that the *S*-naphthyl *S*-CF_3_ analogue was measured at a similar potential of −1.58 V vs SCE, while the *S*-phenyl *S*-CF_3_ sulfoximine has a lower potential of −1.73 V vs SCE. These results highlight the fact that the naphthyl substituent of the sulfoximine slightly improves the ease of reduction of the reagent. Luminescence quenching experiments were also carried out. The quenching rates determined using Stern–Volmer plots showed no quenching of the excited state Ir* of the iridium complex by the styrene **4a**, whereas the sulfoximine **3** efficiently quenched the excited complex Ir* (see details in the SI). Based on the above experiments and literature evidence,^7e,8a^ the following plausible photoredox catalytic cycle is proposed for the pentafluoroethylation–difunctionalization of styrenes **4** with the sulfoximine reagent **3** (Scheme 7). The photocatalyst *fac*-Ir­(ppy)_3_ (**Ir** ^**III**^) is first excited by visible light (blue LEDs) to become excited *Ir ^**III**^, a strong one-electron reductant that *reduces* sulfoximine **3** via a single-electron transfer (SET) process to generate **A** and **Ir** ^**IV**^. Fragmentation of **A** provides the key pentafluoroethyl radical and the sulfinamide **B** (detectable by HRMS).^16^ The CF_3_CF_2_ radical reacts with styrene derivative **4** regioselectively to provide the benzylic radical **C** and form the carbon–CF_2_CF_3_ bond. The strongly oxidizing **Ir** ^**IV**^ *oxidizes* **C** via SET to form the benzylic carbocation **D** and regenerates **Ir** ^**III**^. Finally, trapping **D** with a nucleophile (Nuc-H) leads to difunctionalized products **5/6**.

**Scheme 7:**
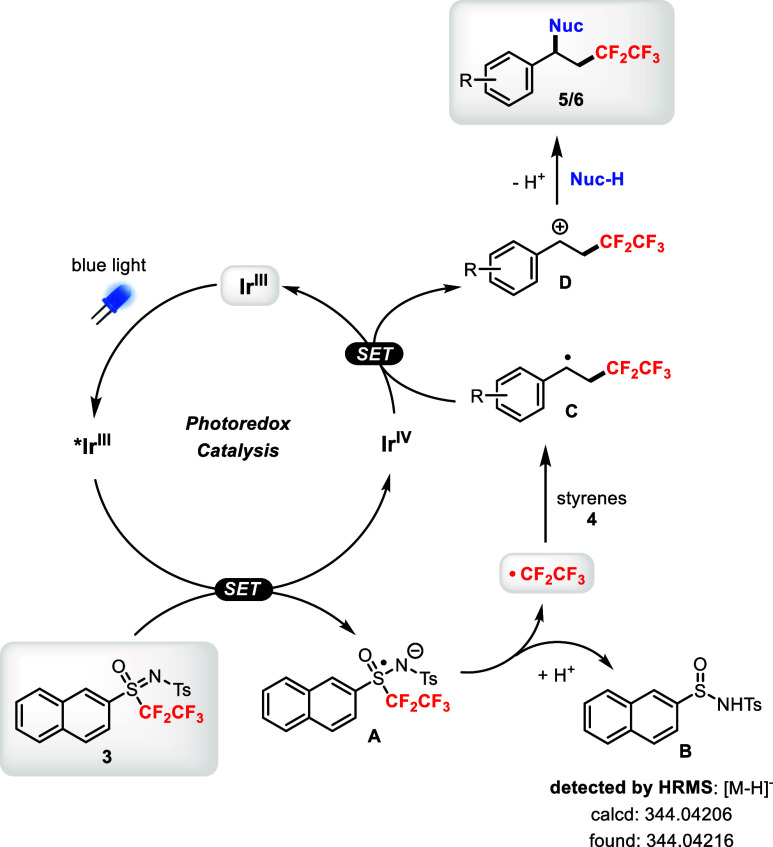
Plausible Mechanism for the Photocatalytic Pentafluoroethylation–Difunctionalization of Styrenes **4** Using the Sulfoximine Reagent **3**

In summary, we have synthesized a new pentafluoroethyl *N*Ts-sulfoximine as a source of a pentafluoroethyl radical. Under mild photocatalytic conditions, the three-component reaction involving the sulfoximine reagent, styrene derivatives, and nucleophiles could take place regioselectively to introduce the pentafluoroethyl group to styrenes with concomitant construction of carbon–heteroatom and carbon–carbon bonds. Further development of radical pentafluoroethylation using this reagent is ongoing in our laboratories.

## Supplementary Material



## Data Availability

The data underlying this study are available in the published article and its Supporting Information.

## Notes

Full experimental details, characterization data, NMR spectra, and single-crystal X-ray data (PDF)
