# Supplementary material for: Pentafluoroethyl Sulfoximine Reagent for the Photocatalytic Pentafluoroethylation–Difunctionalization of Styrene Derivatives
Source: Org Lett. 2026 Jan 21;28(5):1715–9. doi: 10.1021/acs.orglett.5c05212 (PMC12888017; doi:10.1021/acs.orglett.5c05212)
Supplement: Supplementary file 1 [file ol5c05212_si_001.pdf]

Supporting Information

A Pentafluoroethyl Sulfoximine Reagent for the Photocatalytic Pentafluoroethylation-Difunctionalization of Styrene Derivatives

Lu Lin,[a] Gabriel Goujon,[c] Bruce Pégot,[c] Guillaume Dagousset,[c] Elsa Anselmi,[c,d] Emmanuel Magnier[c*] and Gavin Chit Tsui[a,b]*

[a] Department of Chemistry, The Chinese University of Hong Kong, Shatin, New Territories, Hong Kong SAR, China

[b] Shanghai-Hong Kong Joint Laboratory in Chemical Synthesis, The Chinese University of Hong Kong, Shatin, New Territories, Hong Kong SAR, China

[c] Université Paris-Saclay, UVSQ, CNRS, UMR 8180 Institut Lavoisier de Versailles, 78035 Versailles Cedex, France

[d] Université de Tours, Faculté des Sciences et Techniques, 37200 Tours, France

* Email: gctsui@cuhk.edu.hk; Emmanuel.magnier@uvsq.fr.

Experimental Procedures and Spectral Data

Table of Contents:

I. General Experimental S3
II. Experimental Procedures S4
III. Control Experiments S10
IV. Optimization Studies S11
V. Single Crystal X-Ray Structure Determination of Compound 3 S14
VI. Comparison studies S16
VII. Stern-Volmer experiments S17
VIII. Reduction potential measurement S18
IX. Characterization Data of Products S19
X. Reference S30
XI. Spectra S31

I. General Information

General Experimental.

Unless otherwise noted, reactions were carried out in a 10 mL glass tube with magnetic stirring. Reactions that require heating were carried out in the oil bath. Analytical thin layer chromatography (TLC) was performed with Merck silica gel 60 F_{254} aluminum plates. Visualization was done under a UV lamp (254 nm) and by immersion in potassium permanganate ($KMnO_4$), followed by heating using a heat gun. Organic solutions were concentrated by rotary evaporation at 23-35 °C. Purification of reaction products were generally done by flash column chromatography with Silicycle 60-230 mesh silica gel.

Materials.

Halocarbon 125-Pentafluoroethane (Purity: 99.0% min., 9.1kg in 16 L size cylinder) was purchased from SCIENTIFIC GAS ENGINEERING CO., LTD. $Ir(ppy)_3$ (extra pure, 99.99%) was purchased from Bide. Anhydrous MeOH and anhydrous CH_3CN was purchased from J&K Scientific. $TESCF_2CF_3$ was prepared from HCF_2CF_3 according to the literature. Other chemicals for substrates preparation were purchased from Acros, J&K Scientific, Aldrich, Bide and Dikemann.

Instrumentation.

Proton nuclear magnetic resonance spectra (^1H NMR), carbon nuclear magnetic resonance spectra (^{13}C NMR) and fluorine nuclear magnetic resonance spectra (^{19}F NMR) were recorded at 23 °C on Bruker 400 MHz or 500 MHz spectrometer in $CDCl_3$. Chemical shifts of ^1H NMR spectra were reported as parts per million in δ scale using residual solvent signal ($CDCl_3$: 7.26 ppm) or tetramethylsilane (0.00 ppm) as internal standard. Chemical shifts of ^{13}C NMR spectra were reported using residual solvent signal of $CDCl_3$ (77.16 ppm) on the δ scale. Chemical shifts of 19F NMR were reported as parts per million in δ scale using benzotrifluoride (-63.72 ppm) as internal standard. Data are represented as follows: chemical shift (δ ppm), multiplicity (s = singlet, d = doublet, t = triplet, q = quartet, m = multiplet), coupling constant (*J*, Hz) and integration. High resolution mass spectra (HRMS) were obtained on a Finnigan MAT 95XL GC Mass Spectrometer or a Thermo Scientific Q Exactive Focus Mass Spectrometer or a Bruker SolariX 9.4T FTMS with Q Exactive Focus Orbitrap. X-ray structures were obtained on a Bruker Kappa ApexII Duo Diffractometer or a Bruker D8venture Diffractometer.

Photoreaction Setup.

All manipulations for the radical fluorosulfonylation via photoredox reactions were set up in a 10 mL Tube (unless otherwise noted) under an inert Argon atmosphere using glove-box techniques. The reactions were conducted in photo-reactors (Model:H106062, GEAO CHEMICAL, purchased from http://www.geaochem.com/), which comprise a fan for cooling (approximately room temperature) and six 1W blue LED beads for each place. The average power output of the photo-reactor was recorded at 30 mW/cm^2. One place/hole with three 1W blue LED beads (λ_{max} = 460 nm) on both side, and the distance between the Schlenk tube and LED beads is ca. 1 cm. The emission spectra of the blue LEDs were recorded on an Ocean Optics HR4000CG-UVNIR spectrometer. The spectra was normalised to 1.0 at the maximum (450 nm).

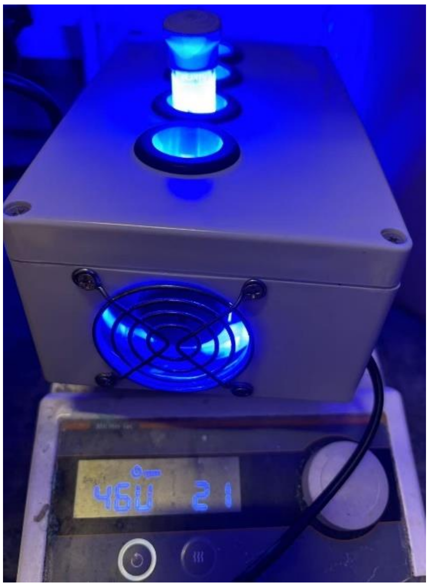

II. Experimental Procedures.

Procedures (I) for the synthesis of N-Tosyl-S-Pentafluoroethyl-S-sulfoximine (3)

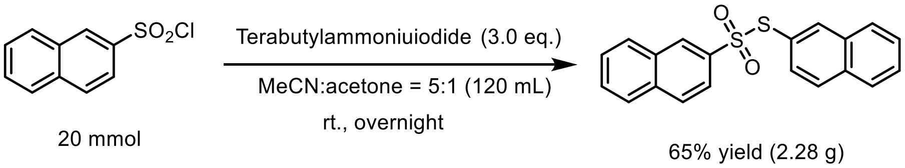

A round-bottom flask charged with a solution of sulfonyl chloride (20.0 mmol) in CH_3CN/acetone (50/10 mL). *n*-Bu_4NI (22.2 g, 3.0 mmol) in CH_3CN/acetone (50/10 mL) was added slowly by a syringe and the reaction system was allowed to stir at room temperature for 10 h. When the reaction was completed (monitored by TLC), the mixture was diluted in DCM and filtered through a pad of Celite. The residue was dried over Na_2SO_4 and the solvent was removed by reduced pressure. Purification on column chromatography afforded the corresponding product as a white solid (2.28 g, 65%). The purity of the product was determined by ^{1}H NMR. The spectra are in full accordance with the literature report.[1]

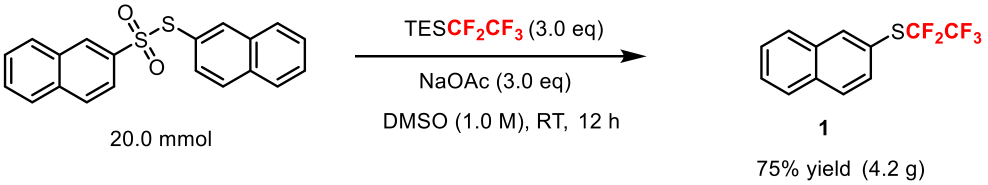

Pentafluoroethyl Sulfides was synthesized according to the literature procedure.[2] A round bottom flask charged with naphthyl thiosulfonate (7.00 g, 20 mmol) and NaOAc (4.92 g, 60 mmol) was evacuated and flushed with argon. 20 mL of DMSO was added, the reaction mixture was stirred at room temperature for 1 h under argon, then followed by dropwise addition of $TESCF_2CF_3$ (14.1 g, 60.0 mmol) under stirring. After 10 h of stirring when the reaction was completed (monitored by TLC), the mixture was diluted with DCM. The organic layer was washed with water (three times) and brine, then dried over anhydrous Na_2SO_4. After filtration, the filtrate was concentrated under reduced pressure. The residue was purified by flash chromatography on silica gel to afford the corresponding pentafluoroethyl sulfide as a colorless oil (15 mmol, 4.2 g, 75 %).

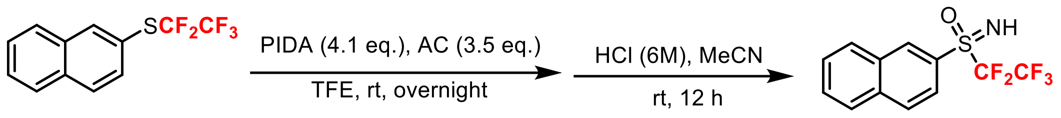

5.0 mmol

2

48% yield (742.6 mg)

S- pentafluoroethyl *N*H-sulfoximines (2) was synthesized according to the literature procedure.[3] To a round-bottom flask was added pentafluoroethyl sulfide (5.0 mmol, 1.0 equiv.) and trifluoroethanol (TFE, 0.4 M). Ammonium carbamate (AC, 1.5 equiv.) and diacetoxyiodobenzene (PIDA, 2.1 equiv.) were added in one portion. The reaction mixture was stirred at room temperature for 3 h. To reach maximum conversion (checked by GC-MS), PIDA (1.0 equiv.) and ammonium carbamate (1.0 equiv.) could be added. After completion, trifluoroethanol was removed under reduced pressure. The crude mixture was diluted in an aqueous solution of HCl (6 M, 1 mL / mmol) and CH_3CN (2 mL / mmol), and the reaction was stirred overnight at room temperature. The pH of the aqueous phase was adjusted to 7 with $NaHCO_3$ (10% aqueous solution) then the crude mixture was extracted with DCM (3 x 10 mL). The organic layer was dried with $MgSO_4$, concentrated under reduced pressure and purified by column chromatography on silica gel to afford the corresponding S-pentafluoroethyl *N*H-sulfoximine as a colorless oil (2.4 mmol, 742.6 mg, 48 %).

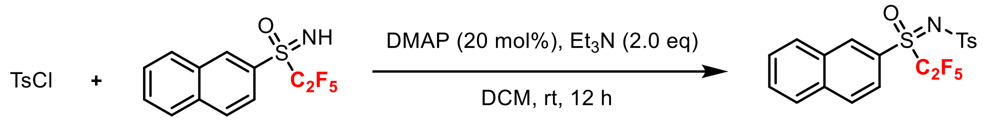

2.0 eq. 10.0 mmol

3

75% yield (3.5 g)

N-Tosyl-S-Pentafluoroethyl-S-sulfoximine (3) was synthesized according to the literature procedure.[3] A round-bottom flask was charged with S- pentafluoroethyl *N*H-sulfoximines (3.09 g, 10.0 mmol), tosyl chloride (3.82 g, 20 mmol) and DMAP (244.4 mg, 2.0 mmol). Then, 20 mL of DCM and 1.01 g of Et_3N (10 mmol) were added. The reaction mixture was stirred at room temperature for 12 h. When the reaction was completed (monitored by TLC), the reaction mixture was purified by flash chromatography on silica gel to afford the corresponding pentafluoroethyl sulfide as a yellow solid (7.5 mmol, 3.5 g, 75 %).

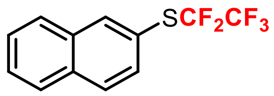

naphthalen-2-yl(perfluoroethyl)sulfane (1). The product was purified by flash column chromatography on silica gel using hexane as eluent and obtained as colorless oil (2.08 g, 75% yield, R_f = 0.7 (100% hexane).**^{1}H NMR** (500 MHz, $CDCl_3$): δ (ppm) 8.24 (s, 1H), 7.88 (d, J = 8.5 Hz, 3H), 7.68 (d, J = 8.5 Hz, 1H), 7.63 – 7.57 (m, 2H); **^{13}C NMR** (126 MHz, $CDCl_3$): δ (ppm) 138.2, 134.1, 133.5, 132.6, 129.3, 128.3, 128.2, 127.9, 127.2, 121.0 (tq, $J_{C\text{-}F}$ = 281.0, 32.7 Hz), 120.0 (t, $J_{C\text{-}F}$ = 2.9 Hz),119.0 (qt, $J_{C\text{-}F}$ = 286.5, 36.8 Hz); **^{19}F NMR** (471 MHz, $CDCl_3$): δ (ppm) -82.41 (t, J = 3.5 Hz, 3F), -91.56 (q, J = 3.5 Hz, 2F); **HRMS** m/z (ESI): calcd. for $C_{12}H_6F_5S$ $[M\text{-}H]^+$: 277.0116; found: 277.0119.

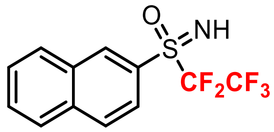

imino(naphthalen-2-yl)(perfluoroethyl)- λ ^{6}-sulfanone (2). The product was purified by flash column chromatography on silica gel using hexane : EA = 20:1 as eluent and obtained as colorless oil (742.6 mg, 48% yield, R_f = 0.55 (hexane : EA = 10:1).**^{1}H NMR** (500 MHz, $CDCl_3$): δ (ppm) 8.74 (s, 1H), 8.08 – 8.01 (m, 3H),

7.94 (d, J = 8.3 Hz, 1H), 7.71 (d, J = 7.6 Hz, 1H), 7.64 (d, J = 6.9 Hz, 1H), 4.01 (s, 1H); **^{13}C NMR** (126 MHz, $CDCl_3$): δ (ppm) 136.2, 133.8, 132.1, 130.4, 129.9, 129.6, 128.8, 128.1, 128.0, 124.5, 118.24 (qt, $J_{C\text{-}F}$ = 288.0, 32.8 Hz), 113.4 (ddq, $J_{C\text{-}F}$ = 304.8, 299.4, 38.7 Hz); **^{19}F NMR** (471 MHz, $CDCl_3$): δ (ppm) -77.51 (s, 3F), -113.19 (d, J = 251.5 Hz, 1F), -117.39 (d, J = 251.4 Hz, 1F); **HRMS** m/z (ESI): calcd. for $C_{12}H_7F_5NOS$ $[M\text{-}H]^+$: 308.0174; found: 308.0178.

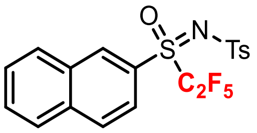

4-methyl-*N*-(naphthalen-2-yl(oxo)(perfluoroethyl)- λ^6-sulfaneylidene)benzenesulfonamide (3). The product was purified by flash column chromatography on silica gel using hexane : EA = 10:1 as eluent and obtained as yellow solid (3.47 g, 75% yield, R_f = 0.45 (hexane : EA = 5:1).**^{1}H NMR** (500 MHz, $CDCl_3$): δ (ppm) 8.73 (s, 1H), 8.10 (d, J = 8.7 Hz, 1H), 8.08 (d, J = 8.7 Hz, 1H), 8.03 – 7.99 (m, 2H), 7.87 (d, J = 8.0 Hz, 2H), 7.80 (d, J = 7.5 Hz, 1H), 7.72 (t, J = 7.5 Hz, 1H), 7.29 (d, J = 7.9 Hz, 2H), 2.41 (s,3H); **^{13}C NMR** (126 MHz, $CDCl_3$): δ (ppm) 144.0, 139.9, 136.7, 134.8, 132.1, 131.4, 130.4, 130.1, 129.6, 128.6, 128.2, 127.7, 126.7, 117.4 (qt, $J_{C\text{-}F}$ = 289.5, 32.1 Hz), 113.1 (ddq, $J_{C\text{-}F}$ = 307.4, 302.0, 40.0 Hz), 21.6; **^{19}F NMR** (471 MHz, $CDCl_3$): δ (ppm) -76.14 (s, 3F), -111.14 (d, J = 241.4 Hz, 1F), -114.50 (d, J = 241.8 Hz, 1F); **HRMS** m/z (ESI): calcd. for $C_{19}H_{14}F_5NO_3S_2Na$ $[M+Na]^+$: 486.0228; found: 486.0230.

Procedures (II) for the synthesis of N-Tosyl-S-trifluoromethyl-S-sulfoximine

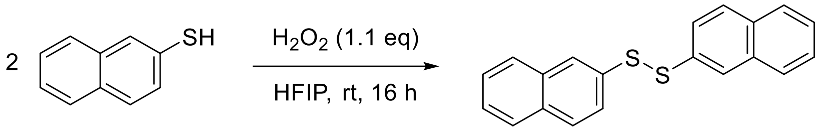

Under air, naphthyl thiol (31 mmol) was stirred in HFIP (30 mL). Then, H_2O_2 (1.1 equivalent, 34 mmol) was added and stirred at room temperature for 16 h. Then, the reaction medium was filtrated, and the solid was washed with water and ethanol, and dried under vacuum to give the expected product without further purification as a yellow powder (4.16 g, 13.1 mmol, 83%).

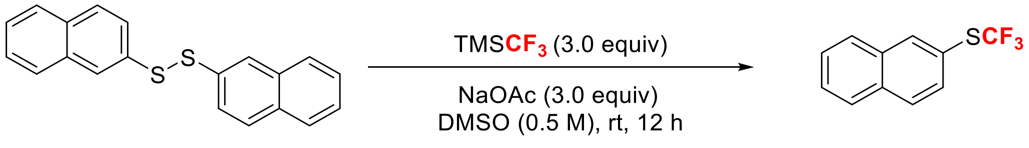

A round bottom flask charged with 1,2-di(naphthalen-2-yl)disulfane (4.34 g, 13.6 mmol) and NaOAc (3.35 g, 41 mmol) was evacuated and flushed with argon. 26 mL of DMSO were added (0.5 M), then $TMSCF_3$ was added dropwise (6.1 mL, 41 mmol) and the mixture was stirred during 16 h. The reaction medium was diluted with DCM. The organic layer was washed with water (three times) and brine, then dried over anhydrous $MgSO_4$. After filtration, the filtrate was concentrated under reduced pressure. The residue was purified by automated flash chromatography on silica gel using pure petroleum ether to afford the corresponding trifluoromethyl sulfide as a colorless oil (2.7 g, 88 %).

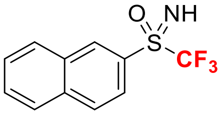

To a round-bottom flask was added naphthalen-2-yl(trifluoromethyl)sulfane (4.83 mmol, 1.0 equiv.) and trifluoroethanol (TFE, 0.4 M). Ammonium carbamate (AC, 1.5 equiv.) and diacetoxyiodobenzene (PIDA, 2.1 equiv.) were added in one portion. The reaction mixture was stirred at room temperature for 3 h. To reach maximum conversion, PIDA (1.0 equiv.) and ammonium carbamate (1.0 equiv.) could be added. After completion, trifluoroethanol was removed under reduced pressure. The crude was diluted in $NaHCO_3$ (saturated aqueous solution) then the crude mixture was extracted with DCM (3 x 10 mL). The organic layer was dried with $MgSO_4$, concentrated under reduced pressure and purified by column chromatography on silica gel to afford the corresponding imino(naphthalen-2-yl)(trifluoromethyl)- λ^6-sulfanone as a yellowish oil (930 mg, 74 %).

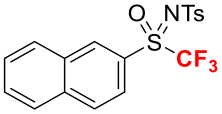

4-methyl-N-(naphthalen-2-yl(oxo)(trifluoromethyl)-l6-sulfaneylidene) benzenesulfonamide

A round-bottom flask was charged with imino(naphthalen-2-yl)(trifluoromethyl)- λ^6-sulfanone (3.2 g, 12.4 mmol), tosyl chloride (4.70 g, 25 mmol) and DMAP (303 mg, 2.5 mmol). Then, 124 mL of DCM and 3.45 mL of Et_3N (25 mmol) were added. The reaction mixture was stirred at room temperature for 12 h. When the reaction was completed (monitored by TLC), the reaction mixture was purified by automated flash chromatography on silica gel to afford the expected product as a yellow solid (4.8 g, 94 %).

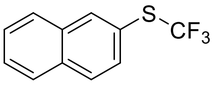

naphthalen-2-yl(trifluoromethyl)sulfane

^{1}H NMR (300 MHz, $CDCl_3$) δ: 8.22 (s, 1H), 7.95-7.82 (m, 3H), 7.69 (d, J = 8.7 Hz, 1H), 7.65-7.51 (m, 2H). **^{19}F NMR (282 MHz, $CDCl_3$) δ:** -43.0 (s, 1F). **^{13}C NMR (75 MHz, $CDCl_3$) δ:** 137.2, 134.0, 133.5, 131.9, 129.8 (q, J = 307 Hz), 129.4, 128.3, 128.1, 127.9, 127.1, 121.6 (q, J = 2.1 Hz). The physical and spectral data were consistent with previously reported: Oxidative Trifluoromethylthiolations of Aryl Boronic Acids Using a Copper/O2-Based protocol.

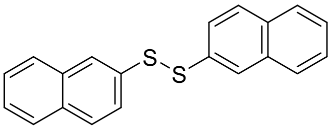

1,2-di(naphthalen-2-yl)disulfane

^{1}H NMR (300 MHz, *d*-DMSO) δ: 8.14 (d, J = 1.9 Hz, 2H), 7.95 (d, J = 8.6 Hz, 2H), 7. 90 (q, J = 5.1 Hz, 4H), 7.67 (dd, J = 8.8, 1.8 Hz, 2H), 7.57-7.47 (m, 4H). **^{13}C NMR (75 MHz, *d*-DMSO) δ:** 133.1, 133.0, 132.1, 129.3, 127.7, 127.4, 127.1, 126.6, 126.2, 125.3. The spectrum contains a lot of background noise due to poor solubility of the compound. The physical and spectral data were consistent with previously reported.: Metal-Free Photocatalytic Aerobic Oxidation of Thiols to Disulfides in Batch and Continuous-Flow.

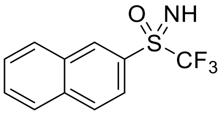

imino(naphthalen-2-yl)(trifluoromethyl)- λ6-sulfanone

Purified by automated column chromatography using petroleum ether/EtOAc 100:0 to 9:1 as an eluent. **^{1}H NMR (300 MHz, CDCl$_3$) *δ*:** 8.75 (s, 1H), 8.12-7.92 (m, 4H), 7.78-7.61 (m, 2H), 3.60 (bs, 1H). **^{19}F NMR (282 MHz, CDCl$_3$) *δ*:** -79.1 (s, 3F). **^{13}C NMR (75 MHz, CDCl$_3$) *δ*:** 136.2, 133.6, 132.2, 130.4, 129.9 (2C), 128.2, 128.1, 128.0, 124.3, 121.3 (q, J = 330 Hz). **HRMS (ESI+):** m/z [M + H]$^+$ calcd for $C_{11}H_9NOSF_3$: 260.0357; found: 260.0351.

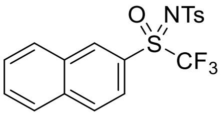

4-methyl-N-(naphthalen-2-yl(oxo)(trifluoromethyl)- λ6-sulfaneylidene) benzenesulfonamide

^{1}H NMR (300 MHz, CDCl$_3$) *δ*: 8.72 (s, 1H), 8.08 (t, J = 9.8 Hz, 2H), 7.99 (d, J = 8.1 Hz, 2H), 7.90 (d, J = 7.9 Hz, 2H), 7.84-7.67 (m, 2H), 7.30 (d, J = 8.1 Hz, 2H), 2.41 (s, 3H). **^{19}F NMR (282 MHz, CDCl$_3$) *δ*:** -75.2 (s, 3F). **^{13}C NMR (75 MHz, CDCl$_3$) *δ*:** 144.0, 139.9, 136.8, 134.3 (2C), 132.3, 131.3, 130.6, 130.1, 129.7 (2C), 128.6, 128.3, 127.0 (2C), 123.6, 120.4 (q, J = 329 Hz), 21.7. **HRMS (ESI+):** m/z [M + H]$^+$ calcd for $C_{18}H_{15}F_3NO_3S_2$: 414.0436; found: 414.0439.

General Procedure (III) for the synthesis of 5

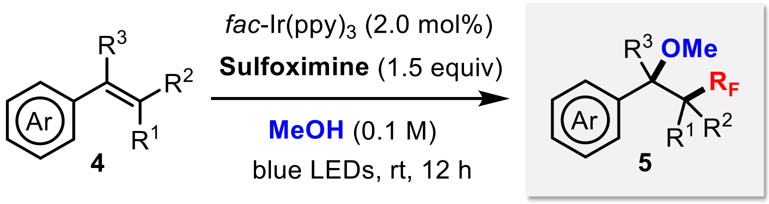

fac-Ir(ppy)$_3$ (2.60 mg, 0.004 mmol, 2 mol%) and sulfoximine reagent (0.3 mmol) were weighed into an oven-dried tube, followed by the addition of anhydrous MeOH (2.0 mL, 0.1 M) and alkene substrate **4** (0.2 mmol) under argon. The reaction mixture was allowed to stir at room temperature under irradiation with blue LEDs for 12 h. Purification by column chromatography or preparative thin layer chromatography on silica gel gave the desired pure product **5**. Photo-induced reactions were conducted in photo-reactors, which comprise a fan for cooling (approximately room temperature) and six 1W blue LED beads for each place (6 W). The average power output of the photo-reactor was ca. 30 mW/cm^2. The emission spectra of the blue LEDs were recorded on an Ocean Optics HR4000CG-UVNIR spectrometer.

General Procedure (IV) for the synthesis of 6

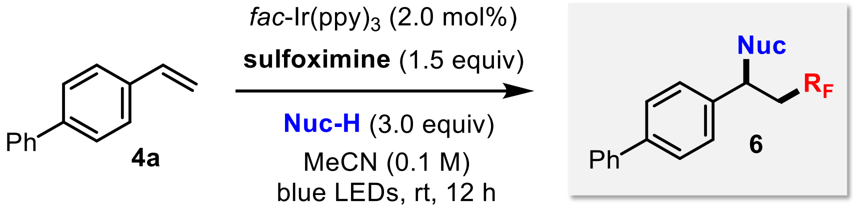

fac-Ir(ppy)$_3$ (2.60 mg, 0.004 mmol, 2 mol%) and sulfoximine reagent (0.3 mmol) were weighed into an oven-

dried tube, followed by the addition of anhydrous CH_3CN (2.0 mL, 0.1 M), alkene substrate **4a** (0.2 mmol) and nucleophile substrate (0.6 mmol, 3.0 equiv.) under argon. The reaction mixture was allowed to stir at room temperature under irradiation with blue LEDs for 12 h. Purification by column chromatography or preparative thin layer chromatography on silica gel gave the desired pure product **6**. Photo-induced reactions were conducted in photo-reactors, which comprise a fan for cooling (approximately room temperature) and six 1W blue LED beads for each place (6 W). The average power output of the photo-reactor was ca. 30 mW/cm^2. The emission spectra of the blue LEDs were recorded on an Ocean Optics HR4000CG-UVNIR spectrometer.

General Procedure (V) for the synthesis of 7

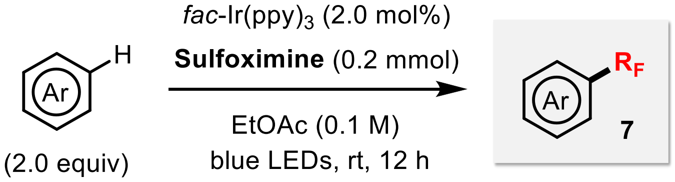

fac-Ir(ppy)$_3$ (2.60 mg, 0.004 mmol, 2 mol%) and sulfoximine reagent **3** (0.2 mmol) were weighed into an oven-dried tube, followed by the addition of anhydrous EtOAc (2.0 mL, 0.1 M), aromatic ring substrate (0.4 mmol, 2.0 equiv.) under argon. The reaction mixture was allowed to stir at room temperature under irradiation with blue LEDs for 12 h. Purification by column chromatography or preparative thin layer chromatography on silica gel gave the desired pure product **7**. Photo-induced reactions were conducted in photo-reactors, which comprise a fan for cooling (approximately room temperature) and six 1W blue LED beads for each place (6 W). The average power output of the photo-reactor was ca. 30 mW/cm^2. The emission spectra of the blue LEDs were recorded on an Ocean Optics HR4000CG-UVNIR spectrometer.

Procedure (VI) for the synthesis of cyclopropyl(phenyl)methanone 8

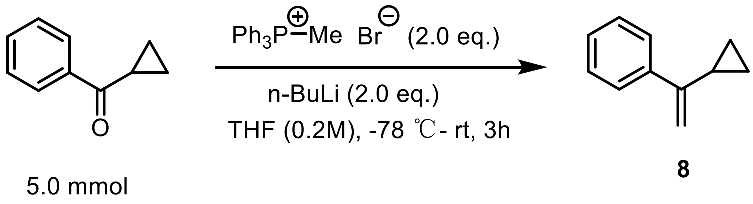

An oven dried Schlenk flask was charged with methyltriphenylphosphonium bromide (3.57 g, 10 mmol, 2.0 equiv.), and placed under argon atmosphere. Anhydrous THF (30 mL) was added and the suspension was cooled to -78°C. While stirring, *n*-BuLi (1.6 M in hexanes, 6.25 mL, 10 mmol, 2.0 equiv.) was added dropwise. After 10 minutes of stirring, cyclopropyl phenyl ketone (690 μl, 5.0 mmol, 1.0 equiv.) was added dropwise. After stirring of the solution for three hours at room temperature, the reaction mixture was quenched by the addition of NH_4Cl solution (satd.). The reaction mixture was transferred to a separatory funnel, and the phases were separated. The aqueous layer was extracted three times with hexane (10 mL each time). The combined organic layers were dried with Na_2SO_4, filtered and the solvent was removed in vacuo. Purification by column chromatography on silica gel gave **8** as colorless oil (641 mg, 4.45 mmol, 89%). The purity of the product was determined by ^1H NMR. The spectra are in full accordance with the literature report.[5]

Procedure (VII) for the synthesis of $Et_3SiCF_2CF_3$

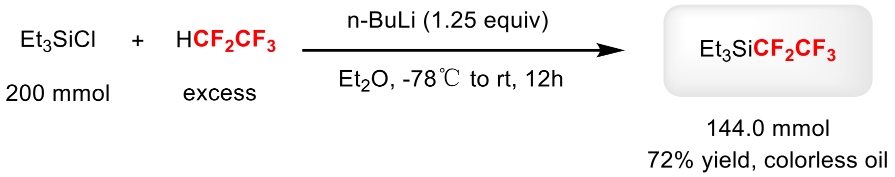

$Et_3SiCF_2CF_3$ was synthesized according to the literature procedure.[4] In a round-bottom flask, 250 ml of diethyl ether was cooled down to -78 °C. Pentafluoroethane was bubbled into diethyl ether for 3 hours followed by the careful addition of a solution of *n*-BuLi (100 ml, 2.5 M in hexane, 250 mmol, 1.25 equiv), so that the temperature of the reaction system didn't exceed -60 °C. After stirring for 1 h, a solution of triethylchlorosilane (21.72 g, 200 mmol, 1.0 equiv) in diethyl ether was added within 5 min. The mixture was allowed to warm up to room temperature slowly for 12 h. The mixture was filtered through a pad of Celite and concentrated to dryness to afford the crude product. The product was purified by distillation in vacuo and obtained as a colorless liquid (33.8 g, 72%). The purity of the product was determined by ^1H NMR and ^{19}F NMR. The spectra are in full accordance with the literature report.[4]

III. Control Experiments

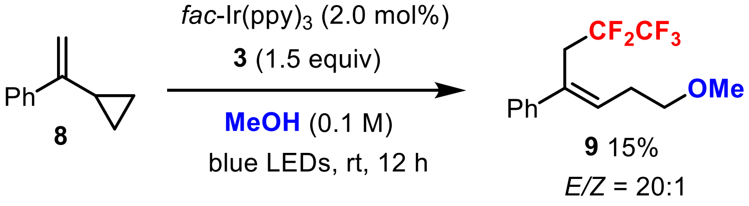

fac-Ir(ppy)$_3$ (2.60 mg, 0.004 mmol, 2 mol%) and radical reagent **3** (138.9 mg, 0.3 mmol) were weighed into an oven-dried tube, followed by the addition of anhydrous MeOH (2.0 mL, 0.1 M) and Cyclopropyl(phenyl)methanone **8** (0.2 mmol) under argon. The reaction mixture was allowed to stir at room temperature under irradiation with blue LEDs for 12 h. Purification by column chromatography or preparative thin layer chromatography on silica gel gave the desired pure product **9**. Photo-induced reactions were conducted in photo-reactors, which comprise a fan for cooling (approximately room temperature) and six 1W blue LED beads for each place (6 W). The average power output of the photo-reactor was ca. 30 mW/cm^2. The emission spectra of the blue LEDs were recorded on an Ocean Optics HR4000CG-UVNIR spectrometer.

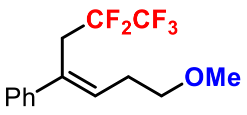

(*E*)-(6,6,7,7,7-pentafluoro-1-methoxyhept-3-en-4-yl)benzene (9). The product was purified by flash column chromatography on silica gel using hexane : EA = 20:1 as eluent and obtained as colorless oil (8.8 mg, 15% yield, dr = 20:1, R_f = 0.45 (hexane : EA = 10:1).**^1H NMR** (500 MHz, $CDCl_3$): δ (ppm) 7.37 – 7.33 (m, 2H), 7.29 – 7.26 (m, 1H), 7.19 (d, J = 6.7 Hz, 2H), 5.81 (t, J = 7.2 Hz, 1H), 3.38 (t, J = 6.5 Hz, 2H), 3.29 (s, 3H), 3.10 (t, J = 17.9 Hz, 2H), 2.31 (q, J = 6.8 Hz, 2H); **^{13}C NMR** (126 MHz, $CDCl_3$): δ (ppm) 139.5, 132.4, 131.1 (t, $J_{C\text{-}F}$ = 2.2 Hz), 128.5, 128.4, 127.4, 119.2 (qt, $J_{C\text{-}F}$ = 286.0, 36.2 Hz), 115.0 (tq, $J_{C\text{-}F}$ = 253.6, 36.9 Hz), 72.0, 58.7, 40.0 (t, $J_{C\text{-}F}$ =

21.4 Hz), 29.8, 17.3, 11.8; **^{19}F NMR** (471 MHz, $CDCl_3$): δ (ppm) -85.05 (s, 3F), -115.95 (t, J = 17.8 Hz, 2F); **HRMS** m/z (ESI): calcd. for $C_{14}H_{15}F_5ONa$ $[M+Na]^+$: 317.0935; found: 317.0937.

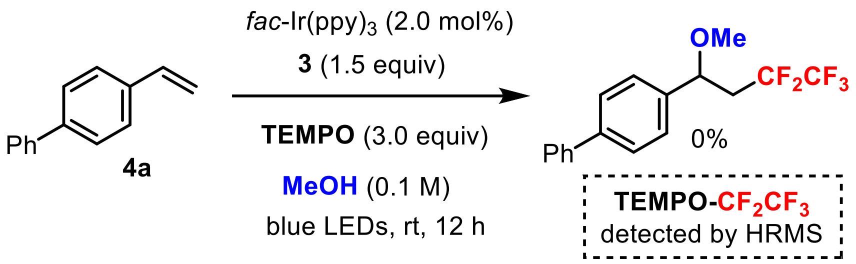

fac-Ir(ppy)$_3$ (2.60 mg, 0.004 mmol, 2 mol%), radical reagent **3** (138.9 mg, 0.3 mmol), TEMPO (0.6 mmol, 3.0 equiv.) were weighed into an oven-dried tube, followed by the addition of anhydrous MeOH (2.0 mL, 0.1 M) and alkene substrate **4a** (0.2 mmol) under argon. The reaction mixture was allowed to stir at room temperature under irradiation with blue LEDs for 12 h. Photo-induced reactions were conducted in photo-reactors, which comprise a fan for cooling (approximately room temperature) and six 1W blue LED beads for each place (6 W). The average power output of the photo-reactor was ca. 30 mW/cm^2. The emission spectra of the blue LEDs were recorded on an Ocean Optics HR4000CG-UVNIR spectrometer. TEMPO-CF_2CF_3 was checked by ^{19}F NMR and HRMS. **2,2,6,6-tetramethyl-1-(perfluoroethoxy)piperidine: ^{19}F NMR** (471 MHz, $CDCl_3$): δ (ppm) -84.91 (s, 2F), -85.55 (s, 3F); **HRMS** m/z (APCI): calcd. for $C_{11}H_{19}F_5NO$ $[M+H]^+$: 276.13813; found: 276.13854.

IV. Optimization Studies

Table S1. Using MeOH as solvent.

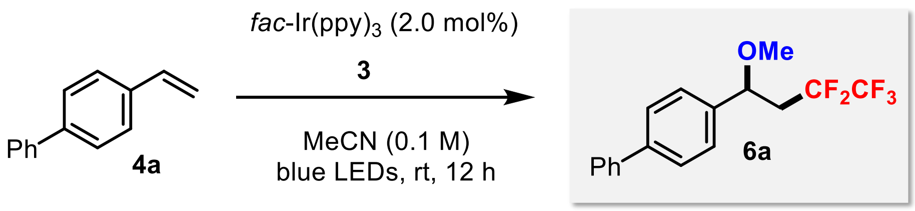

Entry	**4a**	**3**	Solvent	Yield of **5a** [b]
1	2.0eq.	1.0 eq.	MeOH	38%
2	1.5 eq.	1.0 eq	MeOH	70%
3	1.0 eq.	1.0 eq.	MeOH	63%
**4**	**1.0 eq**	**1.5 eq.**	**MeOH**	**85%**
5	1.0 eq.	2.0 eq.	MeOH	54%

[a] Unless specified otherwise. Reaction conditions: **4a**, **3**, Photocatalyst (2.0 mol%), argon, blue LEDs (λ_{max} = 460 nm), rt, 12 h. [b] Yield was determined by ^{19}F NMR analysis using benzotrifluoride as the internal standard.

Table S2. Screening of photocatalysts and solvents.

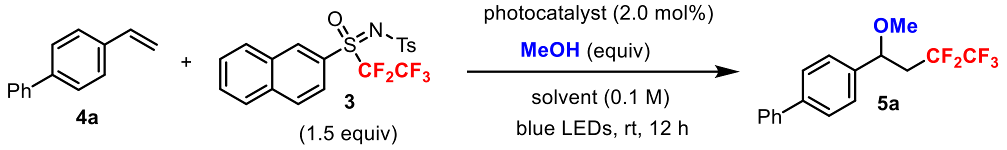

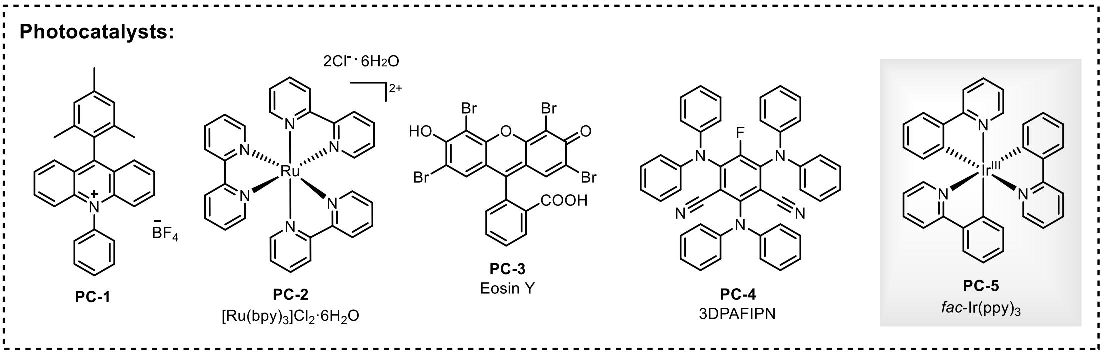

Entry	Photocatalyst	Solvent	MeOH (equiv.)	Yield of **3a**[b]
1	PC-1	DCM	3.0	10%
2	$Ru(bpy)_3Cl_2$•$6H_2O$	DCM	3.0	trace
3	Eosin Y	DCM	3.0	trace
4	3DPAFIPN	DCM	3.0	trace
5	*fac*-Ir(ppy)$_3$	DCM	3.0	71%
6	*fac*-Ir(ppy)$_3$	Acetone	3.0	36%
7	*fac*-Ir(ppy)$_3$	DMF	3.0	trace
8	*fac*-Ir(ppy)$_3$	THF	3.0	24%
9	*fac*-Ir(ppy)$_3$	EA	3.0	10%
10	*fac*-Ir(ppy)$_3$	CH_3CN	5.0	80%
11	*fac*-Ir(ppy)$_3$	CH_3CN	4.0	82%
**12**	***fac*-Ir(ppy)$_3$**	**CH_3CN**	**3.0**	**90%**
13	*fac*-Ir(ppy)$_3$	CH_3CN	2.0	86%
14	*fac*-Ir(ppy)$_3$	CH_3CN	1.5	73%
15	*fac*-Ir(ppy)$_3$	CH_3CN	1.0	59%

[a] Unless specified otherwise. Reaction conditions: **4a** (0.2 mmol), **3** (0.3 mmol, 1.5 equiv.), Photocatalyst (2.0 mol%), argon, blue LEDs (λ_{max} = 460 nm), rt, 12 h. [b] Yield was determined by ^{19}F NMR analysis using benzotrifluoride as the internal standard.

Table S3. C-H bond pentafluoroethylation of an arene.

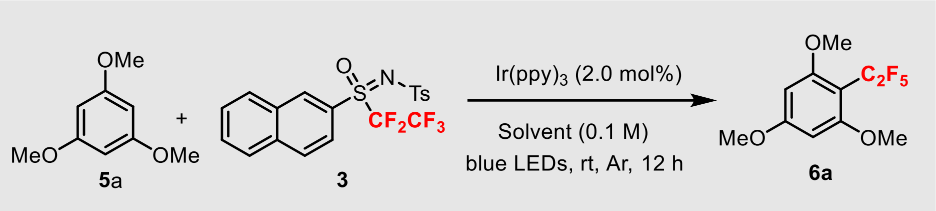

Entry	Solvent	**5a** (equiv.)	**2** (equiv.)	Yield of **6a**[b]
1	CH_3CN	1.0	1.5	62%
2	DMF	1.0	1.5	70%
3	DMSO	1.0	1.5	77%
4	THF	1.0	1.5	trace
5	DCM	1.0	1.5	57%
6	EA	1.0	1.5	82%
7	EA	1.0	1.0	66%
8	EA	1.5	1.0	67%
9	EA	2.0	1.0	88%
10	EA	2.5	1.0	85%

[a] Unless specified otherwise. Reaction conditions: **5a** (0.2 mmol), **3** (0.3 mmol, 1.5 equiv.), PC (2.0 mol%), argon, blue LEDs (λ_{max} = 460 nm), rt, 12 h. [b] Yield was determined by ^{19}F NMR analysis using benzotrifluoride as the internal standard.

V. Single Crystal X-Ray Structure Determinations of Compound 3

Method of crystallization: Crystals of **3** were obtained by slow diffusion from the solution in DCM layered MeOH. Crystallographic data for **3** has been deposited with the Cambridge Crystallographic Data Centre (CCDC) under deposition number numbers 2466731.

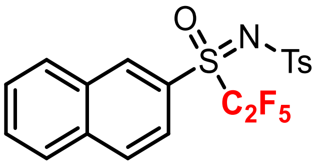

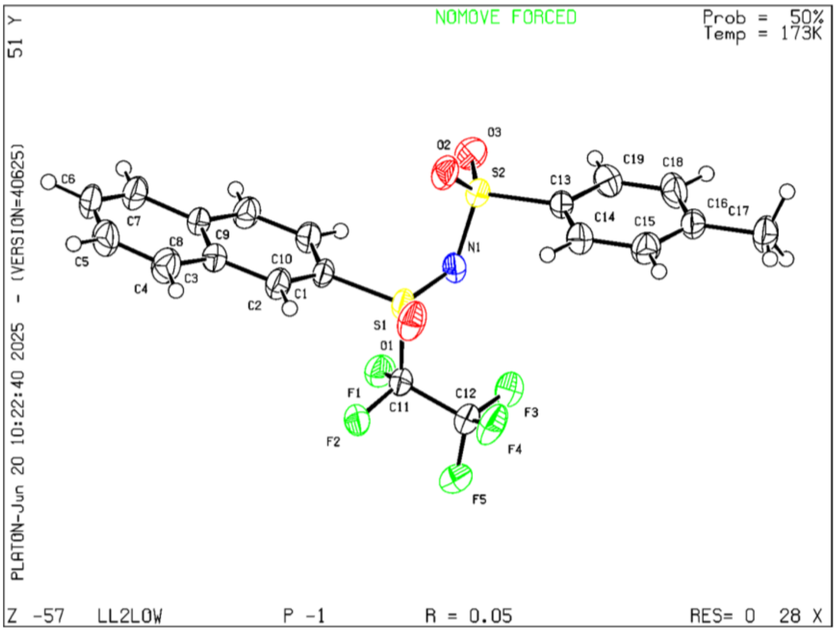

Table 1. Crystal data and structure refinement for p.

Identification code	CCDC:2466731	
Empirical formula	$C_{19} H_{14} F_5 NO_3S_2$	
Formula weight	463.43	
Temperature	173(2) K	
Wavelength	0.71073 Å	
Crystal system	Triclinic	
Space group	P-1	
Unit cell dimensions	a = 7.8599(5) Å	α= 93.174(2)°.
	b = 8.3997(5) Å	β= 93.802(2)°.
	c = 16.7247(12) Å	γ = 117.189(2)°.
Volume	975.43(11) $Å^3$	
Z	2	
Density (calculated)	1.578 Mg/m^3	
Absorption coefficient	0.342 mm^{-1}	

F(000)	472
Crystal size	0.400 x 0.300 x 0.200 mm^3
Theta range for data collection	3.057 to 28.318°.
Index ranges	-10<=h<=10, -11<=k<=11, -22<=l<=22
Reflections collected	43707
Independent reflections	4854 [R(int) = 0.0361]
Completeness to theta = 25.242°	99.5 %
Absorption correction	multi-scan
Max. and min. transmission	0.7457 and 0.7048
Refinement method	Full-matrix least-squares on F^2
Data / restraints / parameters	4854 / 0 / 271
Goodness-of-fit on F^2	1.062
Final R indices [I>2sigma(I)]	R1 = 0.0472, wR2 = 0.1480
R indices (all data)	R1 = 0.0555, wR2 = 0.1590
Extinction coefficient	n/a
Largest diff. peak and hole	0.977 and -0.385 e.$Å^{-3}$

VI. Comparison studies between pentafluoroethyl sulfoximine reagent and *trifluoromethyl* sulfoximine reagents.

(a)

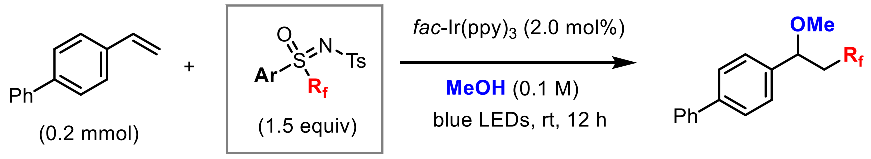

Ar = **2-naphthyl**, R_f = **CF_2CF_3**: 90% iso. yield
Ar = **2-naphthyl**, R_f = **CF_3**: 89% iso. yield
Ar = **phenyl**, R_f = **CF_3**: 93% iso. yield

(b)

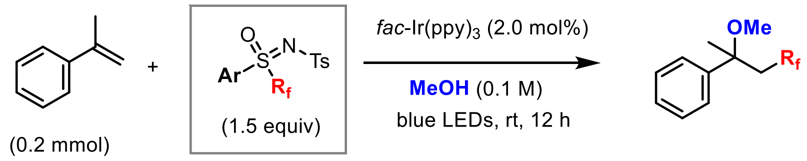

Ar = **2-naphthyl**, R_f = **CF_2CF_3**: 78% iso. yield
Ar = **2-naphthyl**, R_f = **CF_3**: 76% iso. yield

(c)

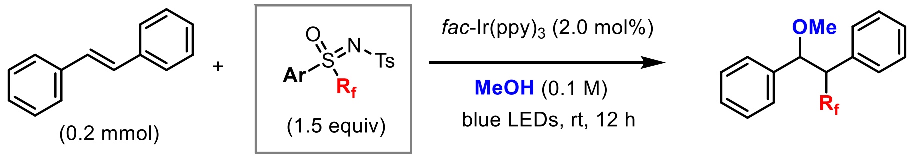

Ar = **2-naphthyl**, R_f = **CF_2CF_3**: 60% iso. yield (dr = 3:1)
Ar = **2-naphthyl**, R_f = **CF_3**: 64% iso. yield (dr = 5:1)

(d)

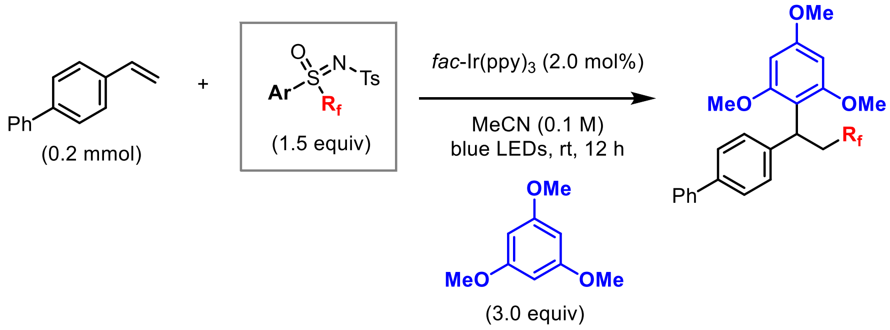

Ar = **2-naphthyl**, R_f = **CF_2CF_3**: 83% iso. yield
Ar = **2-naphthyl**, R_f = **CF_3**: 65% iso. yield
Ar = **phenyl**, R_f = **CF_3**: 73% iso. yield

(e)

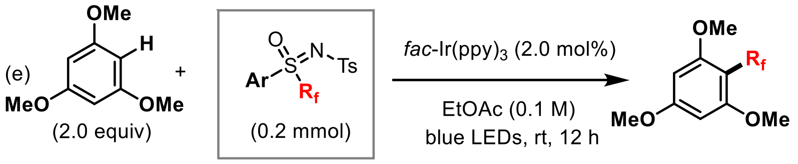

Ar = **2-naphthyl**, R_f = **CF_2CF_3**: 85% iso. yield
Ar = **2-naphthyl**, R_f = **CF_3**: 77% iso. yield
Ar = **phenyl**, R_f = **CF_3**: 83% iso. yield

VII. Stern-Volmer experiments

Rate of quenching (k_q) was determined using Stern-Volmer kinetics:

$$\frac{I_0}{I} = k_q \tau_0 [quencher] + 1$$

Where
I_0 is the luminescence intensity without the quencher
I is the intensity in presence of quencher
τ_0 is the excited state lifetime of the photocatalyst (τ_0 = 1.9 µs for $Ir(ppy)_3$)
The following stock solutions were prepared in distilled MeCN and degassed by three freeze-pump-thaw cycles.
General procedure: A stock solution of $Ir(ppy)_3$ was prepared by dissolving $Ir(ppy)_3$ (12.5 µmol) in 5 mL of MeCN. Of this solution, 0.4 mL were further diluted with the solvent to give a total volume of 10 mL. [**Ir**] = 0.1 x 10^{-3} M. A stock solution of pentafluoroethyl sulfoximine reagent **3** was prepared by dissolving **3** (125 µmol) in 5 mL of MeCN. [**3**] = 25 x 10^{-3} M. A stock solution of vinyl biphenyl **4a** was prepared by dissolving **4a** (125 µmol) in 5 mL of MeCN. [**4a**] = 25 x 10^{-3} M. For each experiment, 6 samples were prepared. Quartz cuvettes (3.5 mL) were filled with photocatalyst stock solution (0.2 mL), reagent stock solution (0 mL, 0.05 mL, 0.1 mL, 0.15 mL, 0.2 mL, 0.25 mL) and MeCN (2.8 mL, 2.75 mL, 2.7 mL, 2.65 mL, 2.6, 2.55 mL) to obtain a total volume of 3 mL. The final concentrations were [**Ir**] = 0.67 10^{-5} M and [quencher] = 0.42 x 10^{-3} M, 0.83 x 10^{-3} M, 1.25 x 10^{-3} M, 1.65 x 10^{-3} M and 2.08 x 10^{-3} M respectively. For each sample, emission spectra were acquired between 450 nm and 650 nm (excitation at 440 nm). In Figure S1 is plotted I_0/I where I is the maximum absorption intensity of iridium.

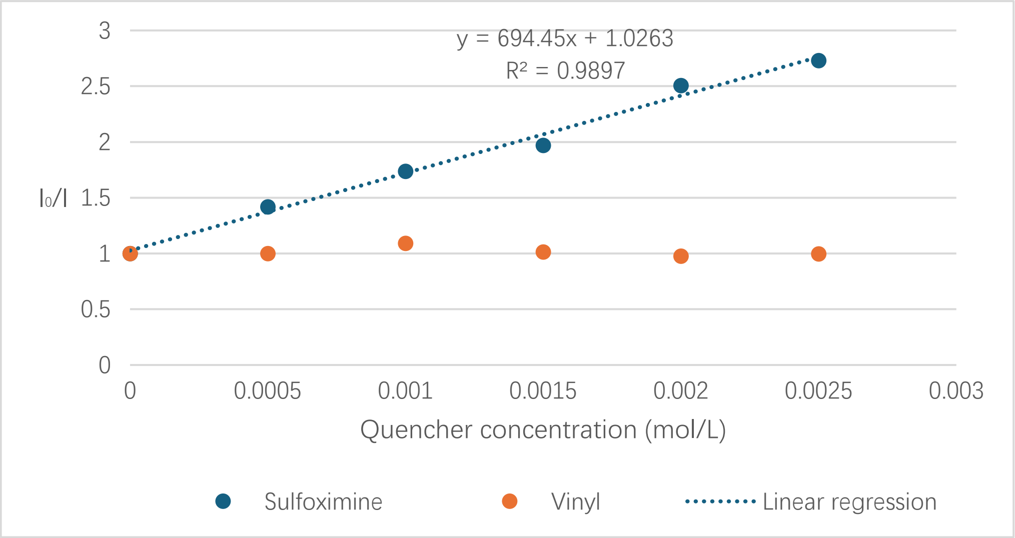

Figure S1: Stern-Volmer experiment: test of the quenching of luminescence of the iridium complex by the species involved in the reaction media

For pentafluoroethyl sulfoximine reagent **3** in MeCN:

$$\boldsymbol{k_q = 366 * 10^6\, L.mol^{-1}.s^{-1}}$$

VIII. Reduction potential measurement

Supporting electrolyte [Bu_4N][PF_6] and the corresponding sulfoximine were dissolved at 0.01 M in dry MeCN. Solutions were deaereated with argon. The potential of the reference electrode was determined by measuring its difference to the ferrocene/ferrocenium couple, used as external standard (+0.64 V in MeCN vs. aqueous SHE), and was then converted vs. SCE (+0.4 V). Cyclic voltammograms were collected with a OrigaFlex-OGF500 Potentiostat. The working electrode is a polished 3 mm diameter glassy carbon disk, the reference electrode is Ag/Ag^+, and the counter electrode a glassy carbon plate. The voltammetric scan rate was 100 $mV.s^{-1}$. Initial potential of scan was 0 V, starting with reduction potentials.

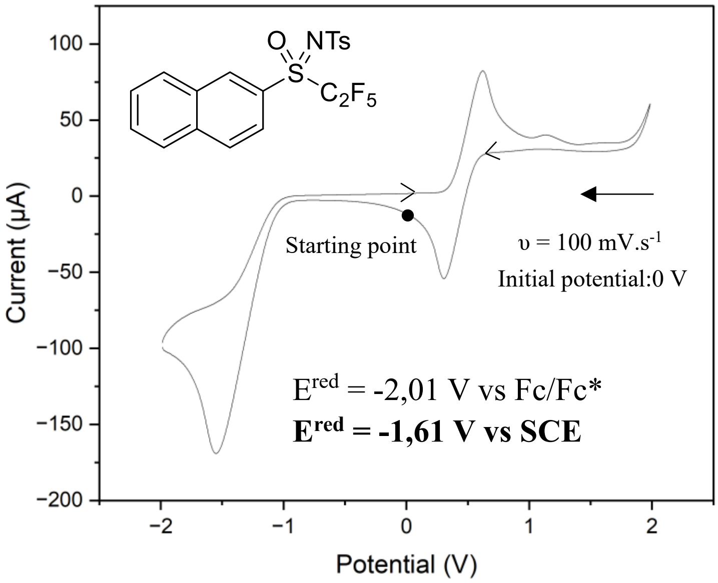

Figure S2: Cyclic voltammetry of pentafluoroethyl sulfoximine reagent **3** follows the IUPAC convention.

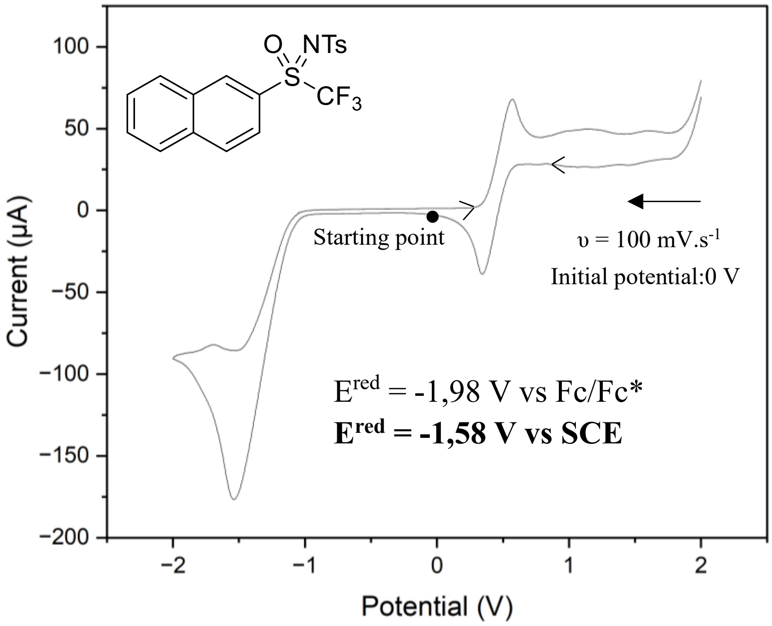

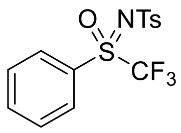

-1.73 V

Figure S3: Cyclic voltammetry of trifluoromethyl sulfoximine reagent follows the IUPAC convention.

IX. Characterization Data of Products:

4-(3,3,4,4,4-pentafluoro-1-methoxybutyl)-1,1'-biphenyl (5a)

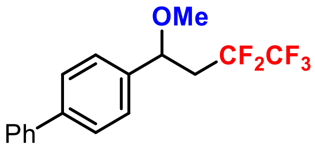

Following the general procedure (III). The product was purified by flash column chromatography on silica gel using hexane: EA=40:1 as eluent and obtained as colorless oil (59.4 mg, 90% yield, R_f = 0.45 (hexane: EA= 20:1).^{1}**H NMR** (500 MHz, $CDCl_3$): δ (ppm) 7.62 (t, J = 8.9 Hz, 4H), 7.46 (t, J = 7.6 Hz, 2H), 7.42 – 7.36 (m, 3H), 4.60 (dd, J = 8.7, 3.6 Hz, 1H), 3.28 (s, 3H), 2.71 – 2.59 (m, 1H), 2.39 – 2.29 (m, 1H); ^{13}**C NMR** (126 MHz, $CDCl_3$): δ (ppm) 141.4, 140.6, 139.3, 128.9, 127.6, 127.5, 127.1, 126.9, 119.1 (qt, $J_{C\text{-}F}$ = 285.5, 36.0 Hz), 114.7 (tq, $J_{C\text{-}F}$ = 254.2, 37.8 Hz), 76.6 (t, $J_{C\text{-}F}$ = 3.0 Hz), 56.7, 39.4 (t, $J_{C\text{-}F}$ = 20.8 Hz); ^{19}**F NMR** (471 MHz, $CDCl_3$): δ (ppm) -85.68 (s, 3F), -116.14 (ddd, J = 268.5, 23.1, 13.6 Hz, 1F), -116.82 (ddd, J = 268.5, 22.2, 12.7 Hz, 1F); **HRMS** m/z (APCI): calcd. for $C_{16}H_{12}F_5$ [M-CH_3OH+H]$^+$: 299.0853; found: 299.0853.

(3,3,4,4,4-pentafluoro-1-methoxybutyl)benzene (5b)

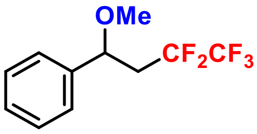

Following the general procedure (III). The product was purified by flash column chromatography on silica gel using hexane: EA= 40:1 as eluent and obtained as colorless oil (33.0 mg, 65% yield, R_f = 0.40 (hexane: EA= 20:1). ^{1}**H NMR** (500 MHz, $CDCl_3$): δ (ppm) 7.41 – 7.38 (m, 2H), 7.35 – 7.33 (m, 3H), 4.54 (dd, J = 8.7, 3.6 Hz, 1H), 3.23 (s, 3H), 2.66 – 2.54 (m, 1H), 2.34 – 2.23 (m, 1H); ^{13}**C NMR** (126 MHz, $CDCl_3$): δ (ppm) 140.5, 129.0, 128.6, 126.6, 119.2 (qt, $J_{C\text{-}F}$ = 285.3, 35.9 Hz), 114.9 (tq, $J_{C\text{-}F}$ = 254.5, 37.8 Hz), 56.8, 39.4 (t, $J_{C\text{-}F}$ = 20.8 Hz); ^{19}**F NMR** (471 MHz, $CDCl_3$): δ (ppm) -85.73 (s, 3F), -116.21 (ddd, J = 263.8, 23.3, 13.3 Hz, 1F), -116.90 (ddd, J = 268.5, 22.2, 12.5 Hz, 1F); **HRMS** m/z (APCI): calcd. for $C_{10}H_8F_5$ [M-CH_3OH+H]$^+$: 223.0541; found: 223.0542.

1-methyl-4-(3,3,4,4,4-pentafluoro-1-methoxybutyl)benzene (5c)

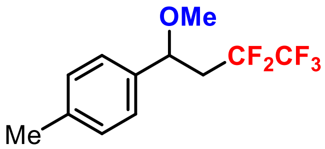

Following the general procedure (III). The product was purified by flash column chromatography on silica gel using hexane: EA=40:1 as eluent and obtained as colorless oil (42.9 mg, 80% yield, R_f = 0.40 (hexane: EA= 20:1). ^{1}**H NMR** (500 MHz, $CDCl_3$): δ (ppm) 7.23 – 7.19 (m, 4H), 4.51 (dd, J = 8.7, 3.6 Hz, 1H), 3.22 (s, 3H), 2.65 – 2.53 (m, 1H), 2.37 (s, 3H), 2.33 – 2.25 (m, 1H); ^{13}**C NMR** (126 MHz, $CDCl_3$): δ (ppm) 138.2, 137.3, 129.5, 126.4, 119.1 (qt, $J_{C\text{-}F}$ = 285.3, 35.9 Hz), 114.8 (tq, $J_{C\text{-}F}$ = 253.7, 38.0 Hz), 76.6 (t, $J_{C\text{-}F}$ = 3.1 Hz), 56.5, 39.3 (t, $J_{C\text{-}F}$ = 20.7 Hz), 21.1; ^{19}**F NMR** (471 MHz, $CDCl_3$): δ (ppm) -85.76 (s, 3F), -116.21 (ddd, J = 263.8, 23.8, 13.4 Hz, 1F), -116.94 (ddd, J = 268.5, 22.6, 12.2 Hz, 1F); **HRMS** m/z (APCI): calcd. for $C_{11}H_{10}F_5$ [M-CH_3OH+H]$^+$: 237.0697; found: 237.0698.

1-methoxy-4-(3,3,4,4,4-pentafluoro-1-methoxybutyl)benzene (5d)

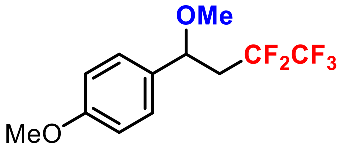

Following the general procedure (III). The product was purified by flash column chromatography on silica gel using hexane: EA= 40:1 as eluent and obtained as colorless oil (50.0 mg, 88% yield, R_f = 0.50 (hexane: EA= 20:1). ^{1}**H NMR** (500 MHz, $CDCl_3$): δ (ppm) 7.29 – 7.26 (m, 2H), 6.96 – 6.92 (m, 2H), 4.52 (dd, J = 8.5, 3.8 Hz, 1H), 3.85 (s, 3H), 3.22 (s, 3H), 2.68 – 2.56 (m, 1H), 2.36 – 2.25 (m, 1H); ^{13}**C NMR** (126 MHz, $CDCl_3$): δ (ppm) 159.8, 132.4, 127.8, 119.2 (qt, $J_{C\text{-}F}$ = 285.5, 35.9 Hz), 114.9 (tq, $J_{C\text{-}F}$ = 253.7, 37.8 Hz), 114.3, 76.5 (t, $J_{C\text{-}F}$ = 3.1 Hz), 56.4, 55.4, 39.4 (t, $J_{C\text{-}F}$ = 20.8 Hz); ^{19}**F NMR** (471 MHz, $CDCl_3$): δ (ppm) -85.79 (s, 3F), -116.24 (ddd, J = 263.8, 23.7, 13.5 Hz, 1F), -116.95 (ddd, J = 268.5, 22.3, 12.5 Hz, 1F); **HRMS** m/z (ESI): calcd. for $C_{12}H_{13}F_5O_2Na$ $[M+Na]^+$: 307.0728; found: 307.0730.

methyl 4-(3,3,4,4,4-pentafluoro-1-methoxybutyl)benzoate (5e)

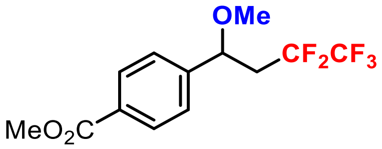

Following the general procedure (III). The product was purified by flash column chromatography on silica gel using hexane: EA= 40:1 as eluent and obtained as colorless oil (33.7 mg, 54% yield, R_f = 0.30 (hexane: EA= 20:1). ^{1}**H NMR** (500 MHz, $CDCl_3$): δ (ppm) 8.06 (d, J = 8.2 Hz, 2H), 7.41 (d, J = 8.2 Hz, 2H), 4.58 (dd, J = 8.5, 3.7 Hz, 1H), 3.92 (s, 3H), 3.23 (s, 3H), 2.65 – 2.53 (m, 1H), 2.33 – 2.22 (m, 1H); ^{13}**C NMR** (126 MHz, $CDCl_3$): δ (ppm) 166.8, 145.6, 130.5, 130.3, 126.6, 116.1 (qt, $J_{C\text{-}F}$ = 285.6, 35.8 Hz), 114.7 (tq, $J_{C\text{-}F}$ = 253.7, 37.8 Hz), 76.6 (t, $J_{C\text{-}F}$ = 3.0 Hz), 57.0, 52.3, 39.3 (t, $J_{C\text{-}F}$ = 20.9 Hz); ^{19}**F NMR** (471 MHz, $CDCl_3$): δ (ppm) -85.72 (s, 3F), -116.42 (t, J = 17.8 Hz, 2F). **HRMS** m/z (APCI): calcd. for $C_{13}H_{14}F_5O_3$ $[M+H]^+$: 313.0858; found: 313.0859.

1-chloro-4-(3,3,4,4,4-pentafluoro-1-methoxybutyl)benzene (5f)

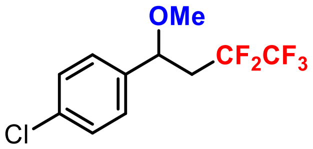

Following the general procedure (III). The product was purified by flash column chromatography on silica gel using hexane: EA= 40:1 as eluent and obtained as colorless oil (43.8 mg, 76% yield, R_f = 0.55 (hexane: EA= 20:1). ^{1}**H NMR** (500 MHz, $CDCl_3$): δ (ppm) 7.38 – 7.35 (m, 2H), 7.29 – 7.25 (m, 2H), 4.51 (dd, J = 8.5, 3.9 Hz, 1H), 3.21 (s, 3H), 2.64 – 2.51 (m, 1H), 2.31 – 2.20 (m, 1H); ^{13}**C NMR** (126 MHz, $CDCl_3$): δ (ppm) 139.0, 134.4, 129.2, 128.0, 119.1 (qt, $J_{C\text{-}F}$ = 285.4, 35.8 Hz), 114.7 (tq, $J_{C\text{-}F}$ = 253.7, 37.8 Hz), 76.4 (t, $J_{C\text{-}F}$ = 3.1 Hz), 56.8, 39.3 (t, $J_{C\text{-}F}$ = 20.9 Hz); ^{19}**F NMR** (471 MHz, $CDCl_3$): δ (ppm) -85.75 (s, 3F), -116.44 – -116.52 (m, 2F). **HRMS** m/z (APCI): calcd. for $C_{10}H_7ClF_5$ $[M-CH_3OH+H]^+$: 257.0151; found: 257.0151.

1-bromo-2-(3,3,4,4,4-pentafluoro-1-methoxybutyl)benzene (5g)

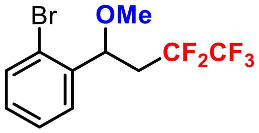

Following the general procedure (III). The product was purified by flash column chromatography on silica gel using hexane: EA= 40:1 as eluent and obtained as colorless oil (49.7 mg, 75% yield, R_f = 0.45 (hexane: EA= 20:1). **^{1}H NMR** (500 MHz, $CDCl_3$): δ (ppm) 7.57 (dd, J = 7.9, 1.1 Hz, 1H), 7.51 (dd, J = 7.7, 1.7 Hz, 1H), 7.40 – 7.37 (m, 1H), 7.20 (td, J = 7.6, 1.7 Hz, 1H), 5.00 (dd, J = 7.9, 4.2 Hz, 1H), 3.28 (s, 3H), 2.41 – 2.33 (m, 2H); **^{13}C NMR** (126 MHz, $CDCl_3$): δ (ppm) 139.3, 133.1, 129.7, 128.1, 127.3, 122.5, 119.1 (qt, $J_{C\text{-}F}$ = 285.5, 35.9 Hz), 114.5 (tq, $J_{C\text{-}F}$ = 253.2, 37.9 Hz), 75.7 (d, $J_{C\text{-}F}$ = 4.0 Hz),, 57.1, 37.9 (t, $J_{C\text{-}F}$ = 21.1 Hz); **^{19}F NMR** (471 MHz, $CDCl_3$): δ (ppm) -85.53 (s, 3F), -115.17 – 115.82 (m, 1F) , -116.91 – 117.55 (m, 1F); **HRMS** m/z (APCI): calcd. for $C_{10}H_7BrF_5$ [M-CH_3OH+H]$^+$: 300.9646; found: 300.9648.

(4,4,5,5,5-pentafluoro-2-methoxypentan-2-yl)benzene (5h)

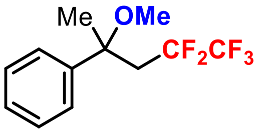

Following the general procedure (III). The product was purified by flash column chromatography on silica gel using hexane: EA= 40:1 as eluent and obtained as colorless oil (41.8 mg, 78% yield, R_f = 0.45 (hexane: EA= 20:1). **^{1}H NMR** (500 MHz, $CDCl_3$): δ (ppm) 7.44 – 7.37 (m, 4H), 7.33 – 7.29 (m, 1H), 4.54 (dd, J = 8.7, 3.6 Hz, 1H), 3.08 (s, 3H), 2.61 –2.39 (m, 2H), 1.80 (s, 3H); **^{13}C NMR** (126 MHz, $CDCl_3$): δ (ppm) 143.6, 128.6, 127.8, 126.2, 119.1 (qt, $J_{C\text{-}F}$ = 286.0, 35.9 Hz), 115.2 (tq, $J_{C\text{-}F}$ = 255.8, 37.1 Hz), 77.0, 50.2, 37.9 (t, $J_{C\text{-}F}$ = 19.5 Hz), 22.0 (t, $J_{C\text{-}F}$ = 2.7 Hz); **^{19}F NMR** (471 MHz, $CDCl_3$): δ (ppm) -86.50 (s, 3F), -114.52 (ddd, J = 265.9, 31.6, 7.5 Hz), -116.95 (ddd, J = 265.5, 31.1, 7.8 Hz); **HRMS** m/z (APCI): calcd. for $C_{11}H_{10}F_5$ [M-CH_3OH+H]$^+$: 237.0697; found: 237.0699.

(3,3,4,4,4-pentafluoro-1-methoxybutane-1,1-diyl)dibenzene (5i)

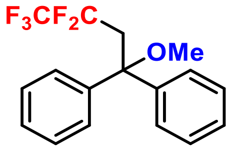

Following the general procedure (III). The product was purified by flash column chromatography on silica gel using hexane: EA= 40:1 as eluent and obtained as white solid (64.0 mg, 97% yield, R_f = 0.65 (hexane: EA= 20:1). **^{1}H NMR** (500 MHz, $CDCl_3$): δ (ppm) 7.38 – 7.31 (m, 8H), 7.28 – 7.24 (m, 2H), 3.22 – 3,15 (m, 5H); **^{13}C NMR** (126 MHz, $CDCl_3$): δ (ppm) 144.2, 128.3, 127.4, 126.6, 119.2 (qt, $J_{C\text{-}F}$ = 286.1, 35.9 Hz), 115.2 (tq, $J_{C\text{-}F}$ = 257.0, 36.8 Hz), 80.4, 51.1, 34.4 (t, $J_{C\text{-}F}$ = 19.0 Hz); **^{19}F NMR** (471 MHz, $CDCl_3$): δ (ppm) -86.75 (s, 3F), -117.01 (t, J = 17.7 Hz, 2F). **HRMS** m/z (ESI): calcd. for $C_{17}H_{15}F_5ONa$ [M+Na]$^+$: 353.0935; found: 353.0938.

(3,3,4,4,4-pentafluoro-1-methoxy-2-methylbutyl)benzene (5j)

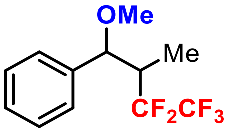

Following the general procedure (III). The product was purified by flash column chromatography on silica gel using hexane: EA= 40:1 as eluent and obtained as colorless oil (22.0 mg, 41% yield, dr = 3:1, R_f = 0.40 (hexane: EA= 20:1).^{1}**H NMR** (500 MHz, $CDCl_3$): δ (ppm) 7.40 – 7.36 (m, 3H), 7.30 – 7.28 (m, 2H), 4.69 (d, J = 2.2 Hz, 1H), 3.30 (s, 3H), 2.40 – 2.30 (m, 1H), 1.06 (d, J = 7.1 Hz, 3H); ^{13}**C NMR** (126 MHz, $CDCl_3$): δ (ppm) 139.5, 128.7, 127.9, 126.6, 123.4 – 114.0 (m, 2C), 79.2 (dd, J_{C-F} = 5.8, 2.5 Hz), 57.4, 43.5 (t, J_{C-F} = 19.8 Hz), 5.8 (tt, J_{C-F} = 4.1, 2.1 Hz); ^{19}**F NMR** (471 MHz, $CDCl_3$): δ (ppm) -82.30 (s, 3F), -117.17 (dd, J = 271.9, 10.7 Hz, 1F), -119.21 (dd, J = 272.0, 19.7 Hz, 1F); **HRMS** m/z (APCI): calcd. for $C_{11}H_{10}F_5$ $[M-CH_3OH+H]^+$: 237.0697; found: 237.0670.

(3,3,4,4,4-pentafluoro-1-methoxybutane-1,2-diyl)dibenzene (5k)

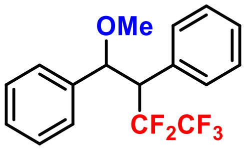

Following the general procedure (III). The product was purified by flash column chromatography on silica gel using hexane: EA= 40:1 as eluent and obtained as colorless oil (39.6 mg, 60% yield, dr = 3:1, R_f = 0.65 (hexane: EA= 20:1). ^{1}**H NMR** (500 MHz, $CDCl_3$): δ (ppm) 7.15 – 7.10 (m, 6H), 7.03 (dd, J = 6.7, 3.0 Hz, 2H), 6.99 (d, J = 8.2 Hz, 2H), 4.75 (d, J = 8.5 Hz, 1H), 3.78 – 3.70 (m, 1H), 3.23 (s, 3H); ^{13}**C NMR** (126 MHz, $CDCl_3$): δ (ppm) 138.3, 131.6, 130.4, 129.0, 128.172, 128.0, 127.8, 127.0, 123.1 – 113.9 (m, 2C), 82.8 (d, J_{C-F} = 4.0 Hz), 56.8, 54.7 (t, J_{C-F} = 19.8 Hz); ^{19}**F NMR** (471 MHz, $CDCl_3$): δ (ppm) -81.17 (s, 3F), -112.10 (dd, J = 273.5, 13.3 Hz, 1F), -116.49 (dd, J = 273.8, 19.9 Hz, 1F); **HRMS** m/z (APCI): calcd. for $C_{16}H_{12}F_5$ $[M-CH_3OH+H]^+$: 299.0854; found: 299.0855.

1-methoxy-2-(perfluoroethyl)-2,3-dihydro-1*H*-indene (5l)

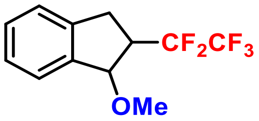

Following the general procedure (III). The product was purified by flash column chromatography on silica gel using hexane: EA= 40:1 as eluent and obtained as colorless oil (50.5 mg, 95% yield, dr = 3:1, R_f = 0.40 (hexane: EA= 20:1). ^{1}**H NMR** (500 MHz, $CDCl_3$): δ (ppm) 7.45 – 7.25 (m, 4H), 5.22 (d, J = 5.2 Hz, 1H), 3.56 (s, 3H), 3.32 – 3.24 (m, 1H), 3.17 – 3.07 (m, 2H); ^{13}**C NMR** (126 MHz, $CDCl_3$): δ (ppm) 140.8, 139.9, 129.2, 127.5, 125.0, 124.9, 120.9 – 113.4 (m, 2C), 84.1 (t, J_{C-F} = 3.4 Hz), 57.2 (d, J_{C-F} = 2.1 Hz), 47.0 (t, J_{C-F} = 21.0 Hz), 30.4 (ddd, J_{C-F} = 6.0, 3.9, 1.9 Hz); ^{19}**F NMR** (471 MHz, $CDCl_3$): δ (ppm) -83.21 (s, 3F), -118.00 (dd, J = 271.1, 11.0 Hz, 1F), -121.53 (dd, J = 271.0, 21.1 Hz, 1F); **HRMS** m/z (ESI): calcd. for $C_{12}H_{11}F_5ONa$ $[M+Na]^+$: 289.0622; found: 289.0622.

4-(1-ethoxy-3,3,4,4,4-pentafluorobutyl)-1,1'-biphenyl (6a)

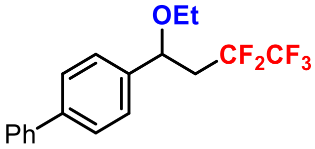

Following the general procedure (IV). The product was purified by flash column chromatography on silica gel using hexane: EA= 40:1 as eluent and obtained as colorless oil (50.2 mg, 73% yield, R_f = 0.45 (hexane: EA= 20:1). ^{1}**H NMR** (500 MHz, $CDCl_3$): δ (ppm) 7.63 – 7.60 (m, 4H), 7.46 (t, J = 7.5 Hz, 2H), 7.43 (d, J = 7.9 Hz, 2H), 7.38 (t, J = 7.3 Hz, 1H), 4.73 (dd, J = 8.9, 3.5 Hz, 1H), 3.49 – 3.38 (m, 2H), 2.72 – 2.60 (m, 1H), 2.39 – 2.29 (m, 1H), 1.22 (t, J = 7.0 Hz, 3H) ; ^{13}**C NMR** (126 MHz, $CDCl_3$): δ (ppm) 141.4, 140.8, 140.2, 129.0, 127.7, 127.6, 127.2, 126.9, 119.2 (qt, J_{C-F} = 285.5, 35.9 Hz), 114.9 (tq, J_{C-F} = 253.6, 37.9 Hz), 74.8 (t, J_{C-F} = 3.1 Hz), 64.6, 39.6 (t, J_{C-F} = 20.7 Hz), 15.2; ^{19}**F NMR** (471 MHz, $CDCl_3$): δ (ppm) -85.63 (s, 3F), -116.40 (ddd, J = 19.7, 15.5, 9.1 Hz, 2F); **HRMS** m/z (ESI): calcd. for $C_{18}H_{17}F_5ONa$ $[M+Na]^+$: 367.1092; found: 367.1094.

4-(3,3,4,4,4-pentafluoro-1-isopropoxybutyl)-1,1'-biphenyl (6b)

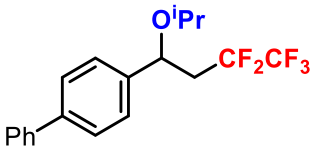

Following the general procedure (IV). The product was purified by flash column chromatography on silica gel using hexane: EA= 40:1 as eluent and obtained as colorless oil (40.1 mg, 56% yield, R_f = 0.45 (hexane: EA= 20:1). ^{1}**H NMR** (500 MHz, $CDCl_3$): δ (ppm) 7.61(d, J = 8.5 Hz, 4H), 7.45 (q, J = 7.8 Hz, 4H), 7.37 (t, J = 7.4 Hz, 1H), 4.87 (dd, J = 8.9, 3.4 Hz, 1H), 3.61 – 3.54 (m, 1H), 2.66 – 2.54 (m, 1H), 2.35 – 2.24 (m, 1H), 1.21 (d, J = 6.0 Hz, 3H), 1.11 (d, J = 6.2 Hz, 3H); ^{13}**C NMR** (126 MHz, $CDCl_3$): δ (ppm) 141.2, 141.1, 140.8, 129.0, 127.6, 127.2, 126.9, 119.2 (qt, J_{C-F} = 285.9, 36.0 Hz), 115.0 (tq, J_{C-F} = 254.5, 37.8 Hz), 72.1 (t, J_{C-F} = 3.5 Hz), 69.8, 39.6 (t, J_{C-F} = 20.5 Hz), 23.5, 21.0;^{19}**F NMR** (471 MHz, $CDCl_3$): δ (ppm) -85.78 (s, 3F), -116.63 (ddd, J = 267.0, 27.0, 10.3 Hz, 1F), -116.54 (ddd, J = 263.8, 25.9, 9.3 Hz, 1F); **HRMS** m/z (ESI): calcd. for $C_{19}H_{19}F_5ONa$ $[M+Na]^+$: 381.1248; found: 381.1255.

4-(1-(*tert*-butoxy)-3,3,4,4,4-pentafluorobutyl)-1,1'-biphenyl (6c)

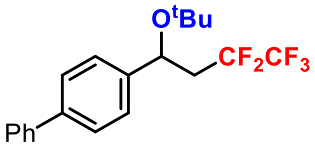

Following the general procedure (IV). The product was purified by flash column chromatography on silica gel using hexane: EA= 40:1 as eluent and obtained as colorless oil (35.0 mg, 47% yield, R_f = 0.45 (hexane: EA= 20:1). ^{1}**H NMR** (500 MHz, $CDCl_3$): δ (ppm) 7.59 (dd, J = 11.4, 7.7 Hz, 4H), 7.45 (dt, J = 7.8, 3.6 Hz, 4H), 7.36 (t, J = 7.4 Hz, 1H), 5.03 (dd, J = 8.9, 3.5 Hz, 1H), 2.60 – 2.48 (m, 1H), 2.28 – 2.17 (m, 1H), 1.17 (s, 9H); ^{13}**C NMR** (126 MHz, $CDCl_3$): δ (ppm) 144.1, 140.9, 140.6, 128.9, 127.5, 127.4, 127.2, 126.5, 119.2 (qt, J_{C-F} = 285.6, 36.0 Hz), 114.9 (m), 75.6, 67.9 (d, J_{C-F} = 3.2 Hz), 40.5 (t, J_{C-F} = 20.2 Hz), 28.8; ^{19}**F NMR** (471 MHz, $CDCl_3$): δ (ppm) -86.06 (s, 3F), -116.70 (ddd, J = 265.8, 29.4, 8.7 Hz, 1F), -117.98 (ddd, J = 265.4, 28.4, 7.1 Hz, 1F); **HRMS** m/z (ESI): calcd. for $C_{20}H_{21}F_5ONa$ $[M+Na]^+$: 395.1412; found: 395.1405.

4-(1-(benzyloxy)-3,3,4,4,4-pentafluorobutyl)-1,1'-biphenyl (6d)

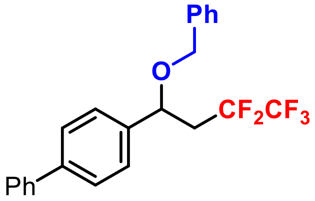

Following the general procedure (IV). The product was purified by flash column chromatography on silica gel using hexane: EA= 40:1 as eluent and obtained as colorless oil (42.2 mg, 52% yield, R_f = 0.40 (hexane: EA= 20:1). **^{1}H NMR** (500 MHz, $CDCl_3$): δ (ppm) 7.65 (dd, J = 11.6, 7.8 Hz, 4H), 7.49 (t, J = 8.4 Hz, 4H), 7.41 – 7.31 (m, 6H), 4.87 (dd, J = 8.8, 3.6 Hz, 1H), 4.51 (d, J = 11.4 Hz, 1H), 4.39 (d, J = 11.5 Hz, 1H), 2.82 – 2.70 (m, 1H), 2.45 – 2.34 (m, 1H); **^{13}C NMR** (126 MHz, $CDCl_3$): δ (ppm) 141.6, 140.7, 139.5, 137.7, 129.0, 128.5, 128.0, 127.9, 127.8, 127.7, 127.3, 127.2, 119.2 (qt, $J_{C\text{-}F}$ = 285.9, 36.0 Hz), 114.9 (tq, $J_{C\text{-}F}$ = 255.8, 37.8 Hz), 74.5 (d, $J_{C\text{-}F}$ = 3.8 Hz), 70.9, 39.3 (t, $J_{C\text{-}F}$ = 20.7 Hz); **^{19}F NMR** (471 MHz, $CDCl_3$): δ (ppm) -85.68 (s, 3F), -116.70 (ddd, J = 268.5, 27.0, 10.4 Hz, 1F), -117.98 (ddd, J = 267.1, 25.4, 9.1 Hz, 1F); **HRMS** m/z (ESI): calcd. for $C_{23}H_{19}F_5ONa$ $[M+Na]^+$: 429.1248; found: 429.1252.

4-(1-(cyclopentyloxy)-3,3,4,4,4-pentafluorobutyl)-1,1'-biphenyl (6e)

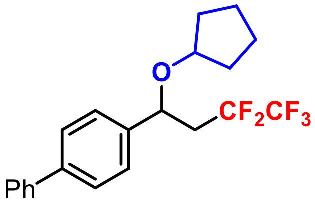

Following the general procedure (IV). The product was purified by flash column chromatography on silica gel using hexane: EA= 20:1 as eluent and obtained as colorless oil (42.2 mg, 55% yield, R_f = 0.40 (hexane: EA= 20:1). **^{1}H NMR** (500 MHz, $CDCl_3$): δ (ppm) 7.62 – 7.60 (m,4H), 7.48 – 7.42 (m,4H), 7.37 (t, J = 7.3 Hz, 1H), 7.36 (t, J = 7.4 Hz, 1H), 4.81 (dd, J = 9.1, 3.2 Hz, 1H), 3.89 – 3.85 (m, 1H), 2.65 – 2.53 (m, 1H), 2.34 – 2.23 (m, 1H), 1.83 – 1.70 (m, 3H), 1.66 – 1.61 (m, 3H), 1.56 – 1.43 (m, 2H); **^{13}C NMR** (126 MHz, $CDCl_3$): δ (ppm) 141.2, 140.8, 129.0, 127.59, 127.57, 127.2, 127.0, 119.2 (qt, $J_{C\text{-}F}$ = 285.6, 35.9 Hz), 114.9 (tq, $J_{C\text{-}F}$ = 253.3, 37.7 Hz), 79.4, 72.6 (t, $J_{C\text{-}F}$ = 3.6 Hz), 39.52 (t, $J_{C\text{-}F}$ = 20.5 Hz), 33.3, 31.2, 23.5, 23.4; **^{19}F NMR** (471 MHz, $CDCl_3$): δ (ppm) -85.83 (s, 3F), -116.18 (ddd, J = 266.9, 28.2, 9.3 Hz, 1F), -117.61 (ddd, J = 266.5, 26.6, 8.3 Hz, 1F); **HRMS** m/z (ESI): calcd. for $C_{21}H_{22}F_5O$ $[M+H]^+$: 385.1585; found: 385.1585.

4-(3,3,4,4,4-pentafluoro-1-phenoxybutyl)-1,1'-biphenyl (6f)

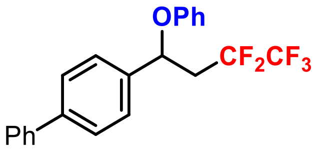

Following the general procedure (IV). The product was purified by flash column chromatography on silica gel using hexane: EA= 40:1 as eluent and obtained as colorless oil (25.1 mg, 32% yield, R_f = 0.35 (hexane: EA= 20:1). **^{1}H NMR** (500 MHz, $CDCl_3$): δ (ppm) 7.59 (dd, J = 14.8, 7.8 Hz, 4H), 7.48 (d, J = 8.0 Hz, 2H), 7.44 (t, J = 7.6 Hz, 2H), 7.36 (t, J = 7.4 Hz, 1H), 7.22 (t, J = 7.8 Hz, 2H), 6.94 (t, J = 7.4 Hz, 1H), 6.89 (d, J = 8.1 Hz, 2H), 5.59 (dd, J = 9.0, 3.1 Hz, 1H), 2.97 – 2.85 (m, 1H), 2.57 – 2.46 (m, 1H); **^{13}C NMR** (126 MHz, $CDCl_3$): δ (ppm) 157.4, 141.5, 140.6, 139.3, 129.6, 129.0, 127.9, 127.7, 127.2, 126.4, 121.7, 119.1 (qt, $J_{C\text{-}F}$ = 285.7, 35.8 Hz), 116.2, 114.7 (tq, $J_{C\text{-}F}$ = 254.5, 38.1 Hz),73.5 (d, $J_{C\text{-}F}$ = 3.1 Hz), 39.9 (t, $J_{C\text{-}F}$ = 20.9 Hz); **^{19}F NMR** (471 MHz, $CDCl_3$): δ

(ppm) -85.55 (s, 3F), -116.55 (ddd, J = 20.6, 15.6, 5.4 Hz, 2F); **HRMS** m/z (ESI): calcd. for $C_{22}H_{17}F_5ONa$ [M+Na] $^+$:415.1092; found: 415.1095.

1-([1,1'-biphenyl]-4-yl)-3,3,4,4,4-pentafluorobutyl acetate (6g)

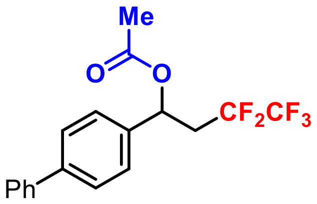

Following the general procedure (IV). The product was purified by flash column chromatography on silica gel using hexane: EA= 40:1 as eluent and obtained as white solid (31.5 mg, 30% yield, R_f = 0.35 (hexane: EA= 20:1). **^{1}H NMR** (500 MHz, $CDCl_3$): δ (ppm) 7.58 (dd, J = 15.2, 7.7 Hz, 4H), 7.45 (t, J = 7.4 Hz, 4H), 7.36 (t, J = 7.3 Hz, 1H), 6.24 (dd, J = 9.4, 3.4 Hz, 1H), 2.87 – 2.75 (m, 1H), 2.53 – 2.43 (m, 1H), 2.10 (s, 3H); **^{13}C NMR** (126 MHz, $CDCl_3$): δ (ppm) 169.7, 141.9, 140.5, 138.0, 129.0, 127.8, 127.7, 127.3, 126.9, 119.0 (qt, $J_{C\text{-}F}$ = 285.4, 35.5 Hz), 114.5 (tq, $J_{C\text{-}F}$ = 254.5, 37.8 Hz), 68.5 (t, $J_{C\text{-}F}$ = 3.1 Hz), 37.4 (t, $J_{C\text{-}F}$ = 21.0 Hz), 21.1;**^{19}F NMR** (471 MHz, $CDCl_3$): δ (ppm) -85.72 (s, 3F), -116.77 (ddd, J = 268.5, 27.3, 10.0 Hz, 1F), -117.70 (ddd, J = 268.6, 25.2, 10.5 Hz, 1F); **HRMS** m/z (ESI): calcd. for $C_{18}H_{15}F_5O_2Na$ [M+Na] $^+$: 381.0884; found: 381.0884.

(1-([1,1'-biphenyl]-4-yl)-3,3,4,4,4-pentafluorobutyl)(benzyl)sulfane (6h)

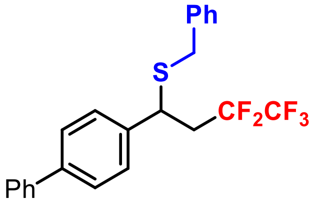

Following the general procedure (IV). The product was purified by flash column chromatography on silica gel using hexane as eluent and obtained as white solid (30.4 mg, 36% yield, R_f = 0.35 (100% hexane). **^{1}H NMR** (500 MHz, $CDCl_3$): δ (ppm) 7.63 (d, J = 7.1 Hz, 2H), 7.60 (d, J = 8.1 Hz, 2H), 7.47 (t, J = 7.6 Hz, 2H), 7.40 – 7.37 (m, 3H), 7.34 (t, J = 7.2 Hz, 2H), 7.29 – 7.26 (m, 3H), 4.11 (dd, J = 8.9, 5.1 Hz, 1H), 3.63 – 3.53 (m, 2H), 2.76 – 2.57 (m, 2H); **^{13}C NMR** (126 MHz, $CDCl_3$): δ (ppm) 140.8, 140.6, 139.7, 137.4, 129.1, 129.0, 128.7, 128.3, 127.6, 127.5, 127.4, 127.2, 118.9 (qt, $J_{C\text{-}F}$ = 285.7, 35.7 Hz), 114.8 (tq, $J_{C\text{-}F}$ = 254.5, 37.8 Hz), 41.0, 37.2 (t, $J_{C\text{-}F}$ = 20.7 Hz), 36.1;150.0, 142.2, 140.4, 134.4, 130.0, 129.1, 129.0, 128.9, 128.1, 127.8, 127.7, 127.3, 127.1, 121.4 (t, $J_{C\text{-}F}$ = 31.1 Hz), 120.1 (qt, $J_{C\text{-}F}$ = 288.9, 38.7 Hz), 111.1 (tq, $J_{C\text{-}F}$ = 257.0, 41.3 Hz), 54.9. **^{19}F NMR** (471 MHz, $CDCl_3$): δ (ppm) -85.75 (s, 3F), -116.04 (ddd, J = 265.3, 26.5, 9.3 Hz, 1F), -117.12 (ddd, J = 265.4, 26.4, 9.9 Hz, 1F); **HRMS** m/z (ESI): calcd. for $C_{23}H_{18}F_5S$ [M-H] $^-$: 421.1055; found: 421.1056.

***N*-(1-([1,1'-biphenyl]-4-yl)-3,3,4,4,4-pentafluorobutyl)aniline (6i)**

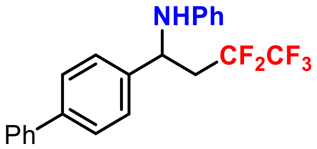

Following the general procedure (IV). The product was purified by flash column chromatography on silica gel using hexane: EA= 20:1 as eluent and obtained as colorless oil (32.8 mg, 42% yield, R_f = 0.45 (hexane: EA= 10:1). **^{1}H NMR** (500 MHz, $CDCl_3$): δ (ppm) 7.62 (t, J = 7.6 Hz, 4H), 7.51 – 7.46 (m, 4H), 7.39 (t, J = 7.4 Hz, 1H), 7.20 – 7.17 (m, 2H), 6.77 (t, J = 7.3 Hz, 1H), 6.63 (d, J = 8.0 Hz, 2H), 4.93 (dd, J = 8.0, 5.2 Hz, 1H), 4.26

(s, 1H), 2.71 – 2.53 (m, 2H); **13C NMR** (126 MHz, $CDCl_3$): δ (ppm) 146.3, 141.2, 141.0, 140.6, 129.4, 128.9, 128.0, 127.6, 127.2, 126.7, 119.3 (qt, J_{C-F} = 287.3, 35.7 Hz), 118.5, 115.9 (tq, J_{C-F} = 254.5, 37.9 Hz), 113.8, 52.1, 38.6 (t, J_{C-F} = 20.4 Hz); **19F NMR** (471 MHz, $CDCl_3$): δ (ppm) -85.66 (s, 3F), -115.47 (ddd, J = 268.5, 25.6, 12.1Hz, 1F), -117.55 (ddd, J = 268.5, 23.5, 12.0 Hz, 1F); **HRMS** m/z (ESI): calcd. for $C_{22}H_{18}F_5ClN$ $[M+Cl]^+$: 426.1053; found: 426.1059.

1-([1,1'-biphenyl]-4-yl)-3,3,4,4,4-pentafluorobutan-1-ol (6j)

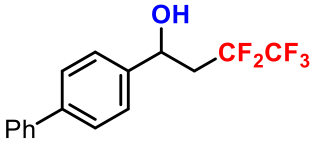

Following the general procedure (IV). The solvent was H_2O/MeCN = 1:9 (0.1 M), H_2O in the solvent was the source of nucleophile substrate. The product was purified by flash column chromatography on silica gel using hexane: EA= 20:1 as eluent and obtained as white solid (51.8 mg, 82% yield, R_f = 0.40 (hexane: EA= 10:1). **1H NMR** (500 MHz, $CDCl_3$): δ (ppm) 7.63 – 7.58 (m, 4H), 7.48 – 7.44 (m, 4H), 7.40 – 7.35 (m, 1H), 5.26 (d, J = 9.0 Hz, 1H), 2.71 – 2.56 (m, 1H), 2.50 – 2.36 (m, 1H), 2.27 (s, 1H); **13C NMR** (126 MHz, $CDCl_3$): δ (ppm) 141.7, 141.5, 140.6, 129.0, 127.73, 127.68, 127.2, 126.2, 119.1 (qt, J_{C-F} = 285.5, 35.8 Hz), 115.3 (tq, J_{C-F} = 254.1, 38.1 Hz), 67.86 (t, J_{C-F} = 2.9 Hz), 39.85 (t, J_{C-F} = 20.4 Hz); **19F NMR** (471 MHz, $CDCl_3$): δ (ppm) -85.77 (s, 3F), -116.40 (ddd, J = 267.0, 27.9, 9.2Hz, 1F), -117.61 (ddd, J = 267.1, 26.7, 9.6 Hz, 1F); **HRMS** m/z (ESI): calcd. for $C_{16}H_{13}F_5ClO$ $[M+Cl]^+$: 351.0581; found: 351.0583.

4-(3,3,4,4,4-pentafluoro-1-(2,4,6-trimethoxyphenyl)butyl)-1,1'-biphenyl (6k)

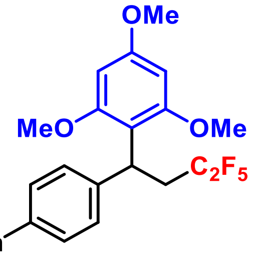

Following the general procedure (IV). The product was purified by flash column chromatography on silica gel using hexane: EA= 20:1 as eluent and obtained as white solid (77.4 mg, 83% yield, R_f = 0.35 (hexane: EA= 10:1). **1H NMR** (500 MHz, $CDCl_3$): δ (ppm) 7.59 (dd, J = 8.3, 1,3 Hz, 2H), 7.52 – 7.50 (m, 2H), 7.46 – 7.42 (m, 4H), 7.35 – 7.32 (m, 1H), 6.17 (s,2H), 5.19 (dd, J = 8.5, 5.6 Hz, 1H), 3.83 (d, J = 13.8 Hz, 9H), 3.29 – 3.16 (m, 1H), 3.02 – 2.90 (m, 1H); **13C NMR** (126 MHz, $CDCl_3$): δ (ppm) 160.2, 158.8, 143.0, 141.2, 138.9, 128.8, 128.1, 127.1, 126.9, 119.4 (qt, J_{C-F} = 285.6, 36.3 Hz), 116.3 (tq, J_{C-F} = 253.3, 37.0 Hz), 112.1, 91.4, 55.9, 55.3, 33.2 (t, J_{C-F} = 20.2 Hz), 31.8 (t, J_{C-F} = 2.7 Hz); **19F NMR** (471 MHz, $CDCl_3$): δ (ppm) -85.82 (s, 3F), -118.40 (t, J = 18.8 Hz, 2F); **HRMS** m/z (ESI): calcd. for $C_{25}H_{24}F_5O_3$ $[M+H]^+$: 467.1640; found: 467.1643.

1,3,5-trimethoxy-2-(3,3,4,4,4-pentafluoro-1-(4-methoxyphenyl)butyl)benzene (6l)

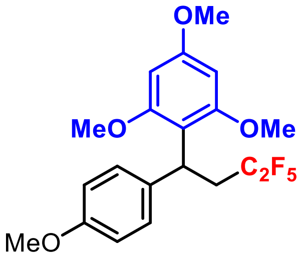

Following the general procedure (IV). The product was purified by flash column chromatography on silica gel using hexane: EA= 10:1 as eluent and obtained as white solid (68.9 mg, 82% yield, R_f = 0.25 (hexane: EA= 10:1). **^{1}H NMR** (500 MHz, $CDCl_3$): δ (ppm) 7.29 (d, J = 8.7 Hz, 2H), 6.79 (d, J = 8.7 Hz, 2H), 6.13 (s, 2H), 5.06 (dd, J = 8.3, 5.8 Hz, 1H), 3.80 (s, 6H), 3.79 (s, 3H), 3.76 (s, 3H), 3.19 – 3.07 (m, 1H), 2.92 – 2.80 (m, 1H); **^{13}C NMR** (126 MHz, $CDCl_3$): δ (ppm) 160.0, 158.7, 157.8, 136.1, 128.7, 122.8 – 114.1 (m, 2C), 113.5, 112.5, 91.3, 55.8, 55.33, 55.28, 33.4 (t, $J_{C\text{-}F}$ = 20.1 Hz), 31.4; **^{19}F NMR** (471 MHz, $CDCl_3$): δ (ppm) -85.89 (s, 3F), -118.48 – -118.56 (m, 2F); **HRMS** m/z (ESI): calcd. for $C_{20}H_{22}F_5O_4$ $[M+H]^+$: 421.1433; found: 421.1436.

1,3,5-trimethoxy-2-(perfluoroethyl)benzene (7a), known compound, CAS No.: 1535199-31-5.

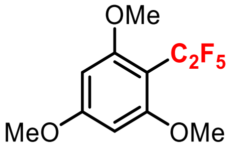

Following the general procedure (V). The product was purified by flash column chromatography on silica gel using hexane as eluent and obtained as colorless oil (48.6 mg, 85% yield, R_f = 0.30 (100% hexane). **^{1}H NMR** (500 MHz, $CDCl_3$): δ (ppm) 6.14 (s, 2H), 3.83 (s, 3H), 3.81 (s, 6H); **^{13}C NMR** (126 MHz, $CDCl_3$): δ (ppm) 163.9, 161.7 (t, $J_{C\text{-}F}$ = 2.2 Hz), 120.0 (qt, $J_{C\text{-}F}$ = 287.5 Hz, 39.8 Hz), 114.4 (tq, $J_{C\text{-}F}$ = 258.3 Hz, 40.4 Hz), 98.3 (t, J = 21.8 Hz), 91.8, 56.4, 55.5; **^{19}F NMR** (471 MHz, $CDCl_3$): δ (ppm) -85.06 (t, J = 3.1 Hz, 3F), -106.70 (q, J = 3.1 Hz, 2F); The physical and spectral data were consistent with previously reported.[6]

1,4-dimethoxy-2-(perfluoroethyl)benzene (7b), known compound, CAS No.:1322085-76-6.

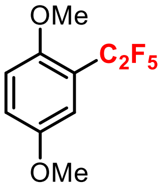

Following the general procedure (V). The product was purified by flash column chromatography on silica gel using hexane as eluent and obtained as colorless oil (31.8 mg, 60% yield, R_f = 0.40 (100% hexane). **^{1}H NMR** (500 MHz, $CDCl_3$): δ (ppm) 7.05 – 7.03 (m, 2H), 6.97 – 6.95 (m, 1H), 3.82 (s, 1H) , 3.79 (s, 1H); **^{13}C NMR** (126 MHz, $CDCl_3$): δ (ppm) 153.4, 152.5 (t, $J_{C\text{-}F}$ = 2.7 Hz), 119.5 (qt, $J_{C\text{-}F}$ = 287.3 Hz, 39.1 Hz), 118.6, 117.6 (t, $J_{C\text{-}F}$ = 22.6 Hz), 114.3 (d, $J_{C\text{-}F}$ = 17.8 Hz), 114.2, 113.6 (tq, $J_{C\text{-}F}$ = 255.8 Hz, 39.7 Hz), 56.8, 56.0; **^{19}F NMR** (471 MHz, $CDCl_3$): δ (ppm) -83.84 (s, 3F), -111.86 (d, J = 2.5 Hz, 2F); The physical and spectral data were consistent with previously reported.[7]

5-(perfluoroethyl)-2,3-dihydrothieno[3,4-b][1,4]dioxine (7c)

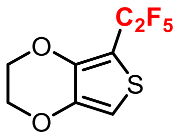

Following the general procedure (V). The product was purified by flash column chromatography on silica gel (EA: hexane = 1: 20) and obtained as colorless oil (37.4 mg, 72% yield, R_f = 0.45 (EA: hexane = 1: 10). ^{1}**H NMR** (500 MHz, $CDCl_3$): δ (ppm) 6.57 (s, 1H), 4.28 (td, J = 3.7, 2.1 Hz, 2H), 4.23 (td, J = 3.7, 2.1 Hz, 2H),; ^{13}**C NMR** (126 MHz, $CDCl_3$): δ (ppm) 143.1 (t, $J_{C\text{-}F}$ = 4.6 Hz), 141.6, 119.2 (qt, $J_{C\text{-}F}$ = 286.7, 40.5 Hz), 112.3 (tq, $J_{C\text{-}F}$ = 253.7, 40.8 Hz), 103.6 (t, $J_{C\text{-}F}$ = 2.5 Hz), 102.5 (t, $J_{C\text{-}F}$ = 29.8 Hz), 65.0, 64.3; ^{19}**F NMR** (471 MHz, $CDCl_3$): δ (ppm) -84.66 (t, J = 3.0 Hz, 3F), -106.96 (1, J = 3.4 Hz, 2F); **HRMS** m/z (ESI): $[M\text{-}H]^-$ calcd. for $C_8H_4F_5O_2S$ 258.9858; Found 258.9860.

4-(3,3,3-trifluoro-1-methoxypropyl)-1,1'-biphenyl

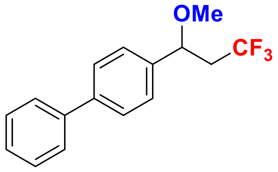

Following the general procedure (III). The product was purified by preparative TLC (petroleum ether: CH_2Cl_2 = 75: 25). ^{1}**H NMR (300 MHz, $CDCl_3$)** δ: 7.68-7.58 (m, 4H), 7.52-7.33 (m, 5H), 4.53 (dd, J = 8.6 Hz, J = 4.2 Hz, 1H), 3.29 (s, 3H), 2.82-2.61 (m, 1H), 2.53-2.33 (m, 1H). ^{19}**F NMR (188 MHz, $CDCl_3$) δ:** – 64.2 (t, J = 10.5 Hz, 3F). ^{13}**C NMR (75 MHz, $CDCl_3$) δ:** 141.5, 140.7, 139.1, 129.0 (2C), 127.7 (2C), 127.6, 127.2 (2C), 127.1 (2C), 125.9 (q, J = 278 Hz), 77.7 (q, J = 3.4 Hz), 56.9, 42.4 (q, J = 28 Hz). The physical and spectral data were consistent with previously reported.[8]

(4,4,4-trifluoro-2-methoxybutan-2-yl)benzene

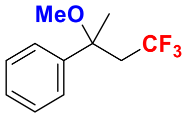

Following the general procedure (III). The product was purified by preparative TLC (petroleum ether: CH_2Cl_2 = 66: 33). ^{1}**H NMR (300 MHz, $CDCl_3$)** δ: 7.44-7.27 (m, 4H), 3.08 (s, 3H), 2.76-2.42 (m, 2H), 1.74 (s, 3H). ^{19}**F NMR (282 MHz, $CDCl_3$) δ:** – 60.6 (td, J = 10.9, 1.0 Hz, 3F). ^{13}**C NMR (75 MHz, $CDCl_3$) δ:** 143.3, 128.6 (2C), 127.8, 126.2 (2C), 125.6 (q, J = 278 Hz), 76.3 (q, J = 2.2 Hz), 50.3, 46.6 (q, J = 26.4 Hz), 22.0 (q, J = 1.8 Hz). The physical and spectral data were consistent with previously reported.[9]

(3,3,3-trifluoro-1-methoxypropane-1,2-diyl)dibenzene

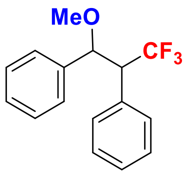

Following the general procedure (III). The product was purified by preparative TLC (petroleum ether: CH_2Cl_2 = 66: 33). The major isomer is described (syn): **^{1}H NMR (300 MHz, CDCl$_3$)** δ: 7.21-7.11 (m, 6H), 7.06-6.97 (m, 4H), 4.62 (d, J = 9.4 Hz, 1H), 3.74-3.59 (m, 1H), 3.26 (s, 3H). **^{19}F NMR (282 MHz, CDCl$_3$) δ:** – 63.8 (d, J = 8.9 Hz, 3F). **^{13}C NMR (75 MHz, CDCl$_3$) δ:** 138.3, 133.1 (q, J = 2.2 Hz), 129.49, 129.47, 128.4 (2C), 128.2 (2C), 128.1, 128.0, 127.8 (2C), 126.5 (q, J = 282 Hz), 83.7 (q, J = 1.7 Hz), 57.1 (q, J = 25.5 Hz), 57.0. The physical and spectral data were consistent with previously reported.[10]

4-(3,3,3-trifluoro-1-(2,4,6-trimethoxyphenyl)propyl)-1,1'-biphenyl

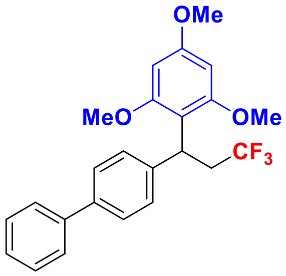

Following the general procedure (IV). The product was purified by preparative TLC (petroleum ether: CH_2Cl_2 = 60: 40). **^{1}H NMR (300 MHz, CDCl$_3$)** δ: 7.62-7.54 (m, 2H), 7.53-7.47 (m, 2H), 7.46-7.37 (m, 4H), 7.36-7.28 (m, 1H), 6.16 (s, 2H), 5.06 (dd, J = 6.1, 8.7 Hz, 1H), 3.82 (s, 6H), 3.81 (s, 3H), 3.34-2.90 (m, 2H). **^{19}F NMR (188 MHz, CDCl$_3$) δ:** – 65.22 (t, J = 10.8 Hz, 3F). **^{13}C NMR (75 MHz, CDCl$_3$) δ:** 160.2, 158.9, 142.8, 141.2, 138.8, 128.8, 128.1, 127.5 (q, J = 277 Hz), 127.1, 126.8, 111.8, 91.4, 55.9, 55.4, 36.7 (q, J = 26.9 Hz), 33.2 (q, J = 2.9 Hz). **HRMS** m/z (ESI): calcd. for $C_{24}H_{24}F_3O_3$ $[M+H]^+$: 417.1667; found: 417.1672.

1,3,5-trimethoxy-2-(trifluoromethyl)benzene

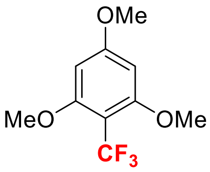

Following the general procedure (V). The product was purified by preparative TLC (petroleum ether: CH_2Cl_2 = 60: 40). **^{1}H NMR (300 MHz, CDCl$_3$)** δ: 6.13 (s, 2H), 3.23 (s, 9H). **^{19}F NMR (188 MHz, CDCl$_3$) δ:** – 54.6 (s, 3F). **^{13}C NMR (75 MHz, CDCl$_3$) δ:** 163.7, 160.6, 124.5 (q, J = 273 Hz), 100.5 (q, J = 30.3 Hz), 99.9, 56.3, 55.5. The physical and spectral data were consistent with previously reported. [11]

X. Reference:

1. Zheng, Y.; Qing, F. L.; Huang, Y.; Xu, X. H.; Tunable and Practical Synthesis of Thiosulfonates and Disulfides from Sulfonyl Chlorides in the Presence of Tetrabutylammonium Iodide. *Adv. Synth. Catal.* **2016,** *358,* 3477–3481.

2. Luo, Z.; Yang, X.; Tsui, G. C.; Perfluoroalkylation of Thiosulfonates: Synthesis of Perfluoroalkyl Sulfides. *Org. Lett.* **2020,** *22,* 6155–6159.

3. Chaabouni, S.; Lohier, J.-F.; Barthelemy, A.-L.; Glachet, T.; Anselmi, E.; Dagousset, G.; Diter, P.; Pegot, B.; Magnier, E.; Reboul, V. One-Pot Synthesis of Aryl- and Alkyl S-Perfluoroalkylated *NH*-Sulfoximines from Sulfides. *Chem. Eur. J.* **2018,** *24,*17006–17010.

4. Katayev, D.; Vaclavik, J.; Bruning, F.; Commare, B.; Togni, A. Synthesis of quaternary α-perfluoroalkyl lactams via electrophilic perfluoroalkylation. *Chem. Commun.* **2016,** *52,* 4049-4052.

5. Bellotti, P.; Huang, H.-M.; Faber, T.; Laskar, R.; Glorius, F.; Catalytic defluorinative ketyl–olefin coupling by halogen-atom transfer. *Chem. Sci.* **2022,** *13,* 7855-7862.

6. Deolka, S.; Govindarajan, R.; Vasylevskyi, S.; Roy, M. C.; Khusnutdinova, J. R.; E. Khaskin, Ligand-free nickel catalyzed perfluoroalkylation of arenes and heteroarenes. *Chem. Sci.* **2022,** *13,* 12971–12979.

7. Tahara, R.; Fukuhara, T.; Hara, S.; A novel method for introducing a polyfluoroalkyl group into aromatic compounds. *J. Fluorine. Chem.* **2011,** *132,* 579–586.

8. Ge, H.; Wu, B.; Liu, Y.; Wang, H.; Shen, Q.; Synergistic Lewis Acid and Photoredox-Catalyzed Trifluoromethylative Difunctionalization of Alkenes with Selenium Ylide-Based Trifluoromethylating Reagent. A*CS Catal.* **2020,** *10,* 12414–12424.

9. Zhang, L.; Zhang, G.; Wang, P.; Li, Y.; Lei, A.; Electrochemical Oxidation with Lewis-Acid Catalysis Leads to Trifluoromethylative Difunctionalization of Alkenes Using CF_3SO_2Na. *Org. Lett.* **2018,** *20,* 7396−7399.

10. Yasu, Y.; Koike, T.; Akita, M.; Three-component Oxytrifluoromethylation of Alkenes: Highly Efficient and Regioselective Difunctionalization of C=C Bonds Mediated by Photoredox Catalysts. *Angew. Chem. Int. Ed.* **2012,** *51,* 9567 –9571.

11. Li, L.; Mu, X.; Liu, W.; Wang, Y.; Mi, Z.; Li, C.; Simple and Clean Photoinduced Aromatic Trifluoromethylation Reaction. *J. Am. Chem. Soc.* **2016,** *138,* 5809−5812.

XI. Spectra.

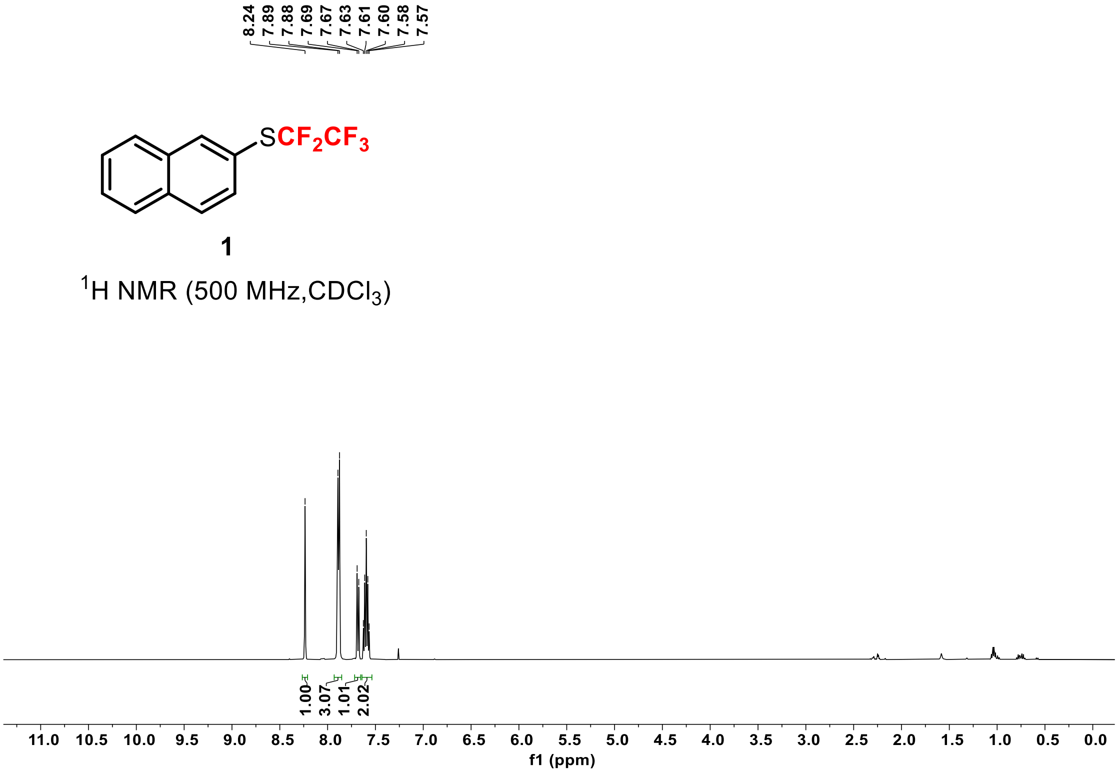
8.24
7.89
7.88
7.69
7.67
7.63
7.61
7.60
7.58
7.57
SCF2CF3
1
1H NMR (500 MHz,CDCl3)
1.00
3.07
1.01
2.02
11.0 10.5 10.0 9.5 9.0 8.5 8.0 7.5 7.0 6.5 6.0 5.5 5.0 4.5 4.0 3.5 3.0 2.5 2.0 1.5 1.0 0.5 0.0
f1 (ppm)

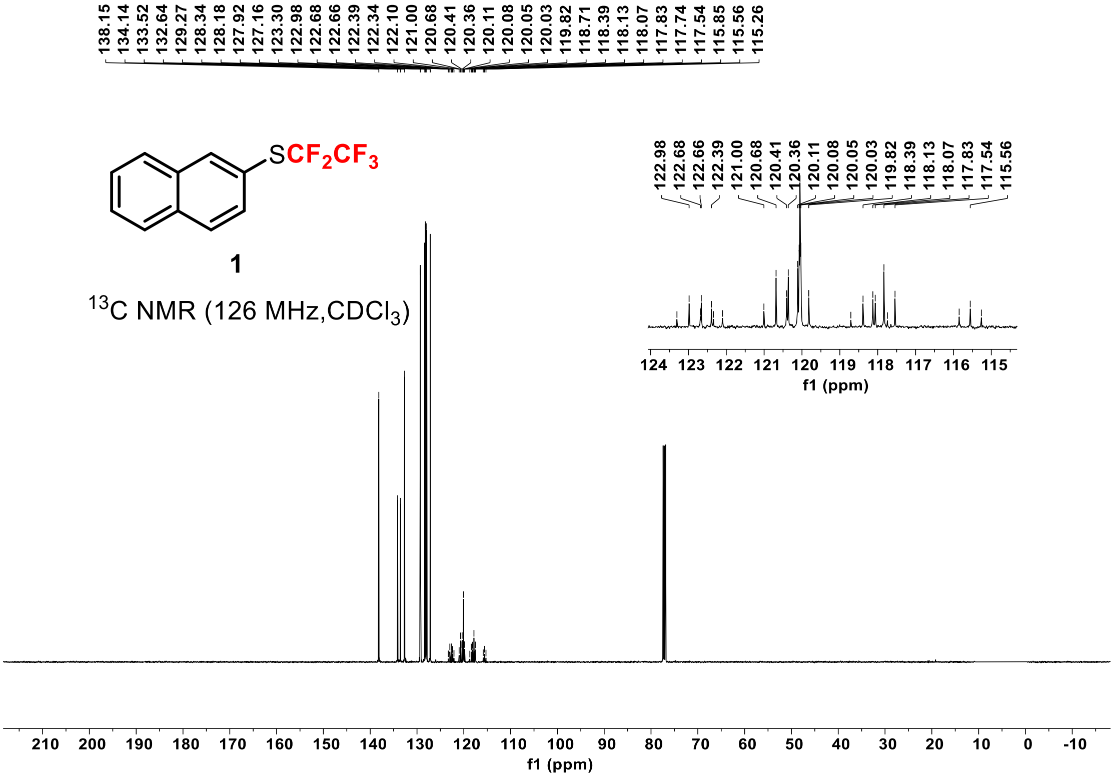
138.15
134.14
133.52
132.64
129.27
128.34
128.18
127.92
127.16
123.30
122.98
122.68
122.66
122.39
122.34
122.10
121.00
120.68
120.41
120.36
120.11
120.08
120.05
120.03
119.82
118.71
118.39
118.13
118.07
117.83
117.74
117.54
115.85
115.56
115.26
SCF2CF3
1
13C NMR (126 MHz,CDCl3)
122.98
122.68
122.66
122.39
121.00
120.68
120.41
120.36
120.11
120.08
120.05
120.03
119.82
118.39
118.13
118.07
117.83
117.54
115.56
124 123 122 121 120 119 118 117 116 115
f1 (ppm)
210 200 190 180 170 160 150 140 130 120 110 100 90 80 70 60 50 40 30 20 10 0 -10
f1 (ppm)

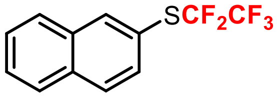

1

^{19}F NMR (471 MHz,CDCl$_3$)

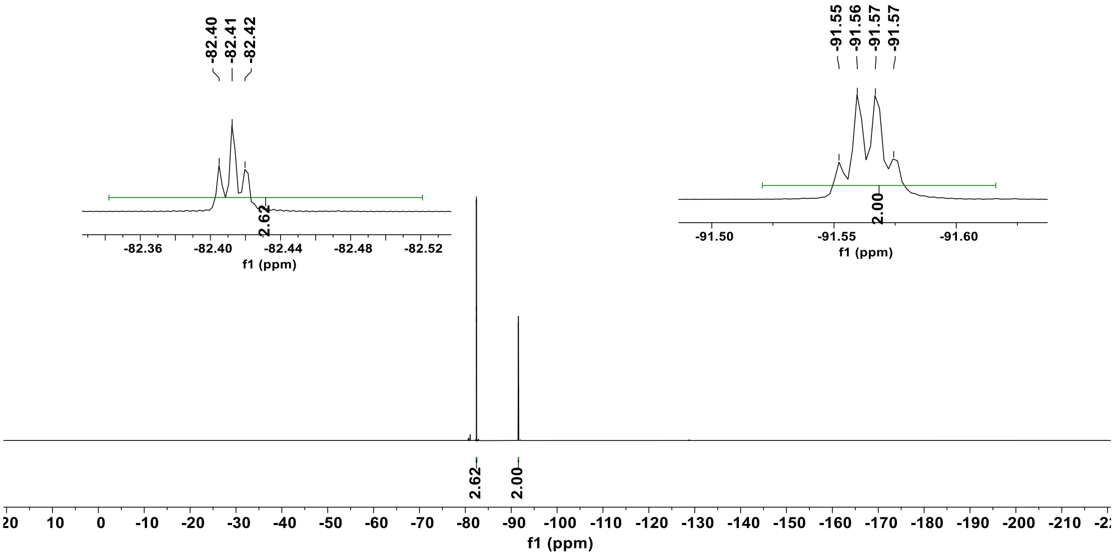

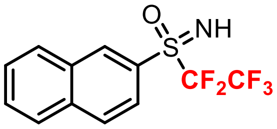

2

^{1}H NMR (500 MHz, CDCl$_3$)

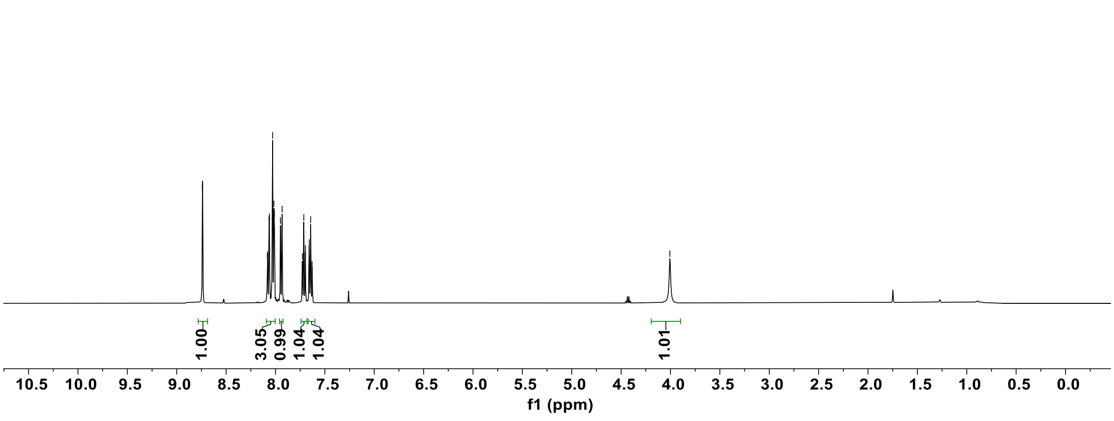

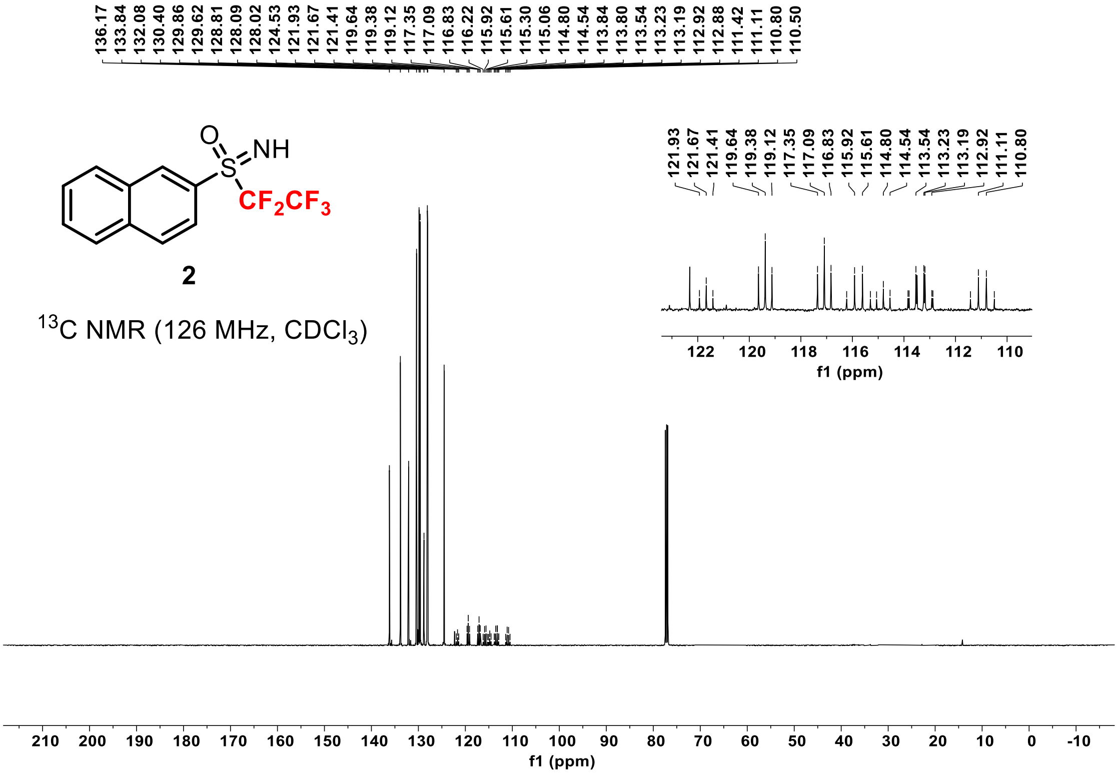
2
^{13}C NMR (126 MHz, CDCl$_3$)
f1 (ppm)

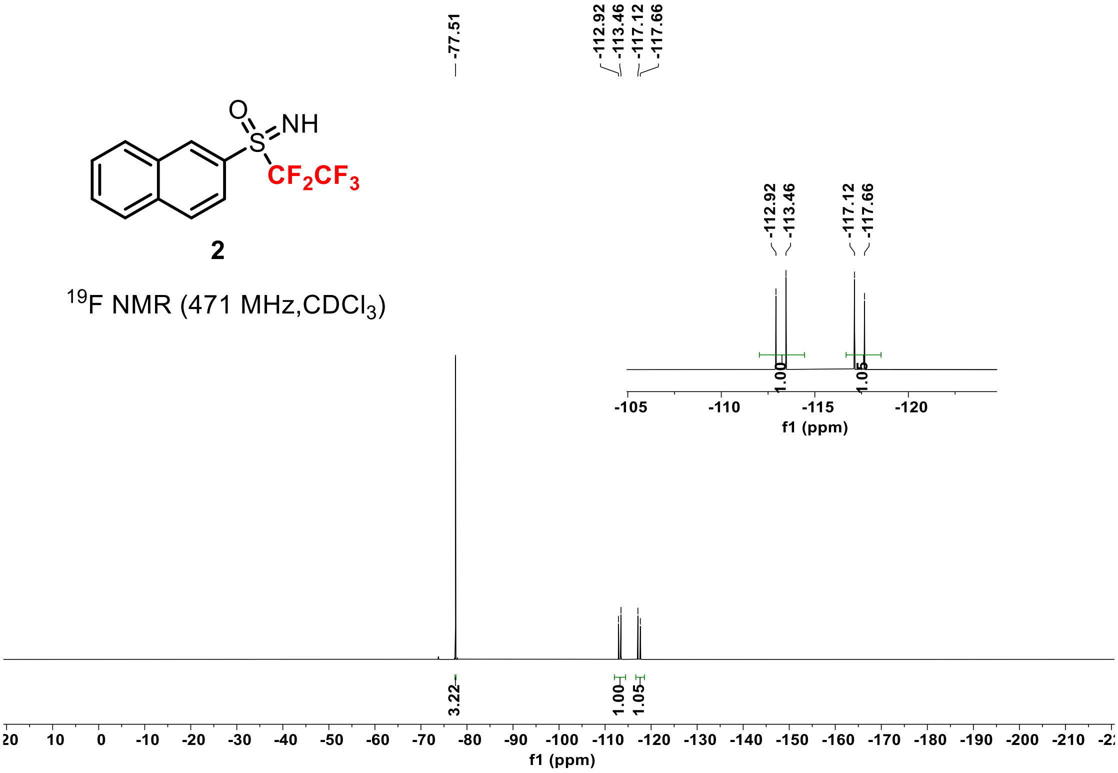
2
^{19}F NMR (471 MHz,CDCl$_3$)
f1 (ppm)

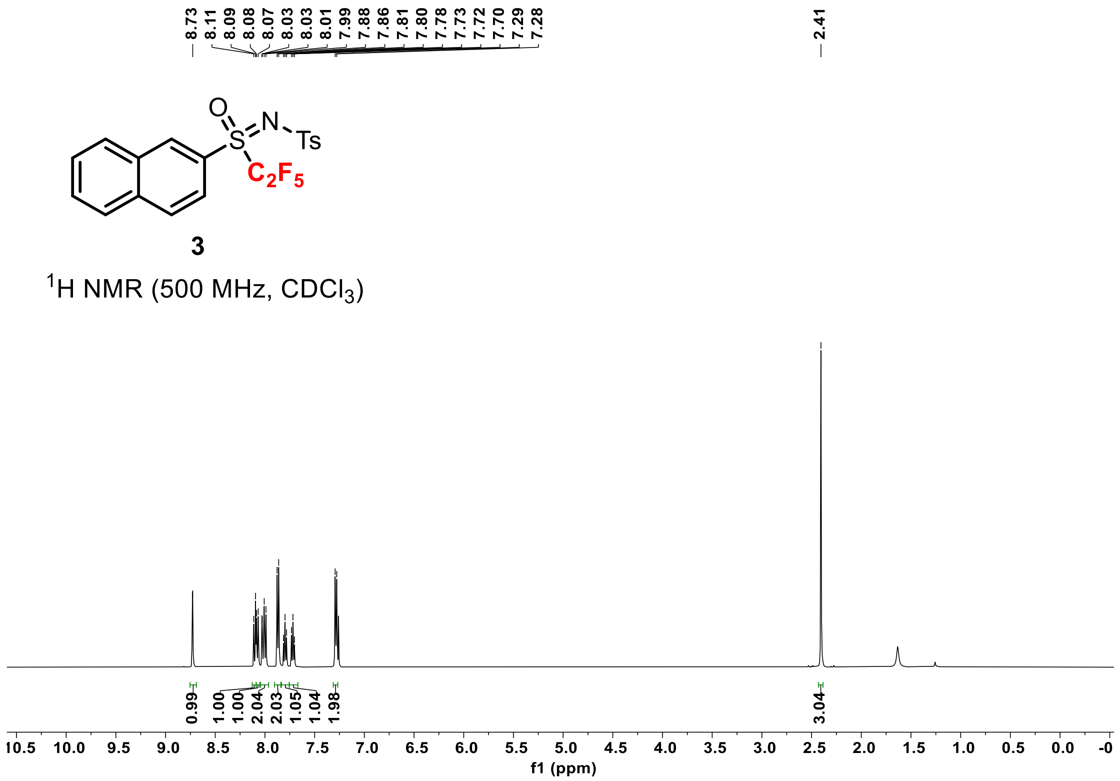
8.73
8.11
8.09
8.08
8.07
8.03
8.03
8.01
7.99
7.88
7.86
7.81
7.80
7.78
7.73
7.72
7.70
7.29
7.28
2.41
O
N
Ts
S
C2F5
3
1H NMR (500 MHz, CDCl3)
0.99
1.00
1.00
2.04
2.03
1.05
1.04
1.98
3.04
f1 (ppm)

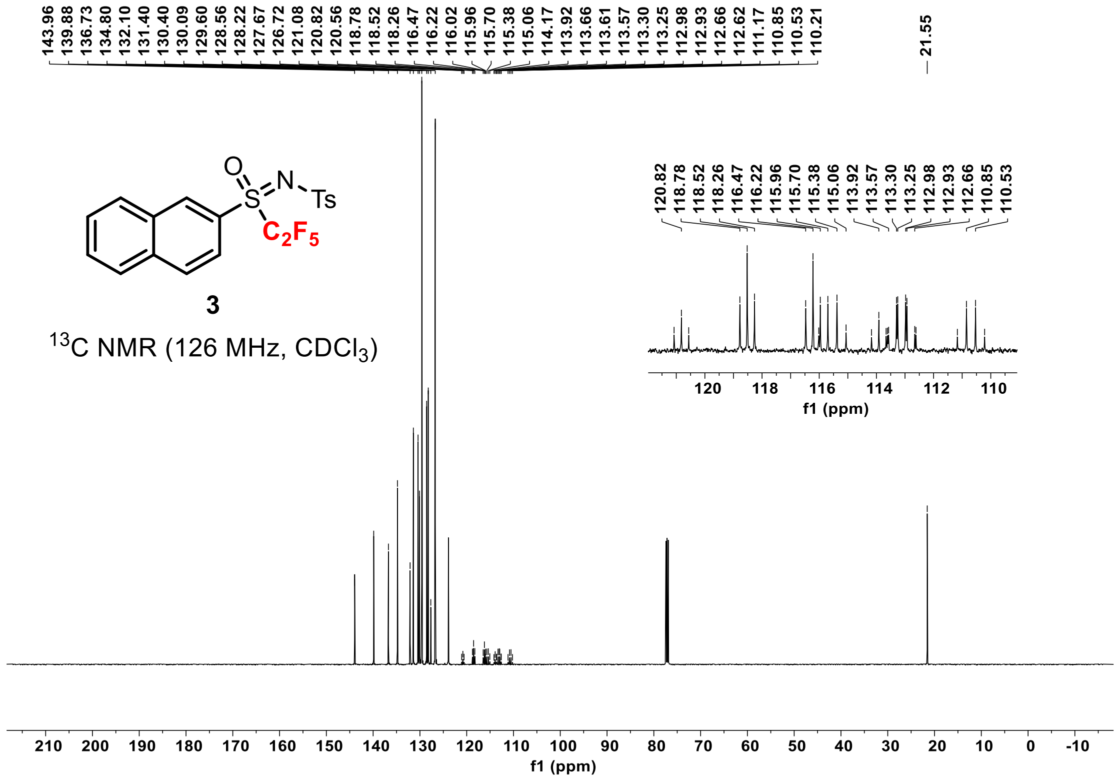
143.96
139.88
136.73
134.80
132.10
131.40
130.40
130.09
129.60
128.56
128.22
127.67
126.72
121.08
120.82
120.56
118.78
118.52
118.26
116.47
116.22
116.02
115.96
115.70
115.38
115.06
114.17
113.92
113.66
113.61
113.57
113.30
113.25
112.98
112.93
112.66
112.62
111.17
110.85
110.53
110.21
21.55
O
N
Ts
S
C2F5
3
13C NMR (126 MHz, CDCl3)
f1 (ppm)

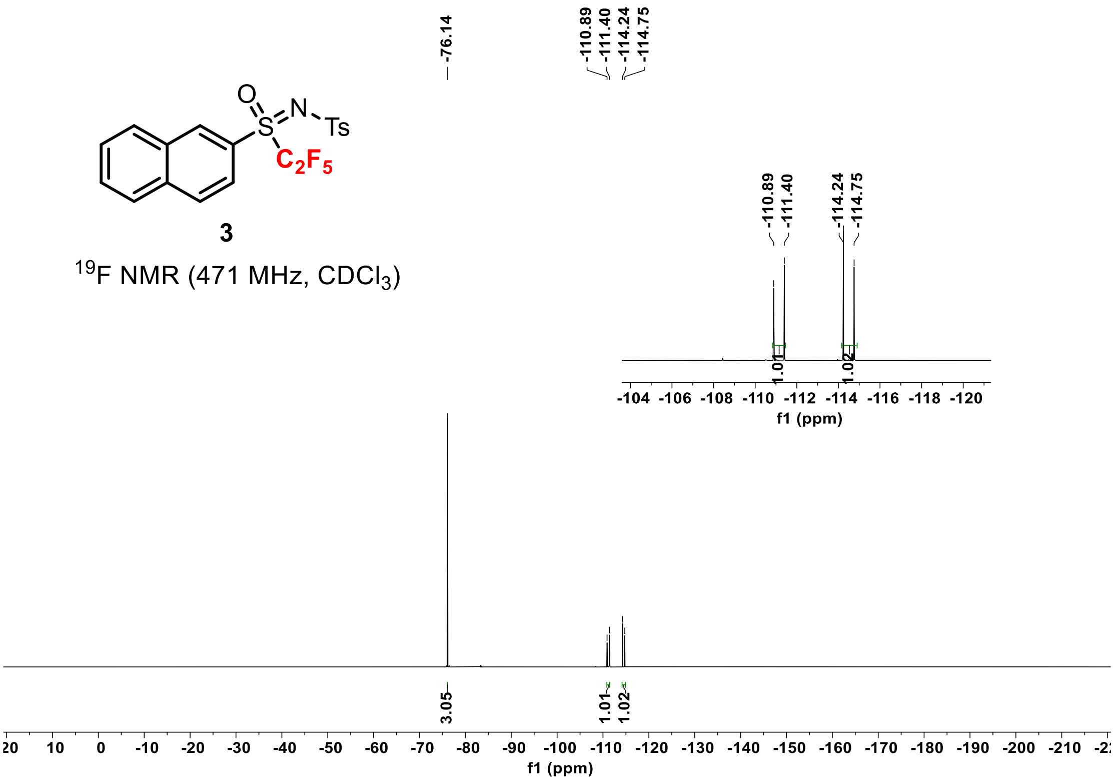

3

^{19}F NMR (471 MHz, $CDCl_3$)

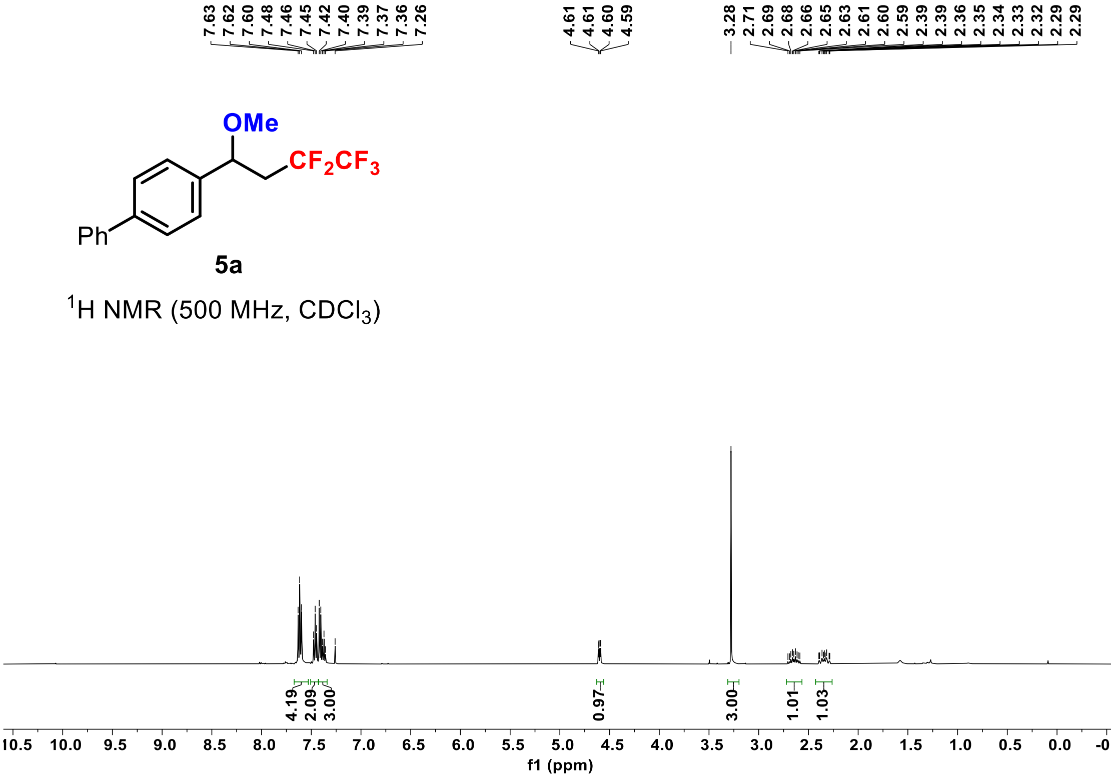

5a

^{1}H NMR (500 MHz, $CDCl_3$)

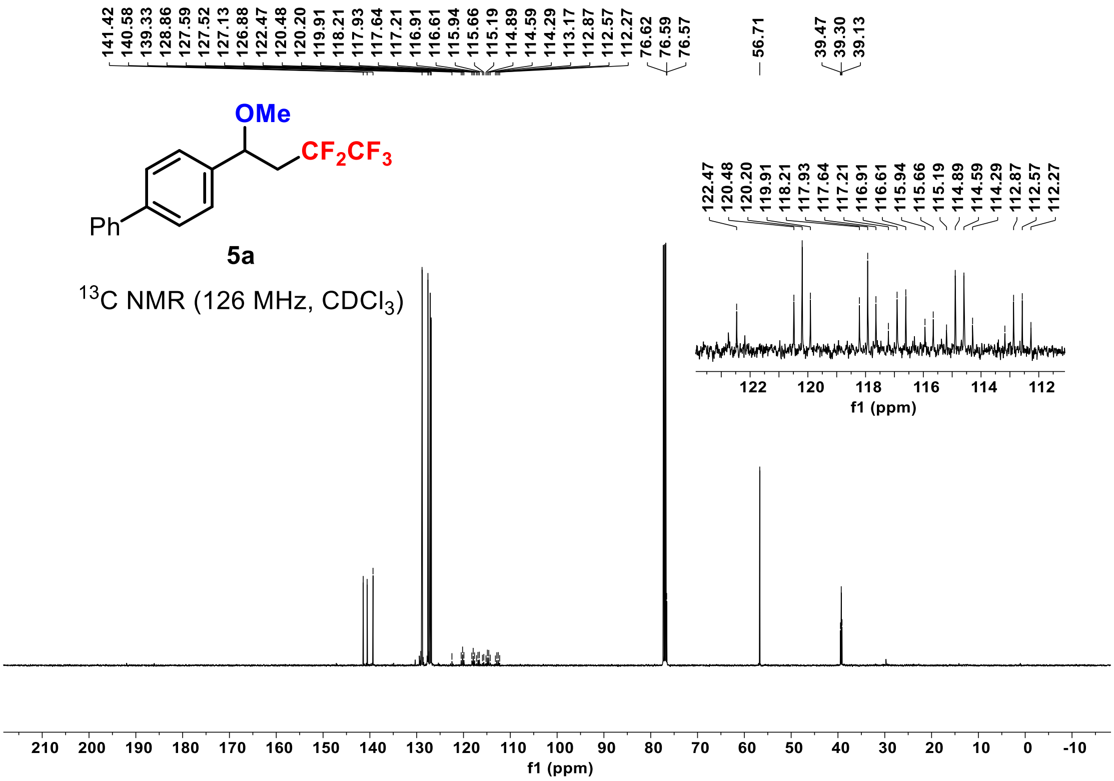
141.42
140.58
139.33
128.86
127.59
127.52
127.13
126.88
122.47
120.48
120.20
119.91
118.21
117.93
117.64
117.21
116.91
116.61
115.94
115.66
115.19
114.89
114.59
114.29
113.17
112.87
112.57
112.27
76.62
76.59
76.57
56.71
39.47
39.30
39.13
OMe
CF2CF3
Ph
5a
13C NMR (126 MHz, CDCl3)
f1 (ppm)

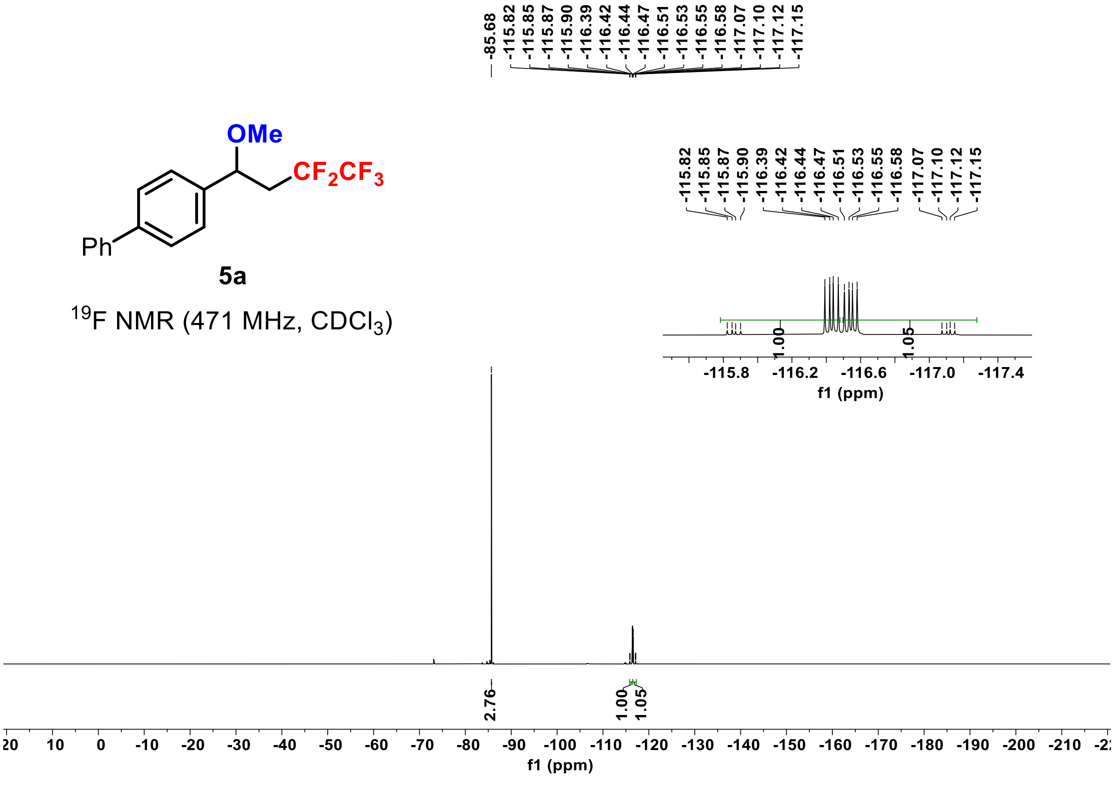
-85.68
-115.82
-115.85
-115.87
-115.90
-116.39
-116.42
-116.44
-116.47
-116.51
-116.53
-116.55
-116.58
-117.07
-117.10
-117.12
-117.15
OMe
CF2CF3
Ph
5a
19F NMR (471 MHz, CDCl3)
1.00
1.05
2.76
f1 (ppm)

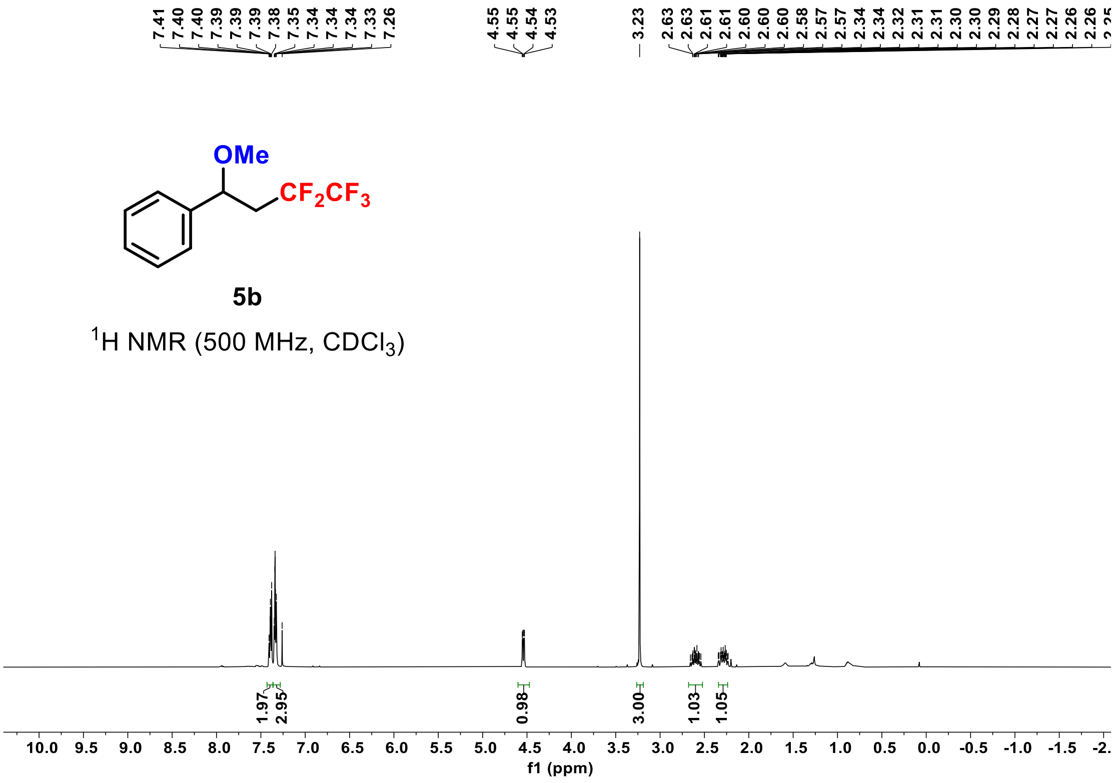

5b

^{1}H NMR (500 MHz, $CDCl_3$)

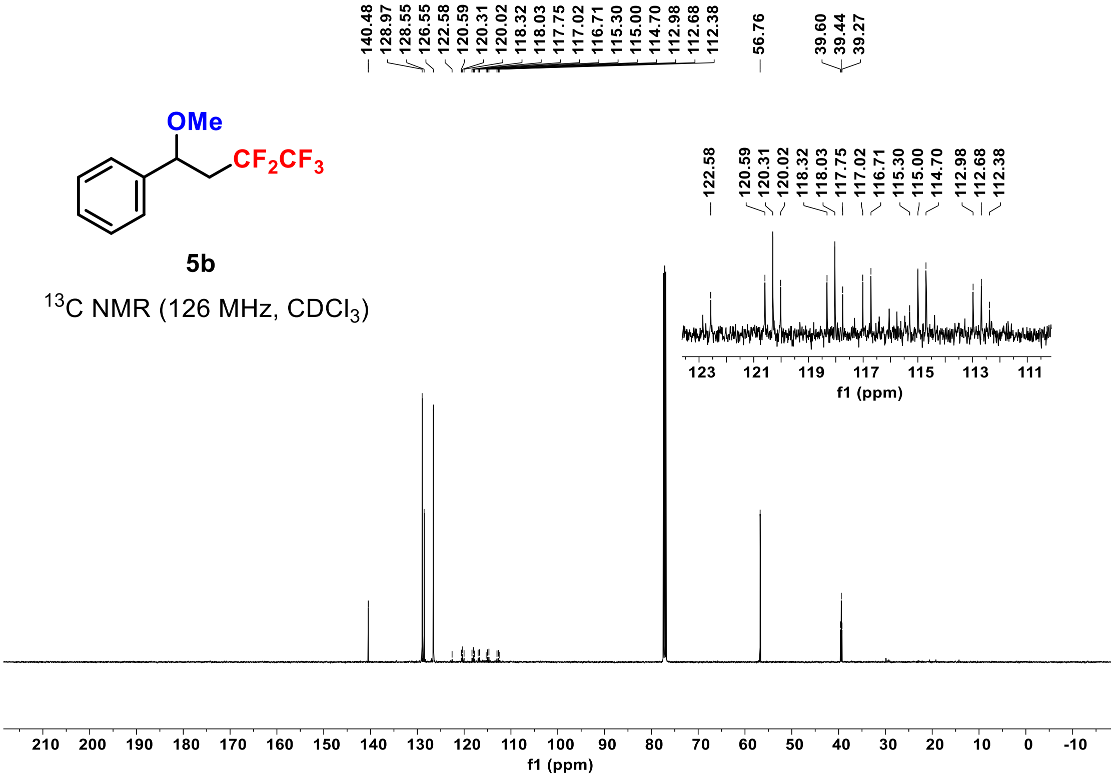

5b

^{13}C NMR (126 MHz, $CDCl_3$)

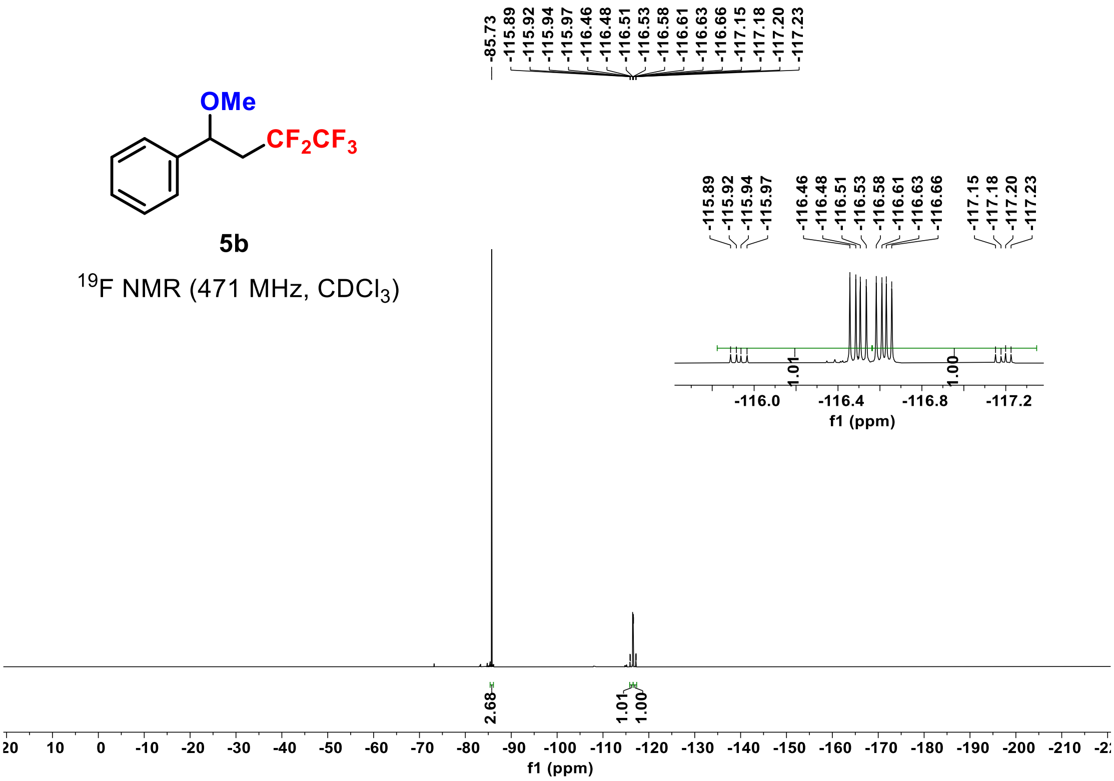

5b

^{19}F NMR (471 MHz, $CDCl_3$)

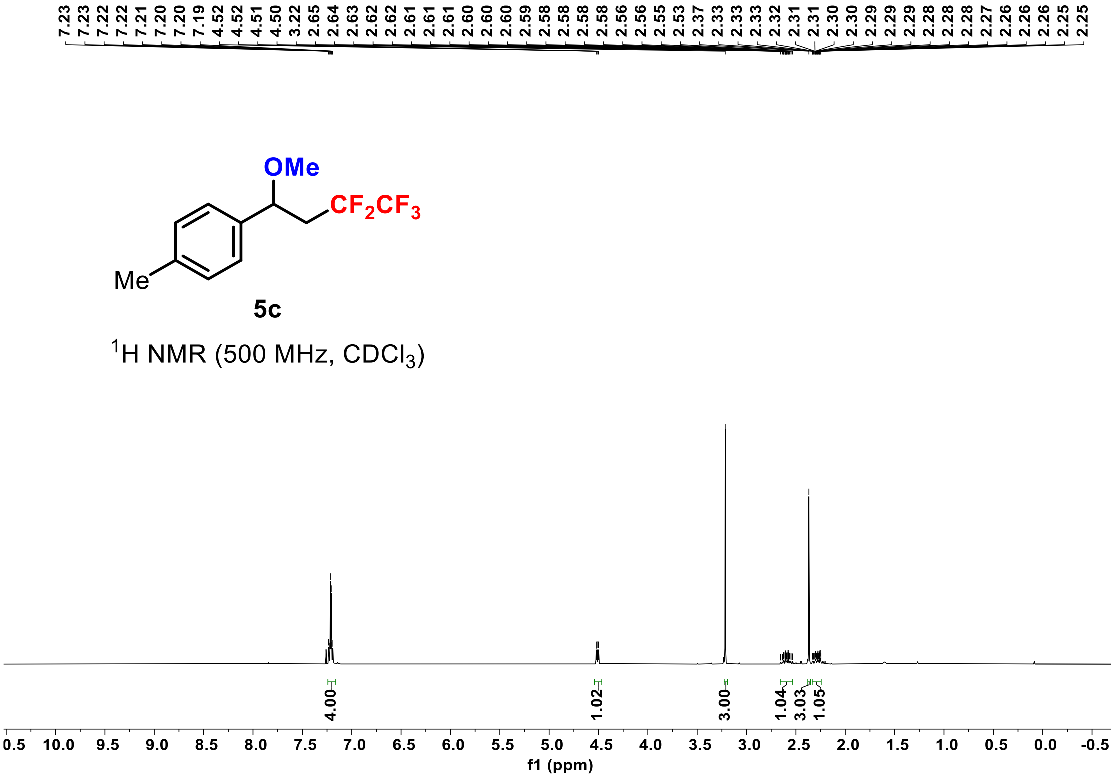

5c

^{1}H NMR (500 MHz, $CDCl_3$)

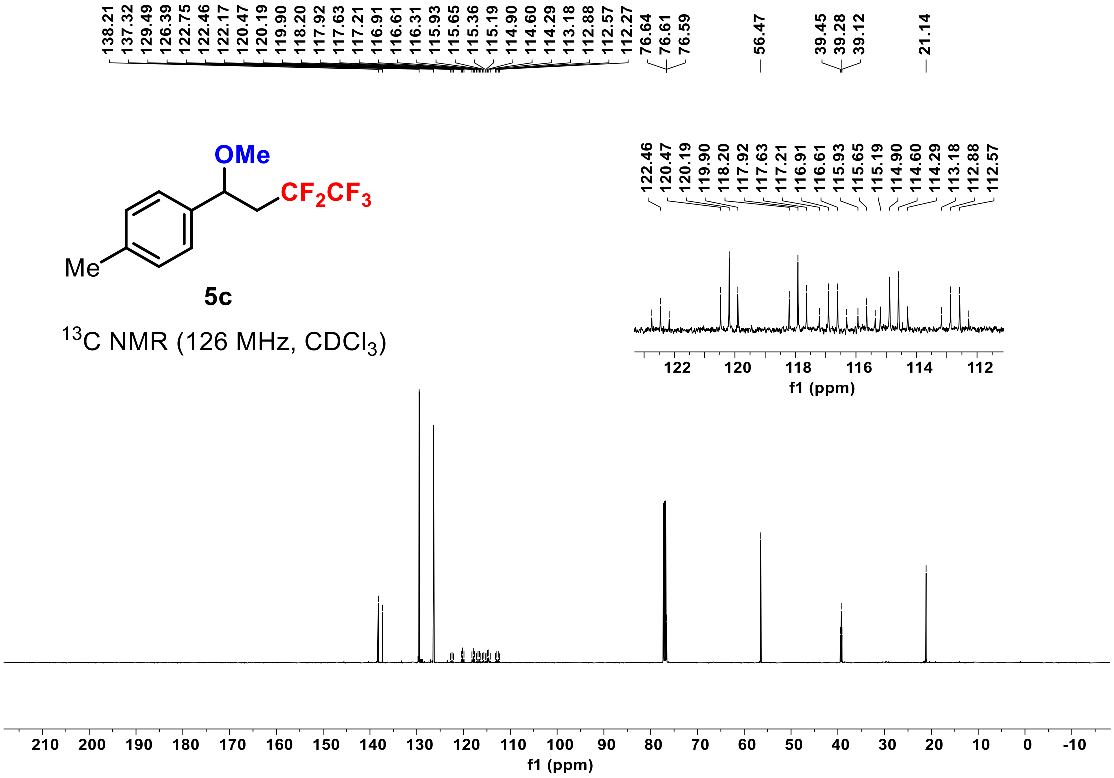
138.21
137.32
129.49
126.39
122.75
122.46
122.17
120.47
120.19
119.90
118.20
117.92
117.63
117.21
116.91
116.61
116.31
115.93
115.65
115.36
115.19
114.90
114.60
114.29
113.18
112.88
112.57
112.27
76.64
76.61
76.59
56.47
39.45
39.28
39.12
21.14
OMe
CF2CF3
Me
5c
13C NMR (126 MHz, CDCl3)
f1 (ppm)

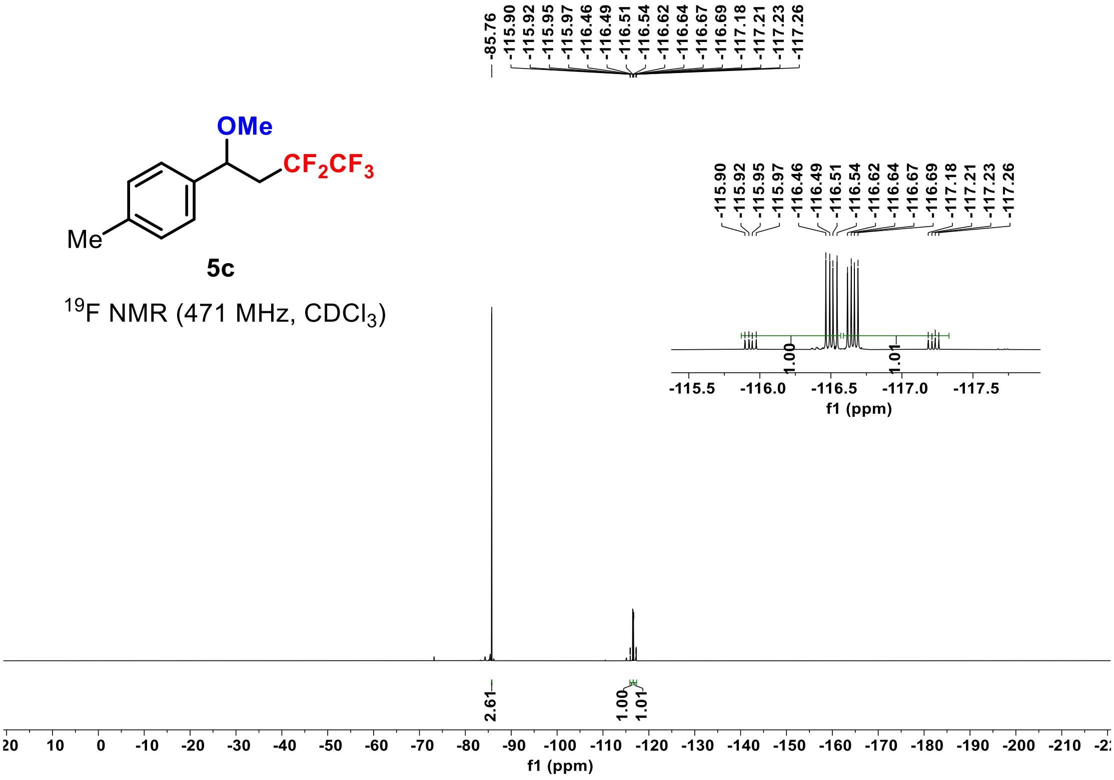
-85.76
-115.90
-115.92
-115.95
-115.97
-116.46
-116.49
-116.51
-116.54
-116.62
-116.64
-116.67
-116.69
-117.18
-117.21
-117.23
-117.26
OMe
CF2CF3
Me
5c
19F NMR (471 MHz, CDCl3)
2.61
1.00
1.01
f1 (ppm)

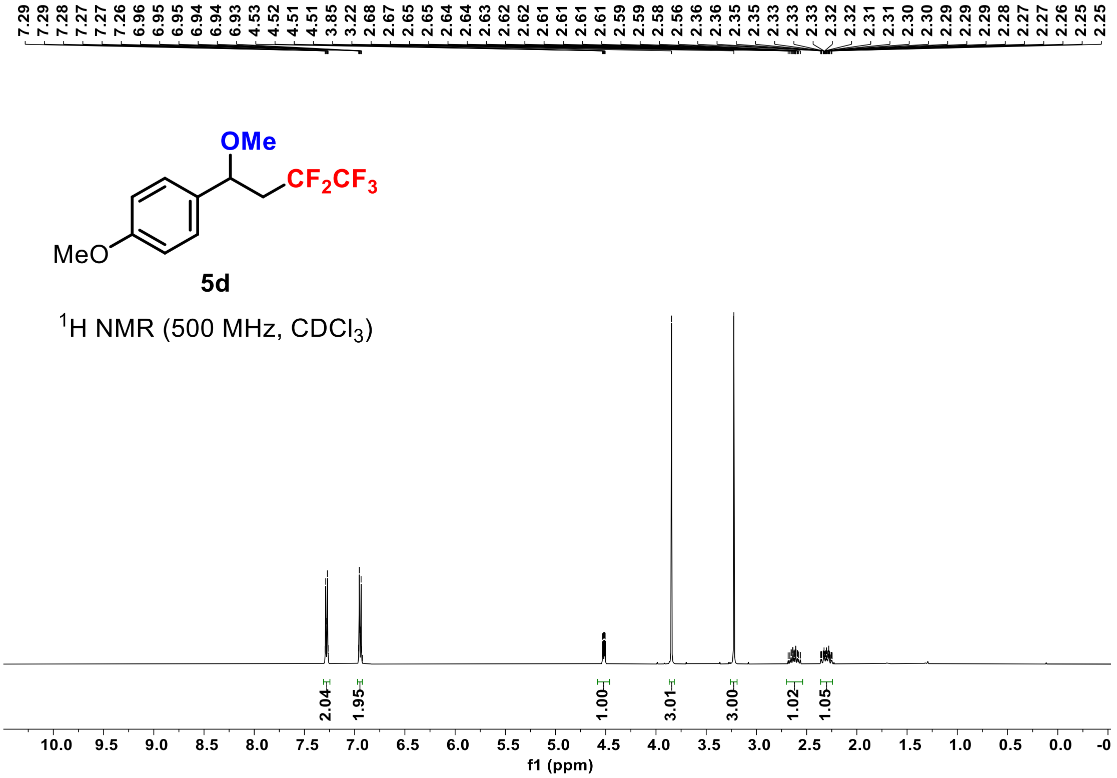
7.29 7.29 7.28 7.27 7.27 7.26 6.96 6.95 6.95 6.94 6.94 6.93 4.53 4.52 4.51 4.51 3.85 3.22 2.68 2.67 2.65 2.65 2.64 2.63 2.62 2.62 2.61 2.61 2.61 2.59 2.59 2.58 2.56 2.36 2.36 2.35 2.35 2.33 2.33 2.33 2.32 2.32 2.31 2.30 2.29 2.29 2.29 2.28 2.27 2.27 2.26 2.25 2.25
OMe
CF2CF3
MeO
5d
1H NMR (500 MHz, CDCl3)
2.04 1.95 1.00 3.01 3.00 1.02 1.05
10.0 9.5 9.0 8.5 8.0 7.5 7.0 6.5 6.0 5.5 5.0 4.5 4.0 3.5 3.0 2.5 2.0 1.5 1.0 0.5 0.0 -0
f1 (ppm)

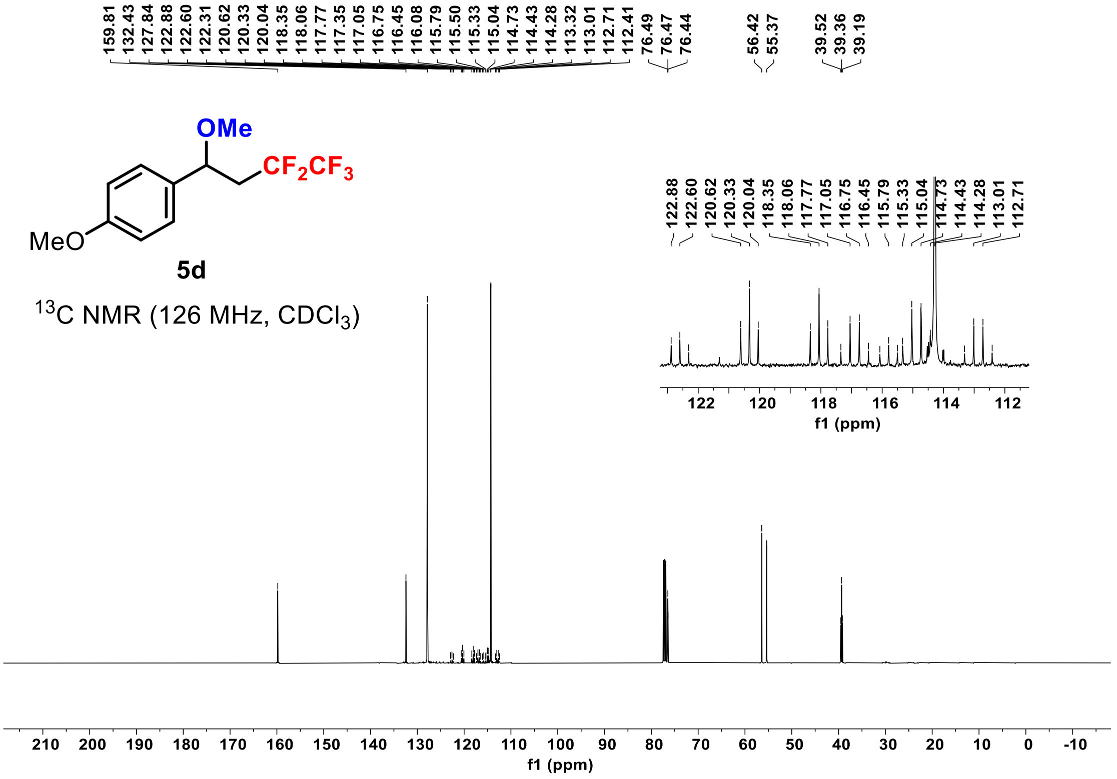
159.81 132.43 127.84 122.88 122.60 122.31 120.62 120.33 120.04 118.35 118.06 117.77 117.35 117.05 116.75 116.45 116.08 115.79 115.50 115.33 115.04 114.73 114.43 114.28 113.32 113.01 112.71 112.41 76.49 76.47 76.44 56.42 55.37 39.52 39.36 39.19
OMe
CF2CF3
MeO
5d
13C NMR (126 MHz, CDCl3)
122.88 122.60 120.62 120.33 120.04 118.35 118.06 117.77 117.05 116.75 116.45 115.79 115.33 115.04 114.73 114.43 114.28 113.01 112.71
122 120 118 116 114 112
f1 (ppm)
210 200 190 180 170 160 150 140 130 120 110 100 90 80 70 60 50 40 30 20 10 0 -10
f1 (ppm)

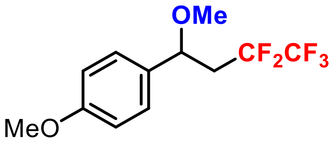

5d

^{19}F NMR (471 MHz, $CDCl_3$)

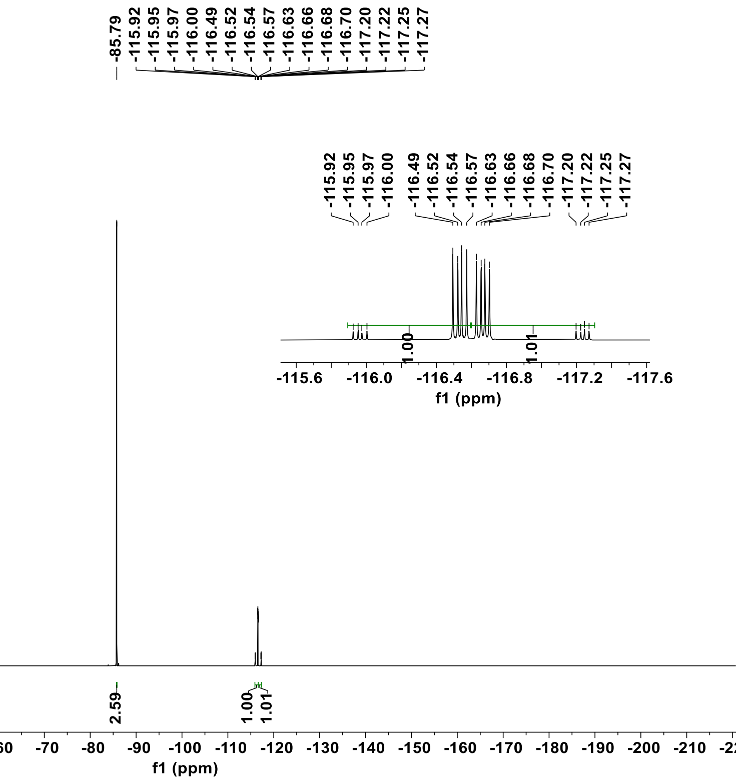

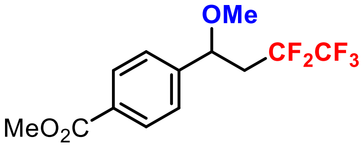

5e

^{1}H NMR (500 MHz, $CDCl_3$)

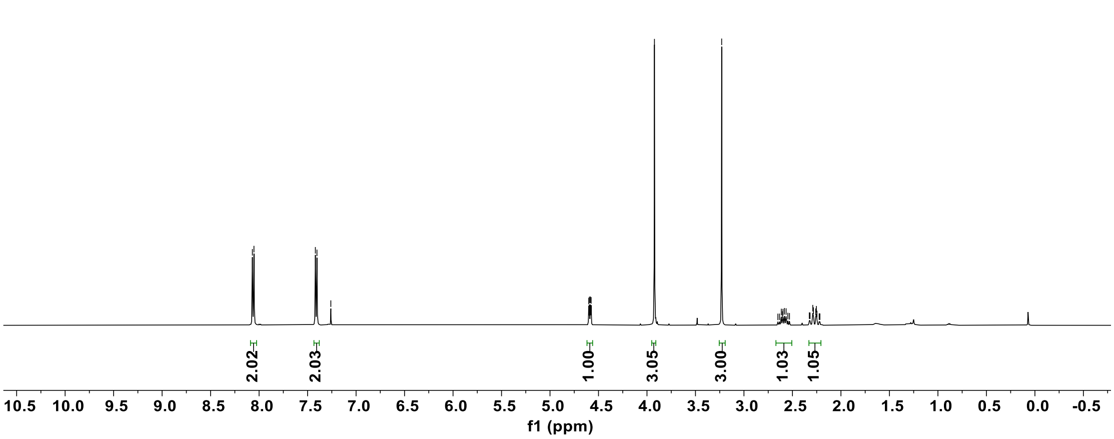

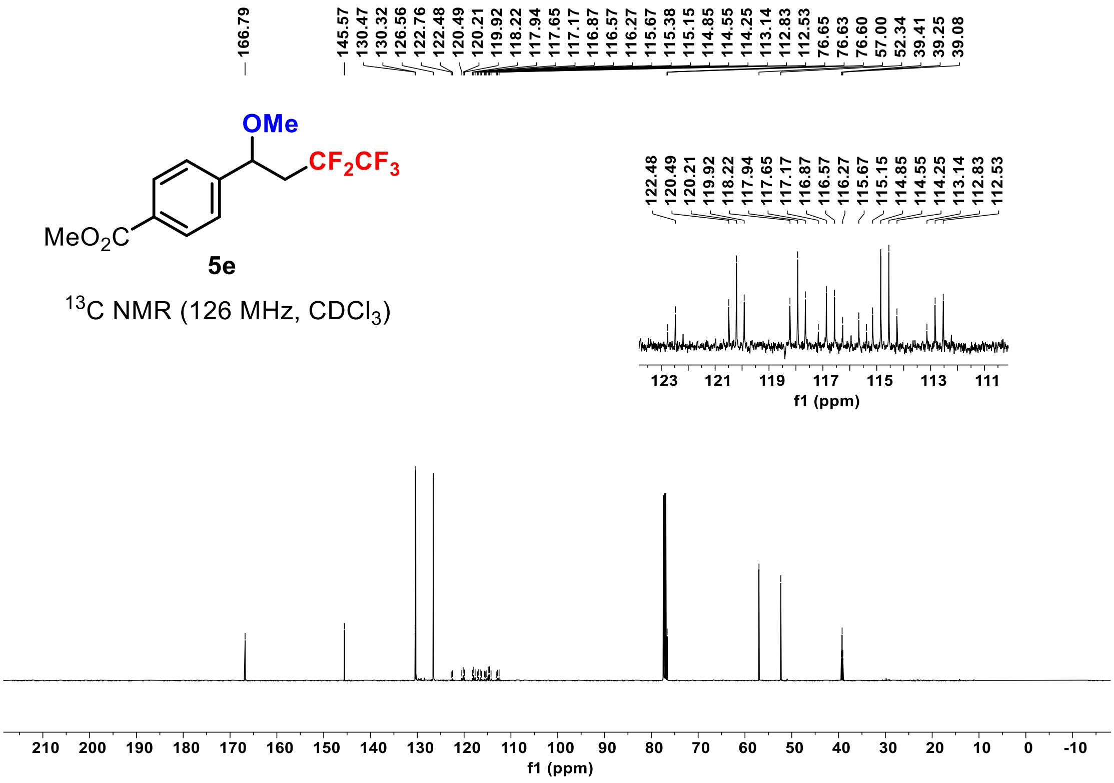

OMe
CF2CF3
MeO2C
5e
13C NMR (126 MHz, CDCl3)
f1 (ppm)

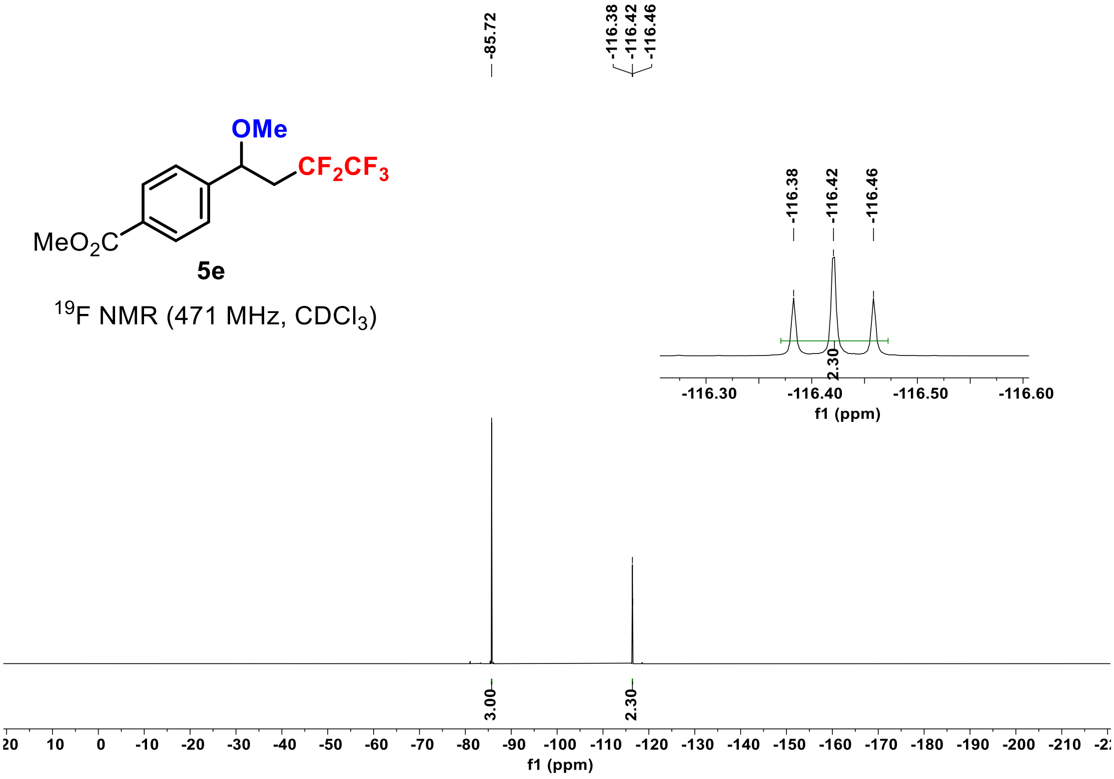

OMe
CF2CF3
MeO2C
5e
19F NMR (471 MHz, CDCl3)
f1 (ppm)

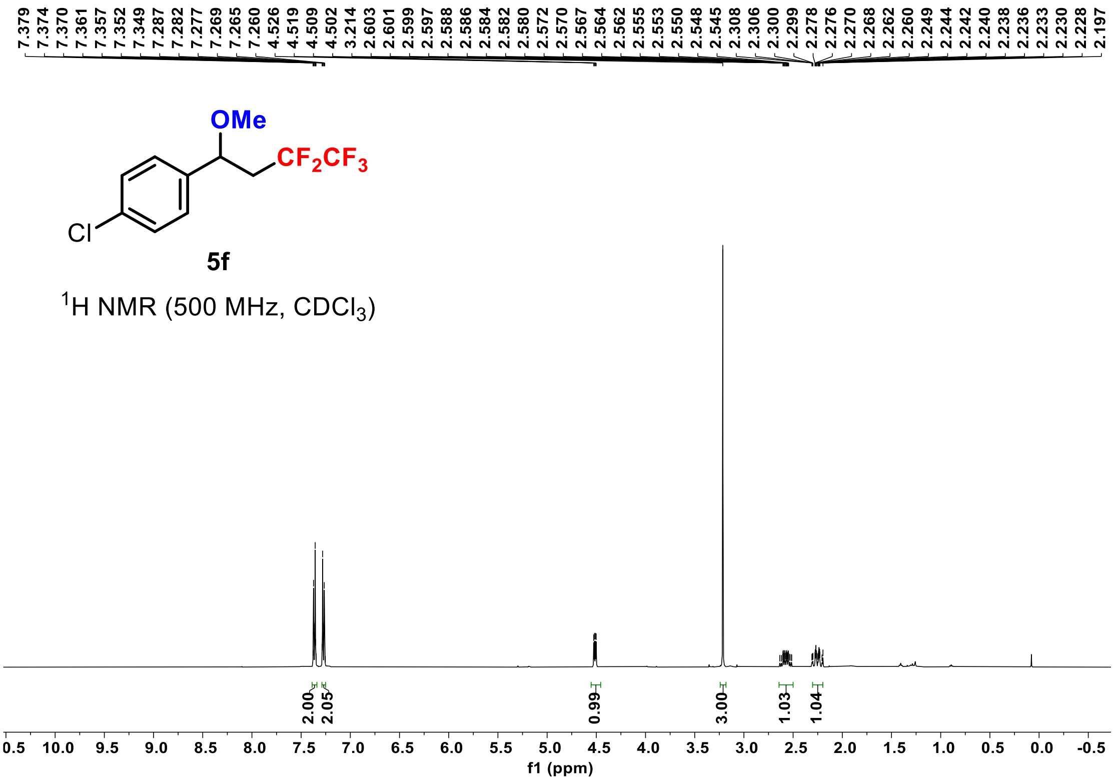
OMe
CF2CF3
Cl
5f
1H NMR (500 MHz, CDCl3)
f1 (ppm)

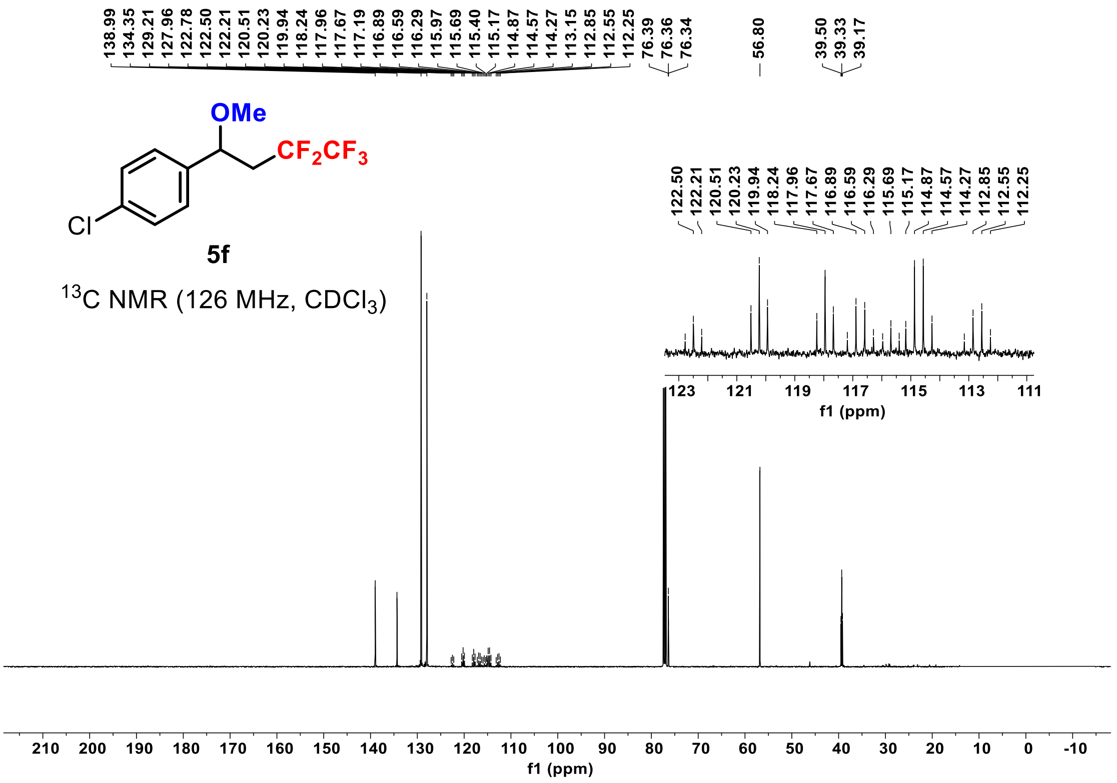
OMe
CF2CF3
Cl
5f
13C NMR (126 MHz, CDCl3)
f1 (ppm)

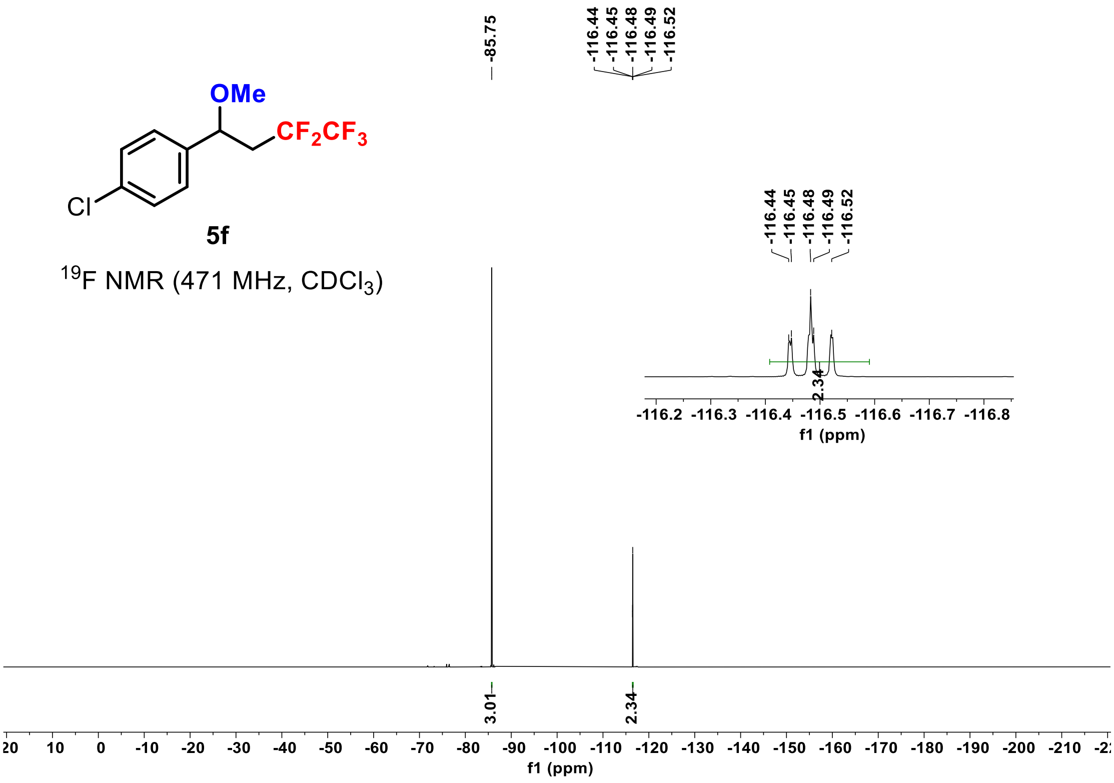
OMe
CF2CF3
Cl
5f
19F NMR (471 MHz, CDCl3)
-85.75
-116.44
-116.45
-116.48
-116.49
-116.52
2.34
3.01
-116.2 -116.3 -116.4 -116.5 -116.6 -116.7 -116.8
f1 (ppm)
20 10 0 -10 -20 -30 -40 -50 -60 -70 -80 -90 -100 -110 -120 -130 -140 -150 -160 -170 -180 -190 -200 -210
f1 (ppm)

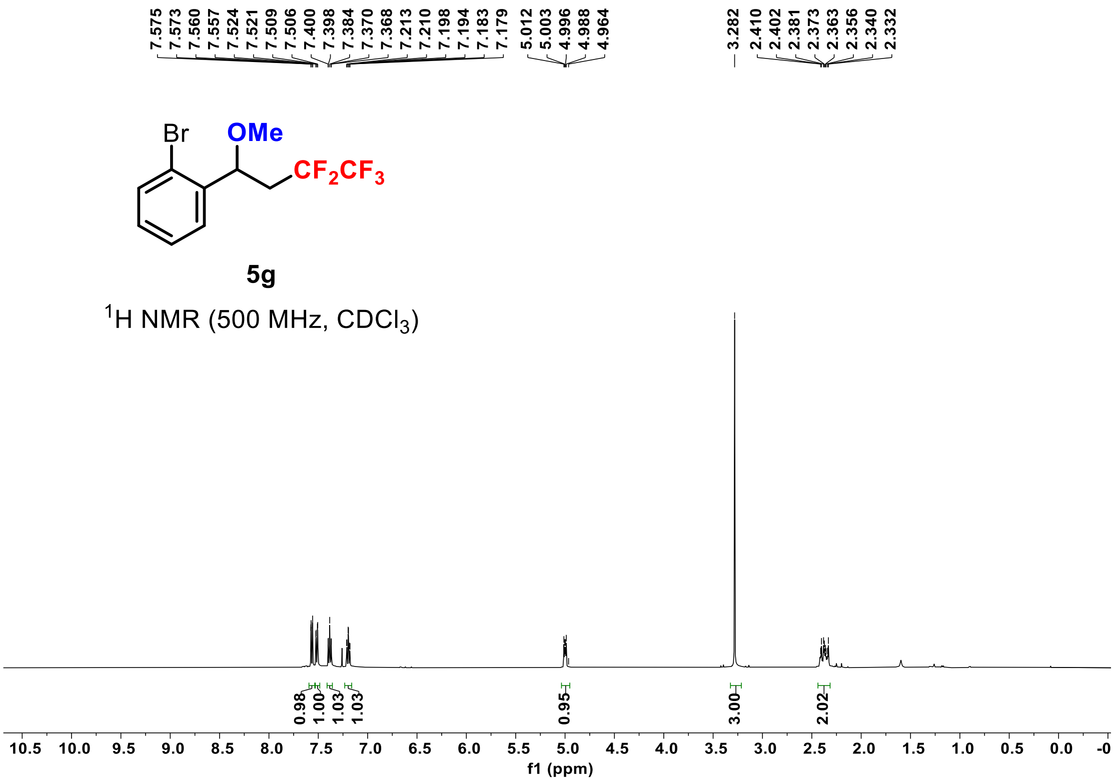
Br
OMe
CF2CF3
5g
1H NMR (500 MHz, CDCl3)
7.575 7.573 7.560 7.557 7.524 7.521 7.509 7.506 7.400 7.398 7.384 7.370 7.368 7.213 7.210 7.198 7.194 7.183 7.179
5.012 5.003 4.996 4.988 4.964
3.282
2.410 2.402 2.381 2.373 2.363 2.356 2.340 2.332
0.98 1.00 1.03 1.03
0.95
3.00
2.02
10.5 10.0 9.5 9.0 8.5 8.0 7.5 7.0 6.5 6.0 5.5 5.0 4.5 4.0 3.5 3.0 2.5 2.0 1.5 1.0 0.5 0.0
f1 (ppm)

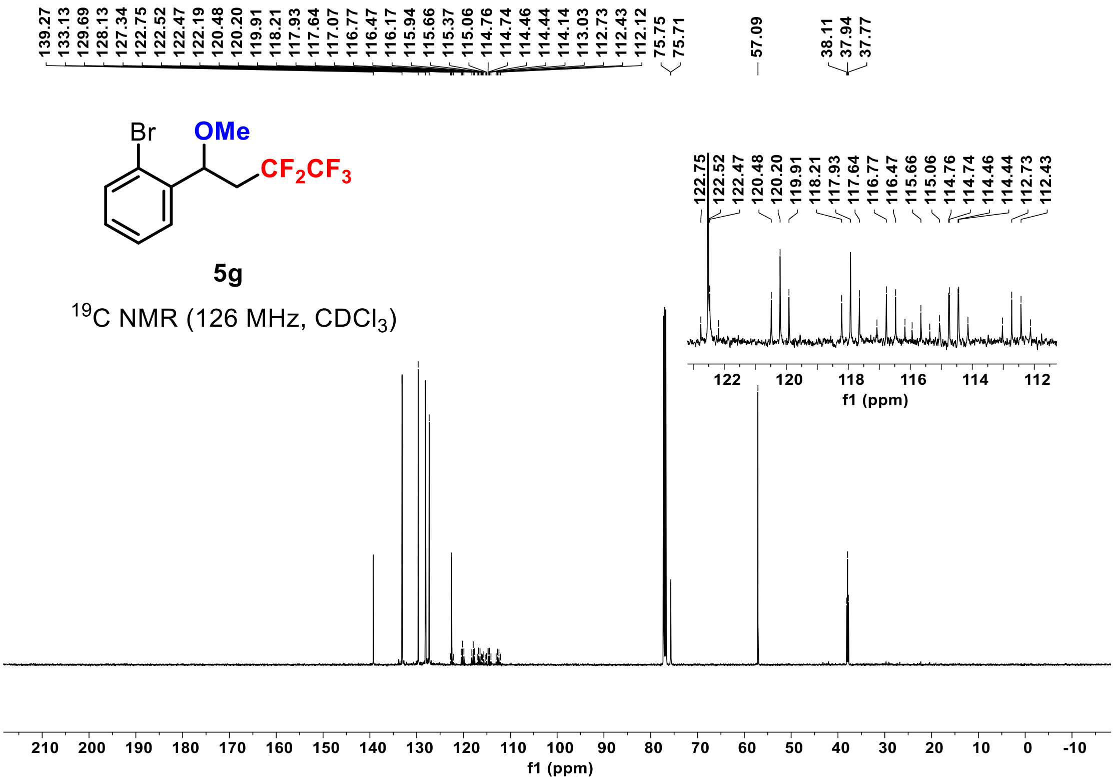
139.27
133.13
129.69
128.13
127.34
122.75
122.52
122.47
122.19
120.48
120.20
119.91
118.21
117.93
117.64
117.07
116.77
116.47
116.17
115.94
115.66
115.37
115.06
114.76
114.74
114.46
114.44
114.14
113.03
112.73
112.43
112.12
75.75
75.71
57.09
38.11
37.94
37.77
Br
OMe
CF2CF3
5g
19C NMR (126 MHz, CDCl3)
f1 (ppm)

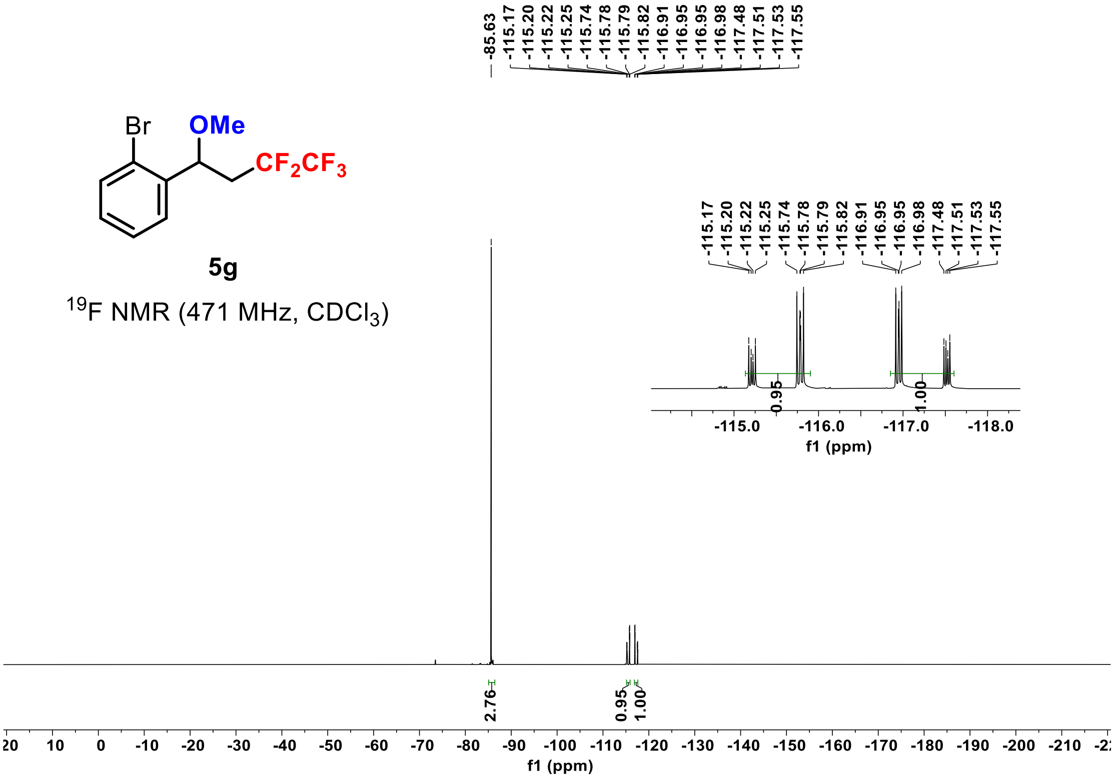
-85.63
-115.17
-115.20
-115.22
-115.25
-115.74
-115.78
-115.79
-115.82
-116.91
-116.95
-116.95
-116.98
-117.48
-117.51
-117.53
-117.55
Br
OMe
CF2CF3
5g
19F NMR (471 MHz, CDCl3)
0.95
1.00
2.76
f1 (ppm)

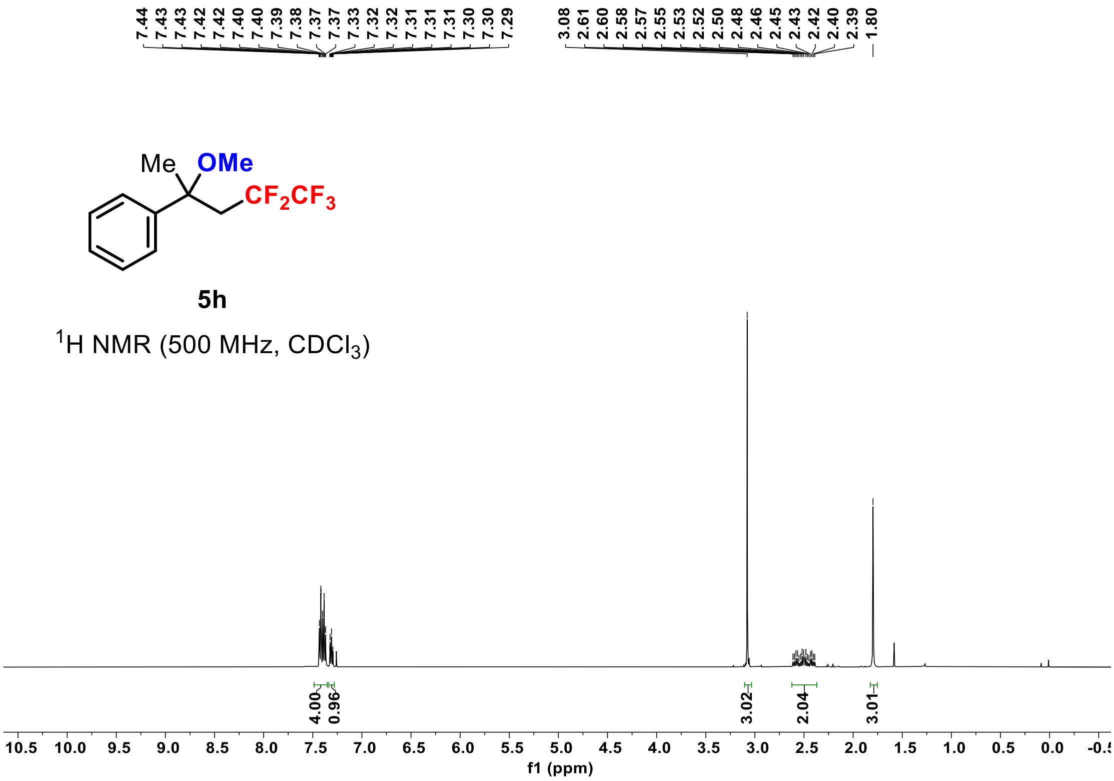

7.44 7.43 7.43 7.42 7.42 7.40 7.39 7.38 7.37 7.37 7.33 7.32 7.32 7.31 7.31 7.30 7.29
3.08 2.61 2.60 2.58 2.57 2.55 2.53 2.52 2.50 2.48 2.46 2.45 2.43 2.42 2.40 2.39 1.80
Me
OMe
CF2CF3
5h
1H NMR (500 MHz, CDCl3)
4.00 0.96 3.02 2.04 3.01
10.5 10.0 9.5 9.0 8.5 8.0 7.5 7.0 6.5 6.0 5.5 5.0 4.5 4.0 3.5 3.0 2.5 2.0 1.5 1.0 0.5 0.0
f1 (ppm)

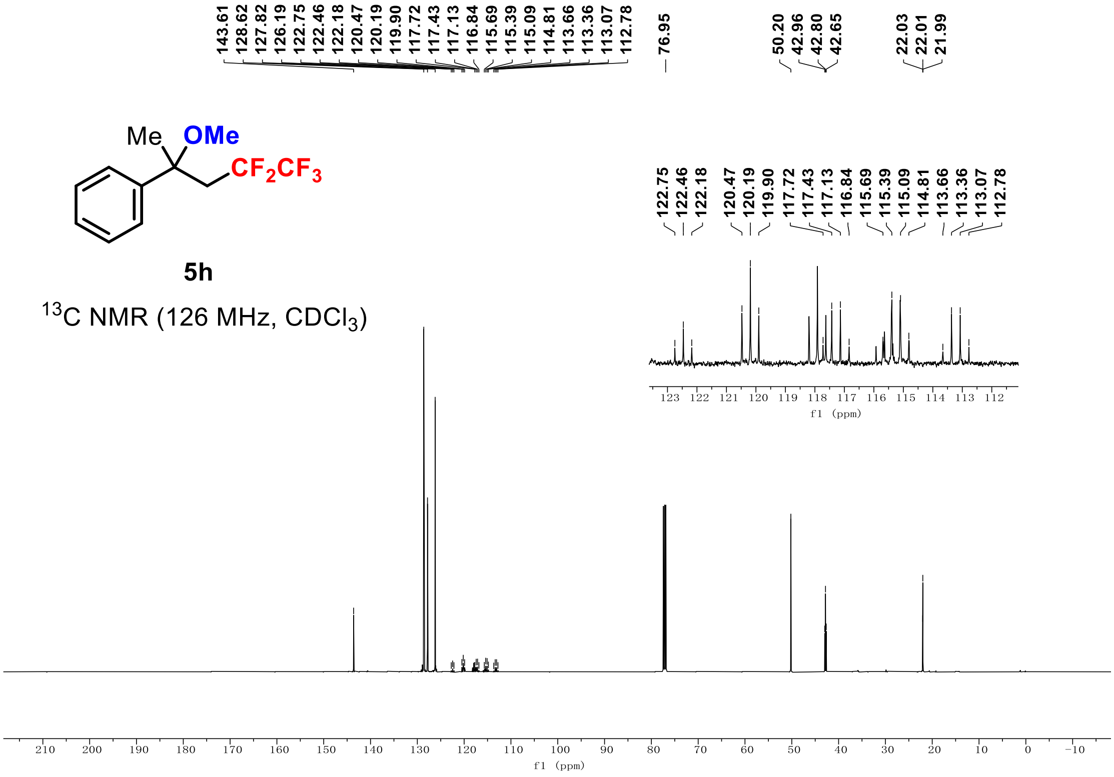

143.61 128.62 127.82 126.19 122.75 122.46 122.18 120.47 120.19 119.90 117.72 117.43 117.13 116.84 115.69 115.39 115.09 114.81 113.66 113.36 113.07 112.78
76.95
50.20 42.96 42.80 42.65
22.03 22.01 21.99
Me
OMe
CF2CF3
5h
13C NMR (126 MHz, CDCl3)
122.75 122.46 122.18 120.47 120.19 119.90 117.72 117.43 117.13 116.84 115.69 115.39 115.09 114.81 113.66 113.36 113.07 112.78
123 122 121 120 119 118 117 116 115 114 113 112
f1 (ppm)
210 200 190 180 170 160 150 140 130 120 110 100 90 80 70 60 50 40 30 20 10 0 -10
f1 (ppm)

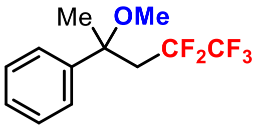

5h

^{19}F NMR (471 MHz, $CDCl_3$)

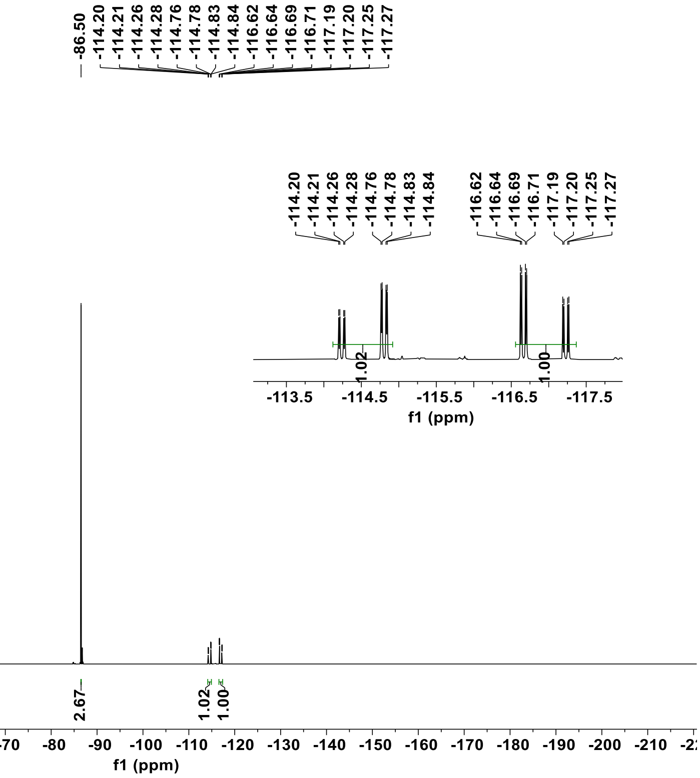

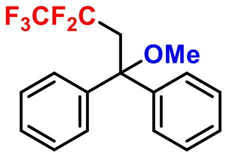

5i

^{1}H NMR (500 MHz, $CDCl_3$)

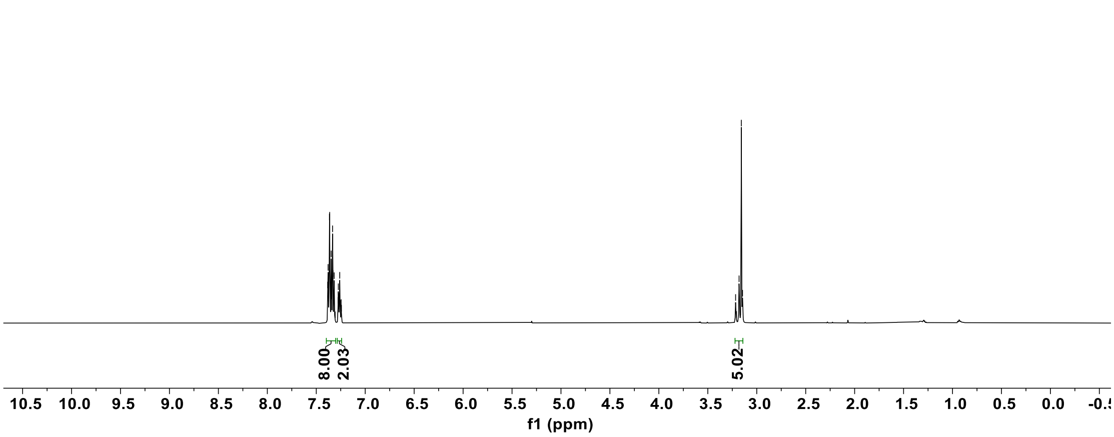

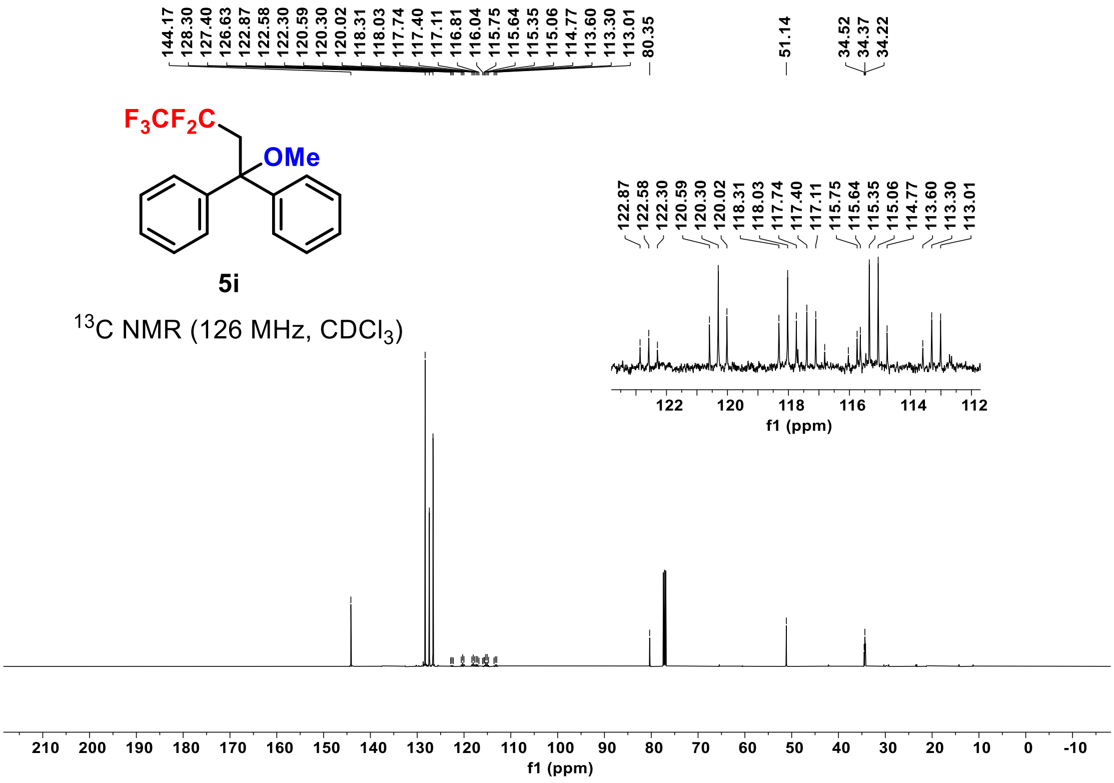

144.17
128.30
127.40
126.63
122.87
122.58
122.30
120.59
120.30
120.02
118.31
118.03
117.74
117.40
117.11
116.81
116.04
115.75
115.64
115.35
115.06
114.77
113.60
113.30
113.01
80.35
51.14
34.52
34.37
34.22
F3CF2C
OMe
5i
13C NMR (126 MHz, CDCl3)
f1 (ppm)

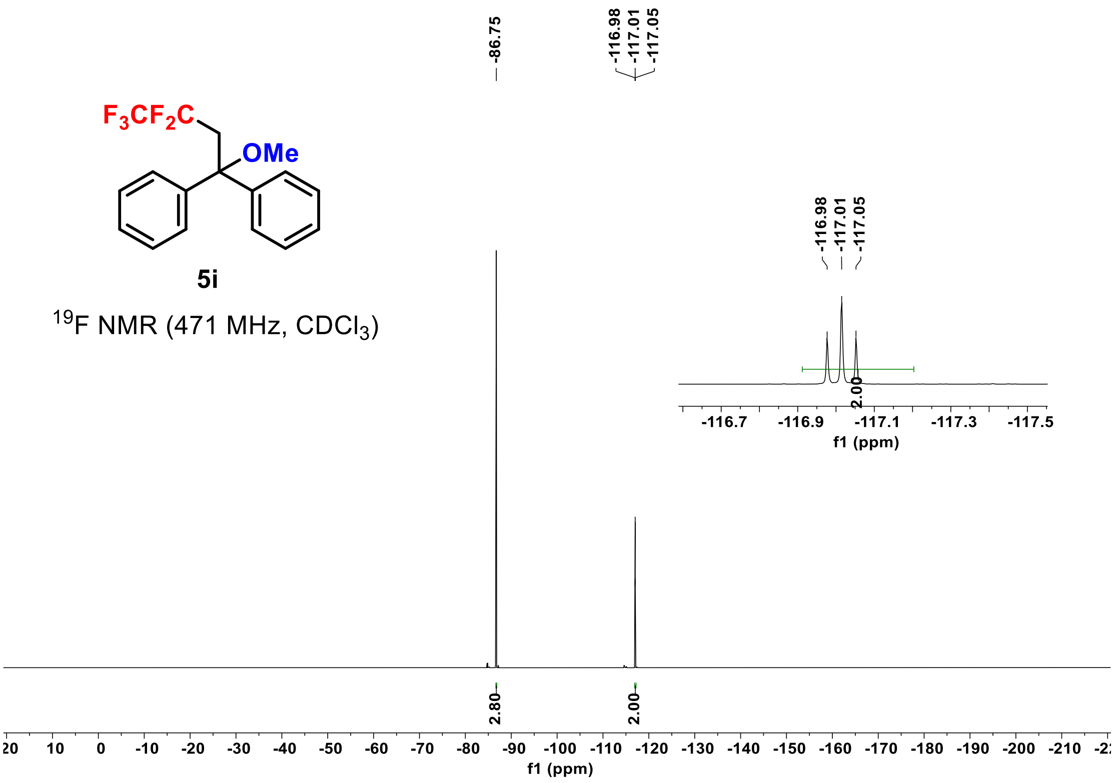

-86.75
-116.98
-117.01
-117.05
F3CF2C
OMe
5i
19F NMR (471 MHz, CDCl3)
2.00
2.80
f1 (ppm)

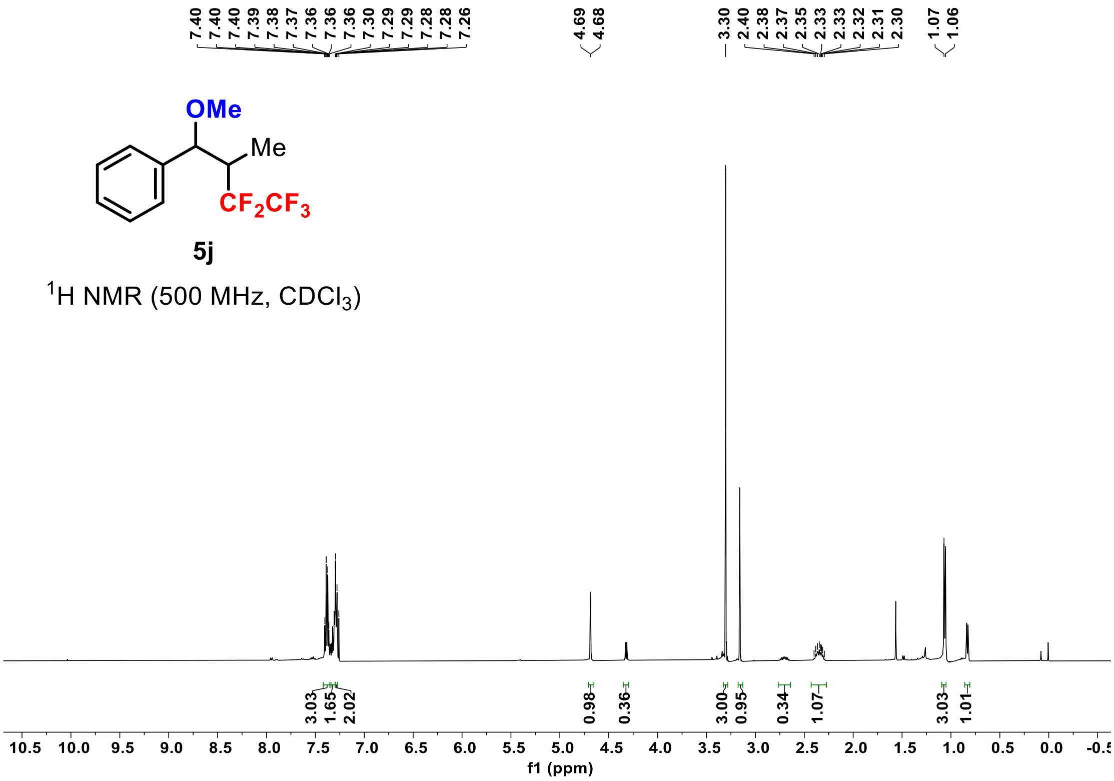
7.40 7.40 7.40 7.39 7.38 7.37 7.36 7.36 7.36 7.30 7.29 7.29 7.28 7.28 7.26
4.69 4.68
3.30 2.40 2.38 2.37 2.35 2.33 2.33 2.32 2.31 2.30
1.07 1.06
OMe
Me
CF2CF3
5j
1H NMR (500 MHz, CDCl3)
3.03 1.65 2.02
0.98 0.36 3.00 0.95 0.34 1.07 3.03 1.01
f1 (ppm)

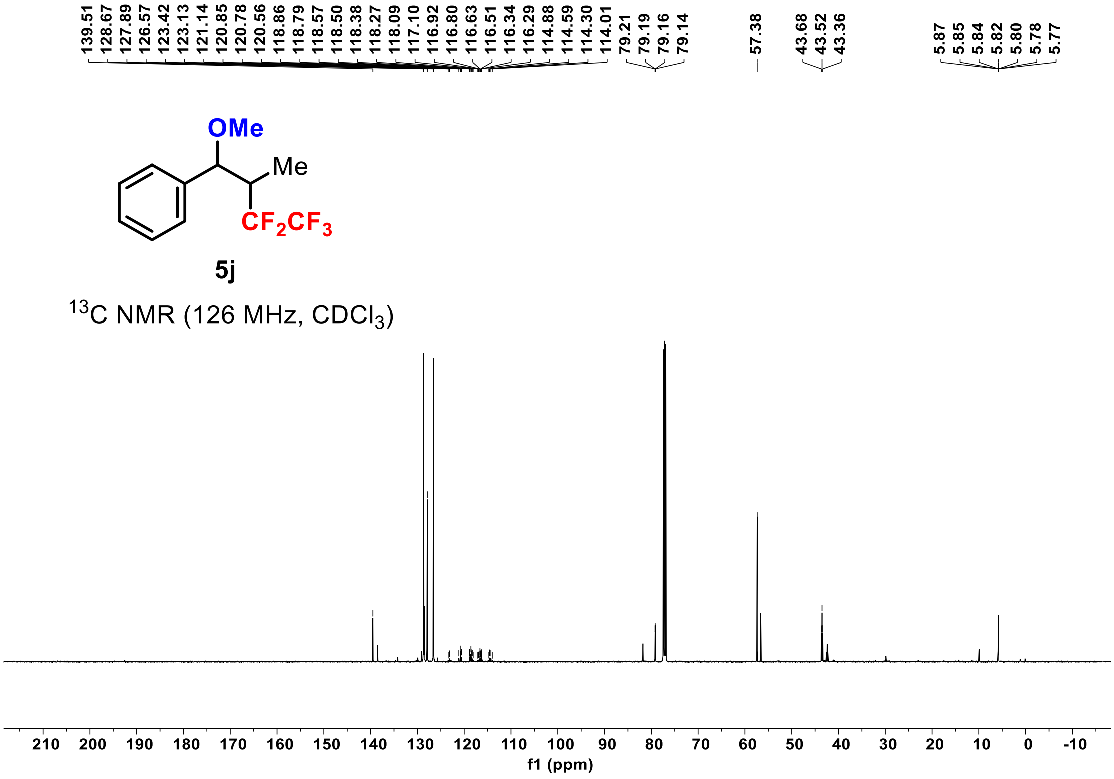
139.51 128.67 127.89 126.57 123.42 123.13 121.14 120.85 120.78 120.56 118.86 118.79 118.57 118.50 118.38 118.27 118.09 117.10 116.92 116.80 116.63 116.51 116.34 116.29 114.88 114.59 114.30 114.01
79.21 79.19 79.16 79.14
57.38
43.68 43.52 43.36
5.87 5.85 5.84 5.82 5.80 5.78 5.77
OMe
Me
CF2CF3
5j
13C NMR (126 MHz, CDCl3)
f1 (ppm)

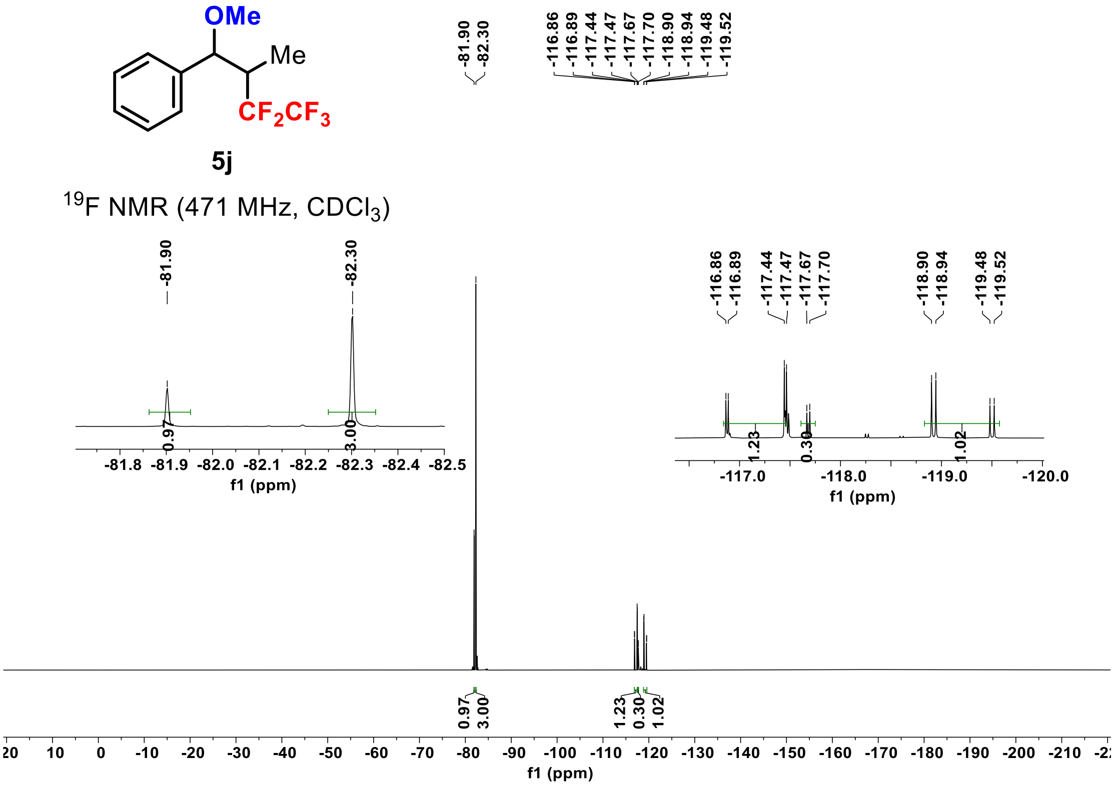
OMe
Me
CF2CF3
5j
19F NMR (471 MHz, CDCl3)
f1 (ppm)

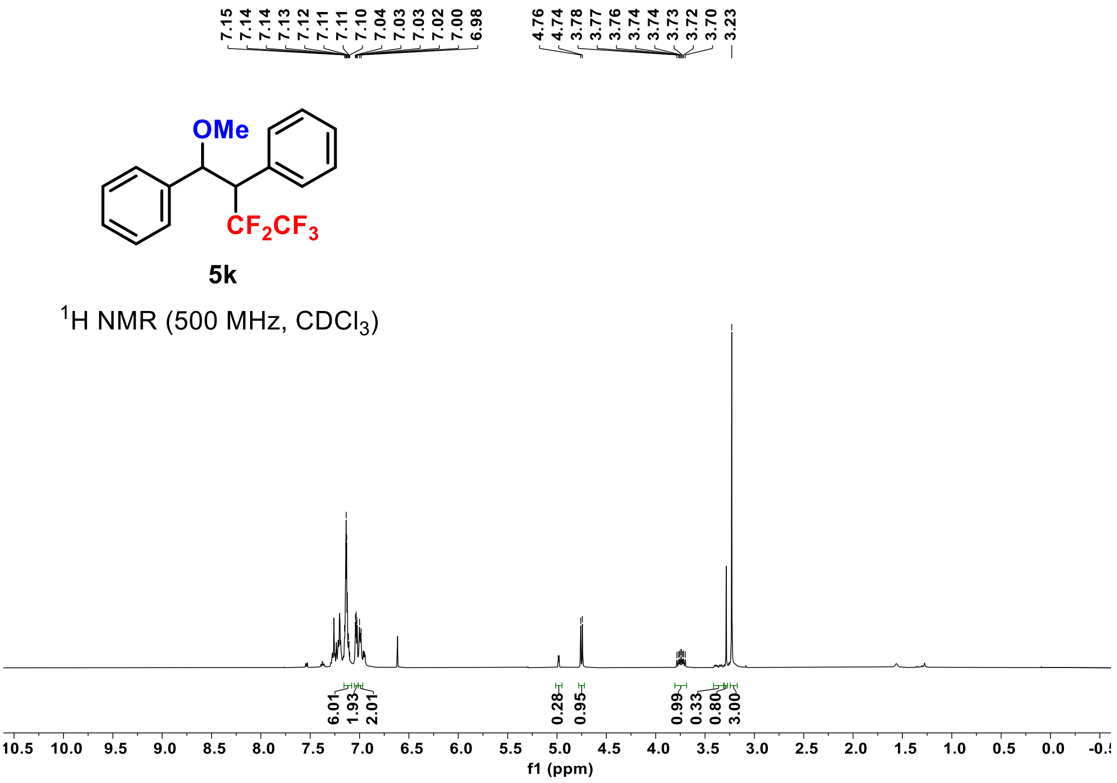
OMe
CF2CF3
5k
1H NMR (500 MHz, CDCl3)
f1 (ppm)

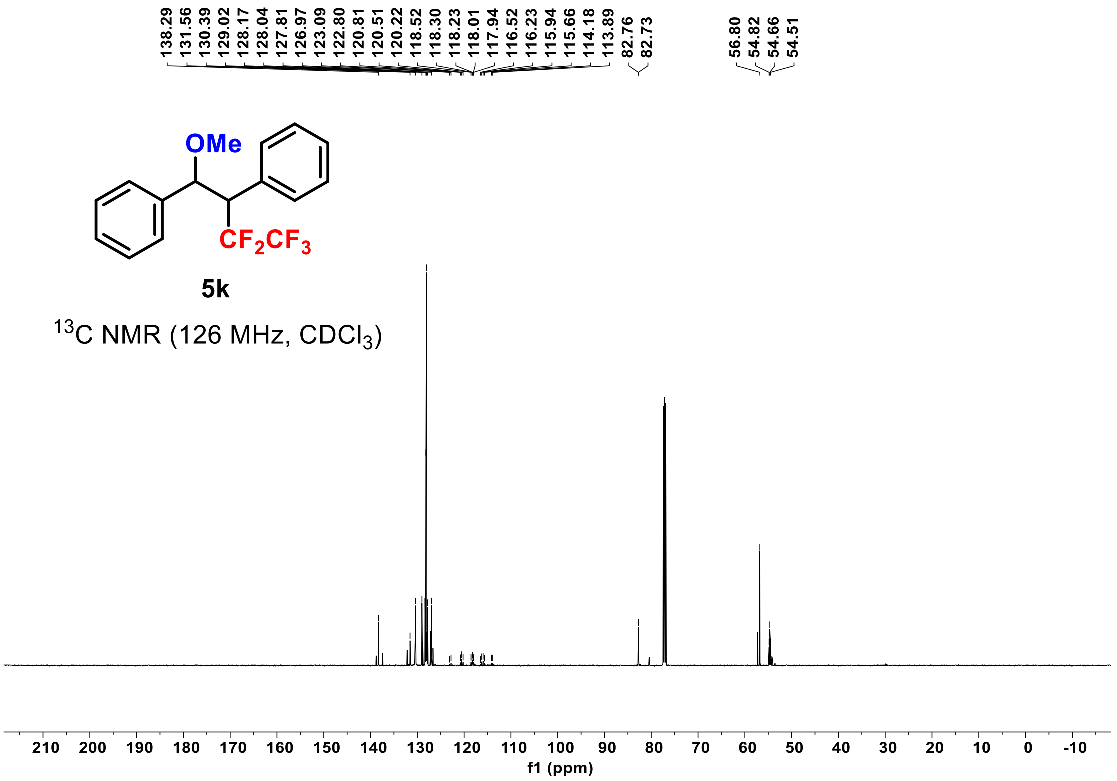
138.29
131.56
130.39
129.02
128.17
128.04
127.81
126.97
123.09
122.80
120.81
120.51
120.22
118.52
118.30
118.23
118.01
117.94
116.52
116.23
115.94
115.66
114.18
113.89
82.76
82.73
56.80
54.82
54.66
54.51
OMe
CF2CF3
5k
13C NMR (126 MHz, CDCl3)
210 200 190 180 170 160 150 140 130 120 110 100 90 80 70 60 50 40 30 20 10 0 -10
f1 (ppm)

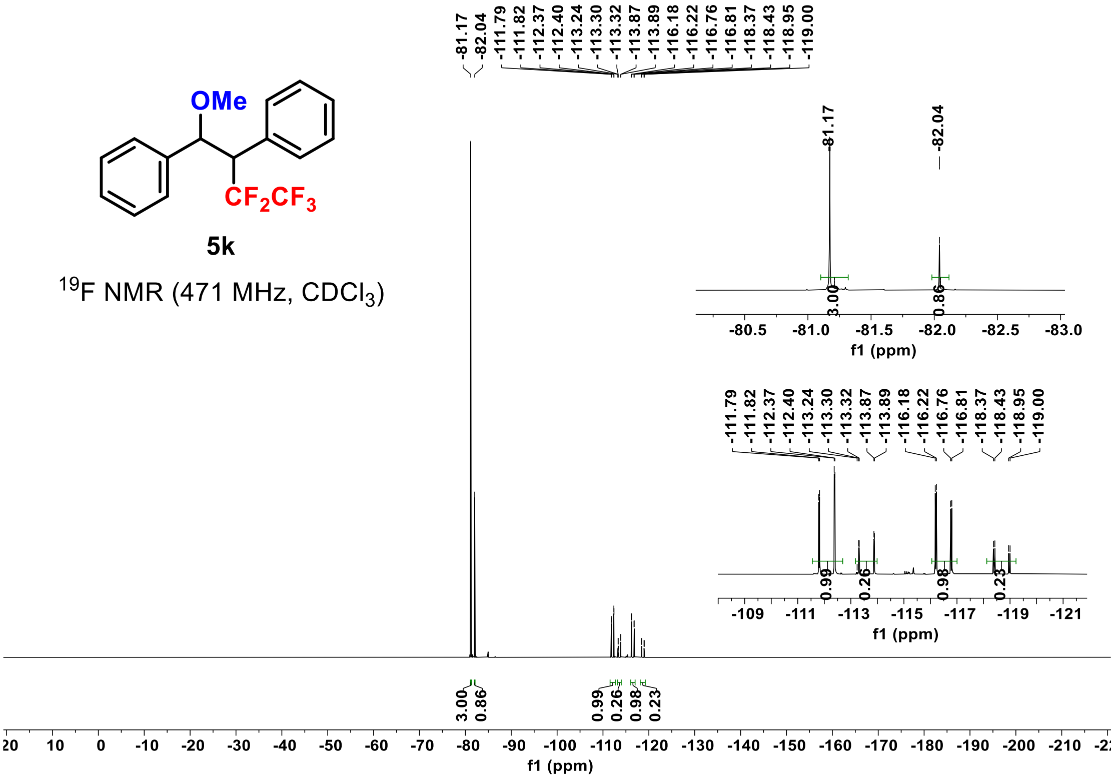
-81.17
-82.04
-111.79
-111.82
-112.37
-112.40
-113.24
-113.30
-113.32
-113.87
-113.89
-116.18
-116.22
-116.76
-116.81
-118.37
-118.43
-118.95
-119.00
OMe
CF2CF3
5k
19F NMR (471 MHz, CDCl3)
3.00
0.86
-80.5 -81.0 -81.5 -82.0 -82.5 -83.0
0.99
0.26
0.98
0.23
-109 -111 -113 -115 -117 -119 -121
f1 (ppm)
20 10 0 -10 -20 -30 -40 -50 -60 -70 -80 -90 -100 -110 -120 -130 -140 -150 -160 -170 -180 -190 -200 -210
f1 (ppm)

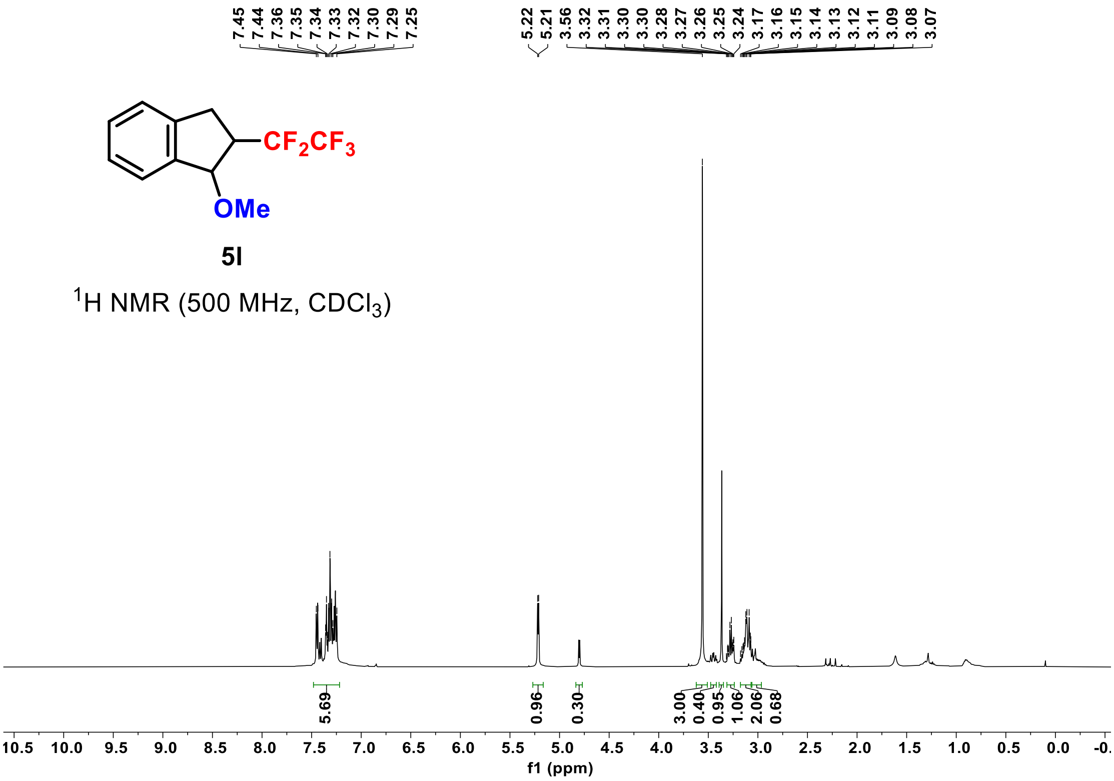
7.45
7.44
7.36
7.35
7.34
7.33
7.32
7.30
7.29
7.25
5.22
5.21
3.56
3.32
3.31
3.30
3.30
3.28
3.27
3.26
3.25
3.24
3.17
3.16
3.15
3.14
3.13
3.12
3.11
3.09
3.08
3.07
CF2CF3
OMe
5l
1H NMR (500 MHz, CDCl3)
5.69
0.96
0.30
3.00
0.40
0.95
1.06
2.06
0.68
f1 (ppm)

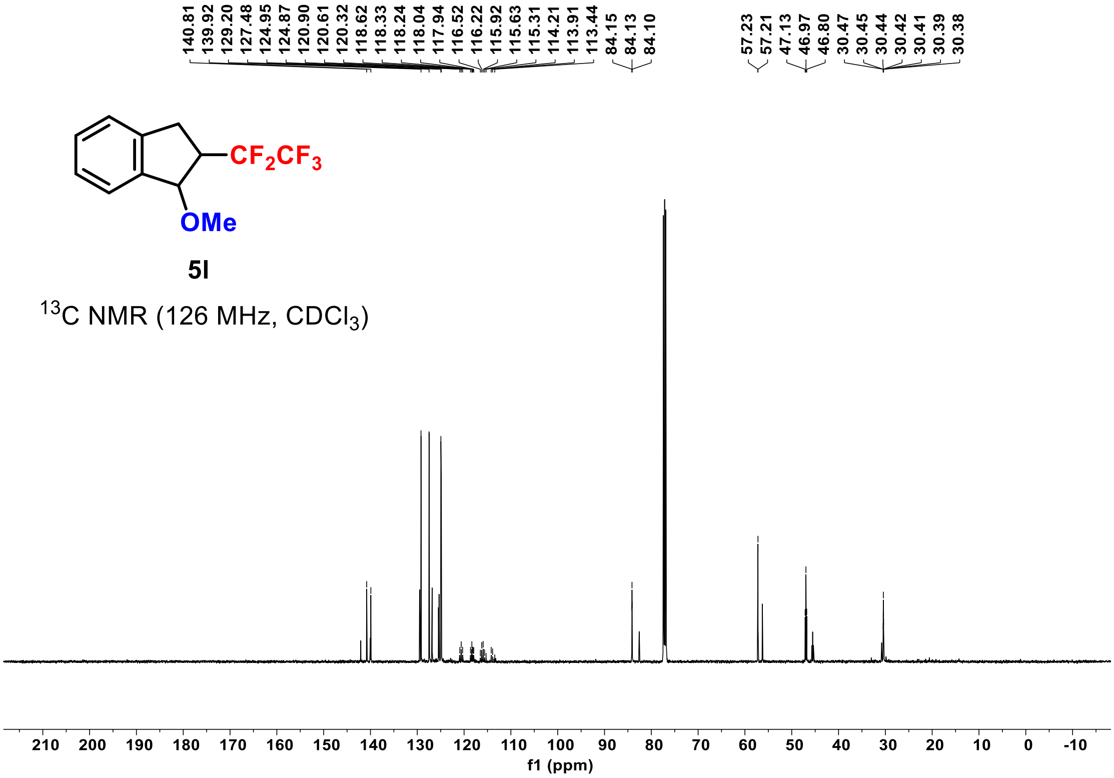
140.81
139.92
129.20
127.48
124.95
124.87
120.90
120.61
120.32
118.62
118.33
118.24
118.04
117.94
116.52
116.22
115.92
115.63
115.31
114.21
113.91
113.44
84.15
84.13
84.10
57.23
57.21
47.13
46.97
46.80
30.47
30.45
30.44
30.42
30.41
30.39
30.38
CF2CF3
OMe
5l
13C NMR (126 MHz, CDCl3)
f1 (ppm)

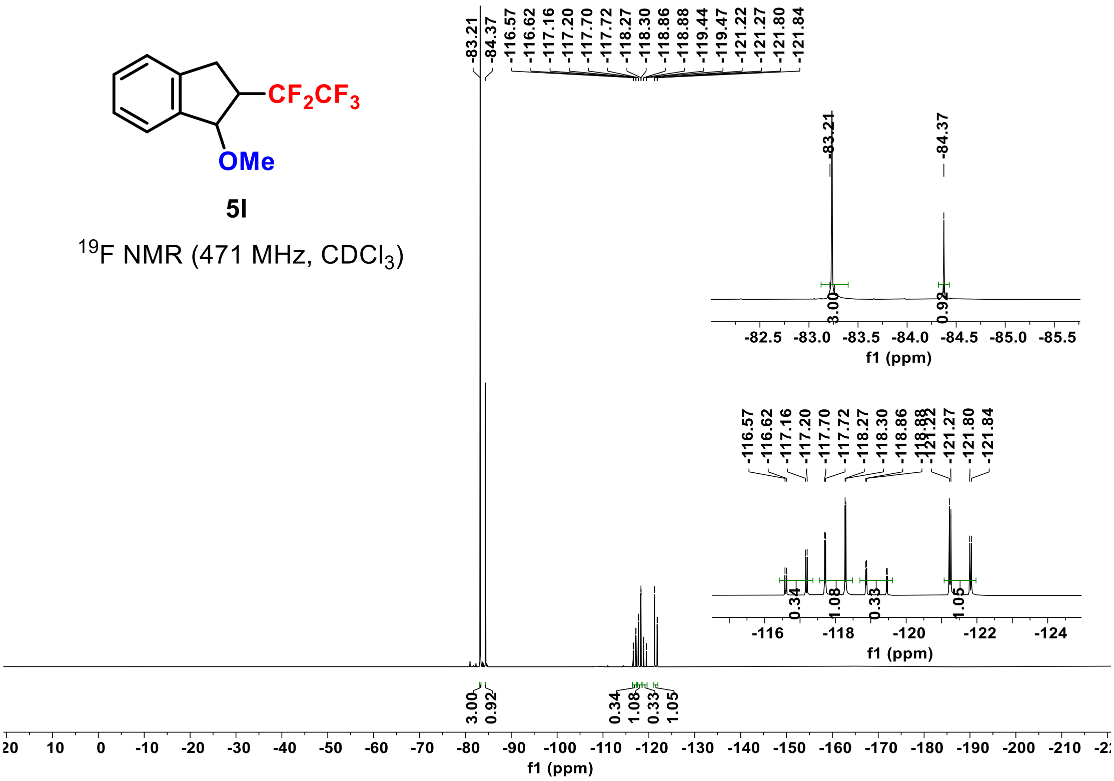

CF2CF3
OMe
5l
19F NMR (471 MHz, CDCl3)
-83.21
-84.37
-116.57
-116.62
-117.16
-117.20
-117.70
-117.72
-118.27
-118.30
-118.86
-118.88
-119.44
-119.47
-121.22
-121.27
-121.80
-121.84
3.00
0.92
0.34
1.08
0.33
1.05
f1 (ppm)

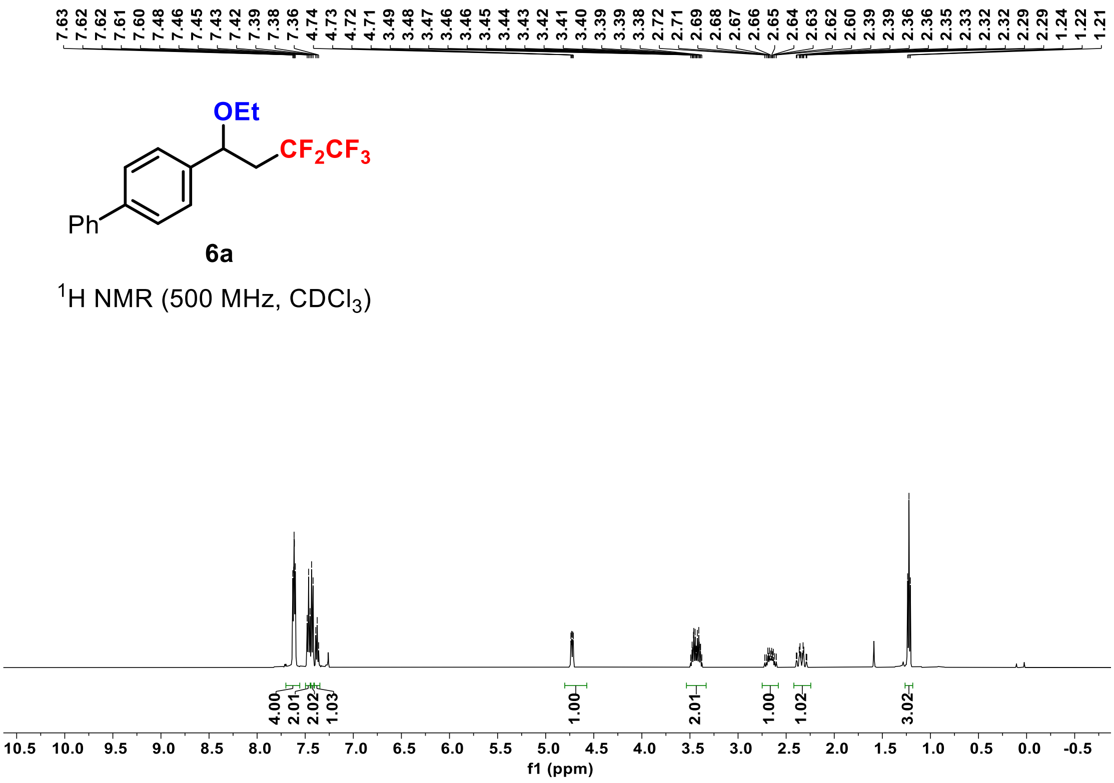

7.63
7.62
7.62
7.61
7.60
7.48
7.46
7.45
7.43
7.42
7.39
7.38
7.36
4.74
4.73
4.72
4.71
3.49
3.48
3.47
3.46
3.46
3.45
3.44
3.43
3.42
3.41
3.40
3.39
3.39
3.38
2.72
2.71
2.69
2.68
2.67
2.66
2.65
2.64
2.63
2.62
2.60
2.39
2.39
2.36
2.36
2.35
2.33
2.32
2.32
2.29
2.29
1.24
1.22
1.21
OEt
CF2CF3
Ph
6a
1H NMR (500 MHz, CDCl3)
4.00
2.01
2.02
1.03
1.00
2.01
1.00
1.02
3.02
f1 (ppm)

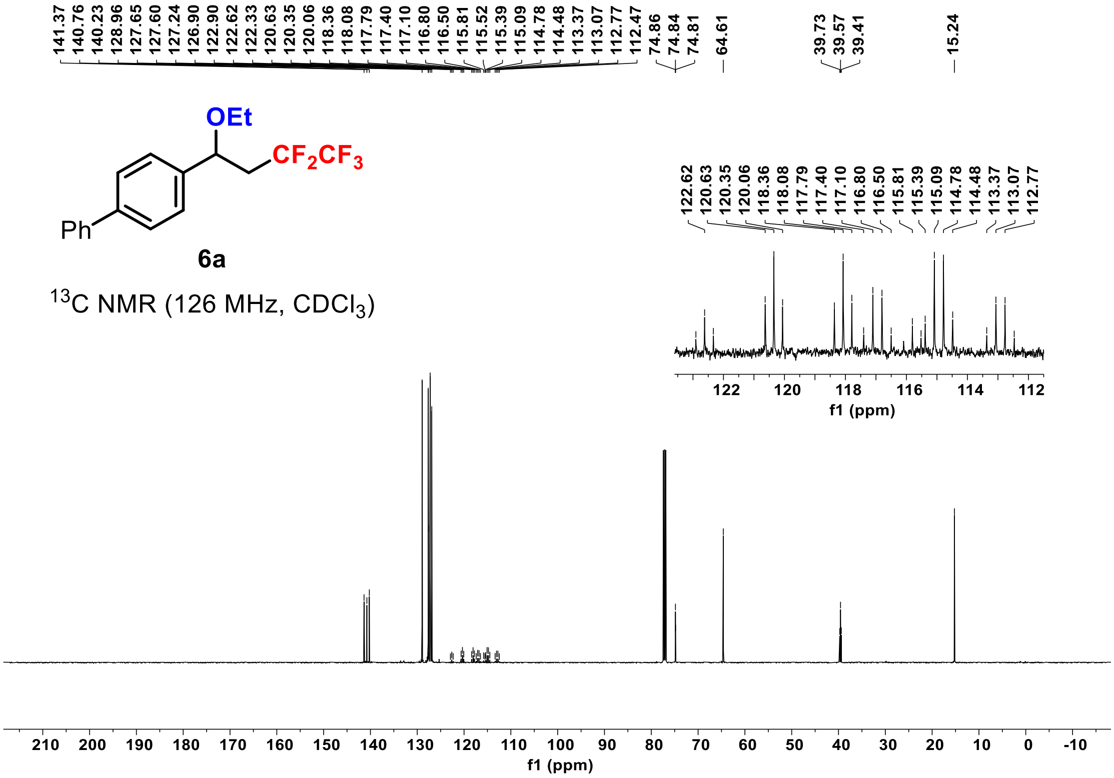
141.37
140.76
140.23
128.96
127.65
127.60
127.24
126.90
122.90
122.62
122.33
120.63
120.35
120.06
118.36
118.08
117.79
117.40
117.10
116.80
116.50
115.81
115.52
115.39
115.09
114.78
114.48
113.37
113.07
112.77
112.47
74.86
74.84
74.81
64.61
39.73
39.57
39.41
15.24
OEt
CF2CF3
Ph
6a
13C NMR (126 MHz, CDCl3)
f1 (ppm)

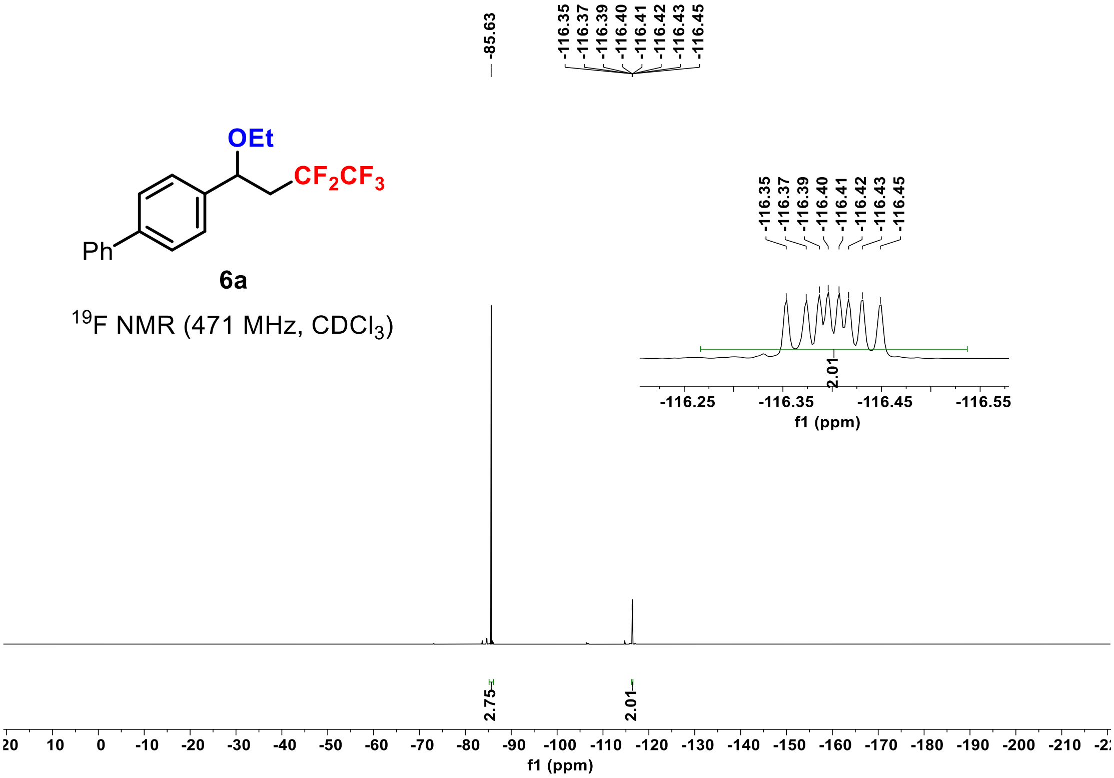
-85.63
-116.35
-116.37
-116.39
-116.40
-116.41
-116.42
-116.43
-116.45
OEt
CF2CF3
Ph
6a
19F NMR (471 MHz, CDCl3)
2.01
2.75
f1 (ppm)

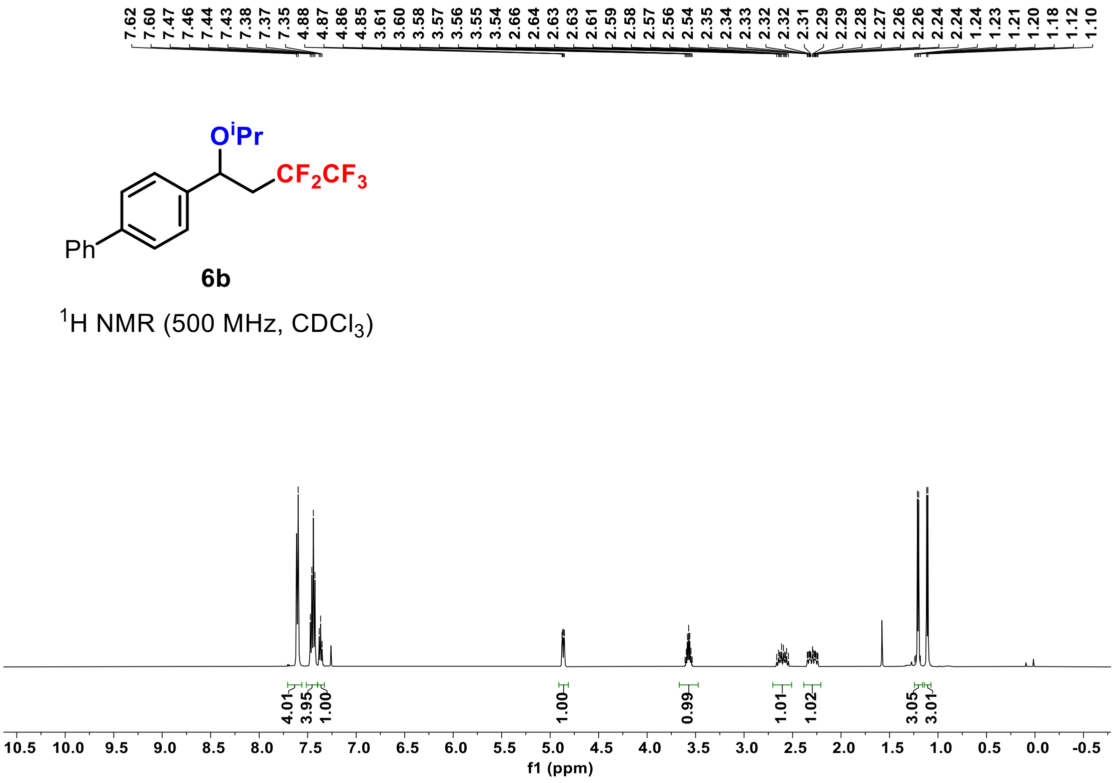

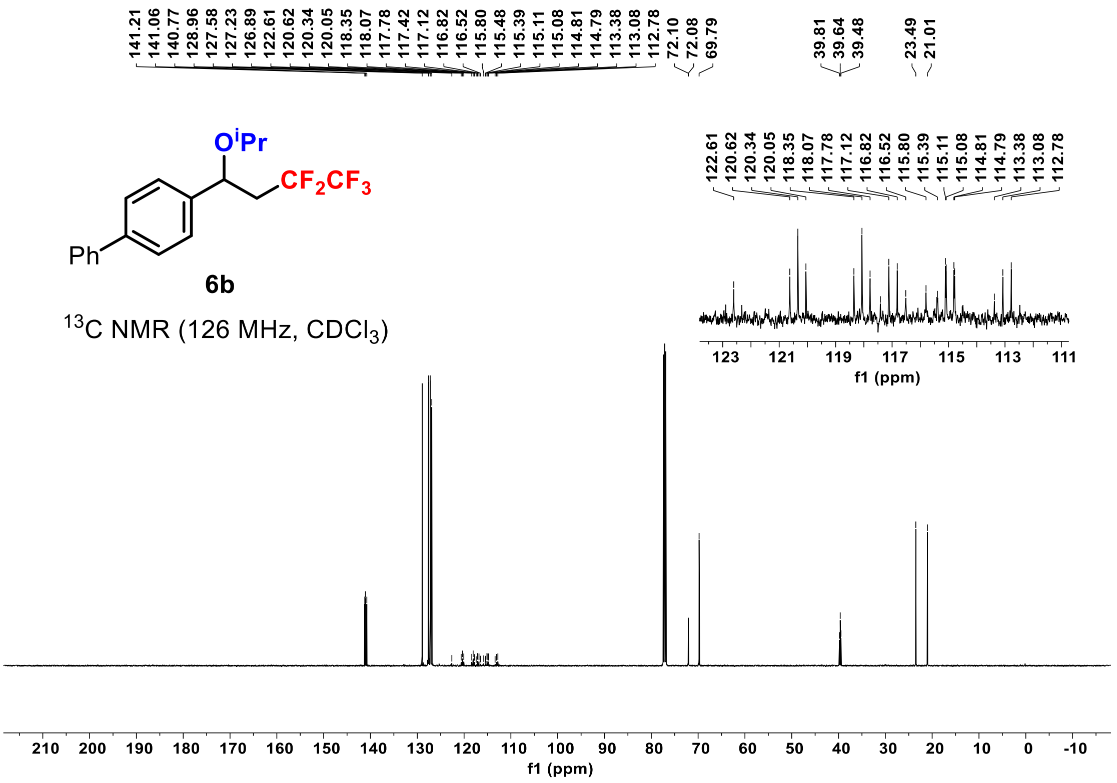

6b

^{19}F NMR (471 MHz, $CDCl_3$)

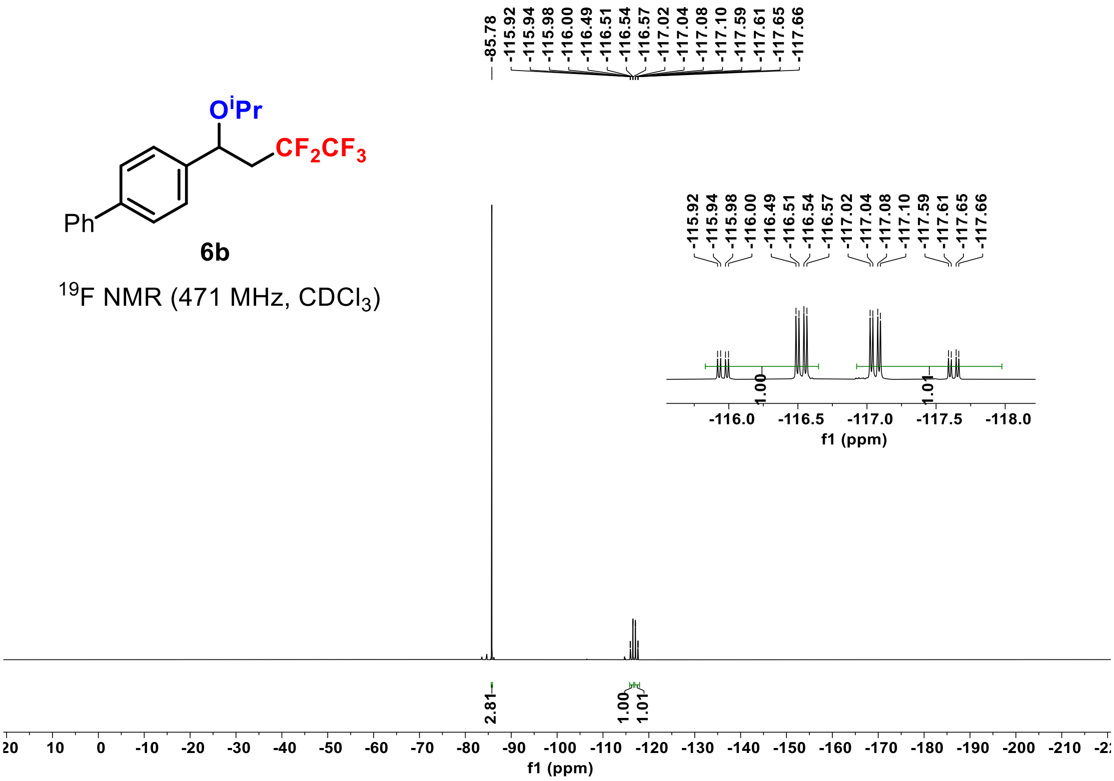

6c

^{1}H NMR (500 MHz, $CDCl_3$)

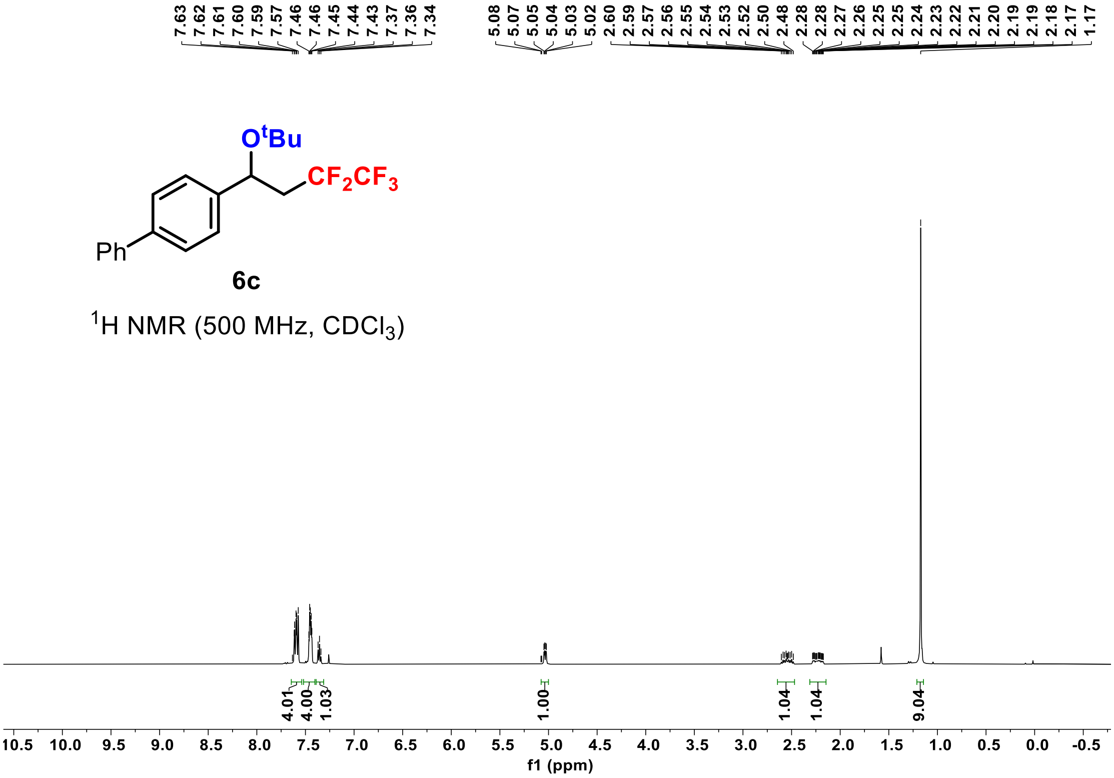

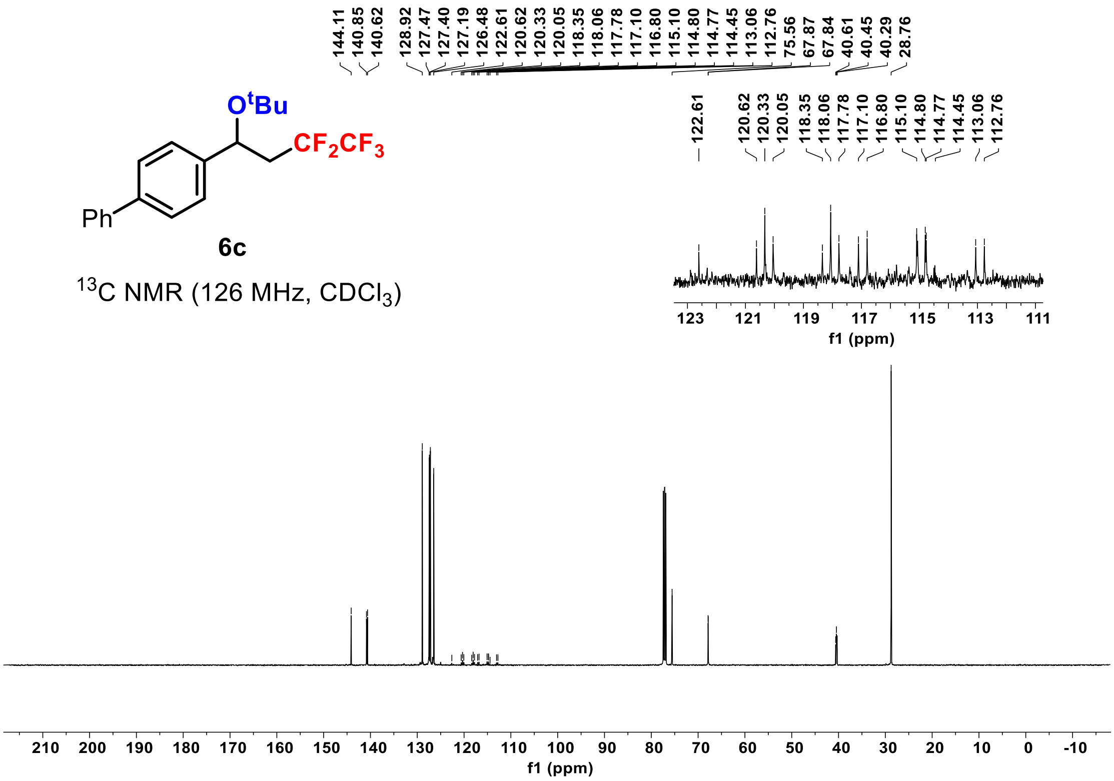
O^tBu
CF2CF3
Ph
6c
13C NMR (126 MHz, CDCl3)
f1 (ppm)

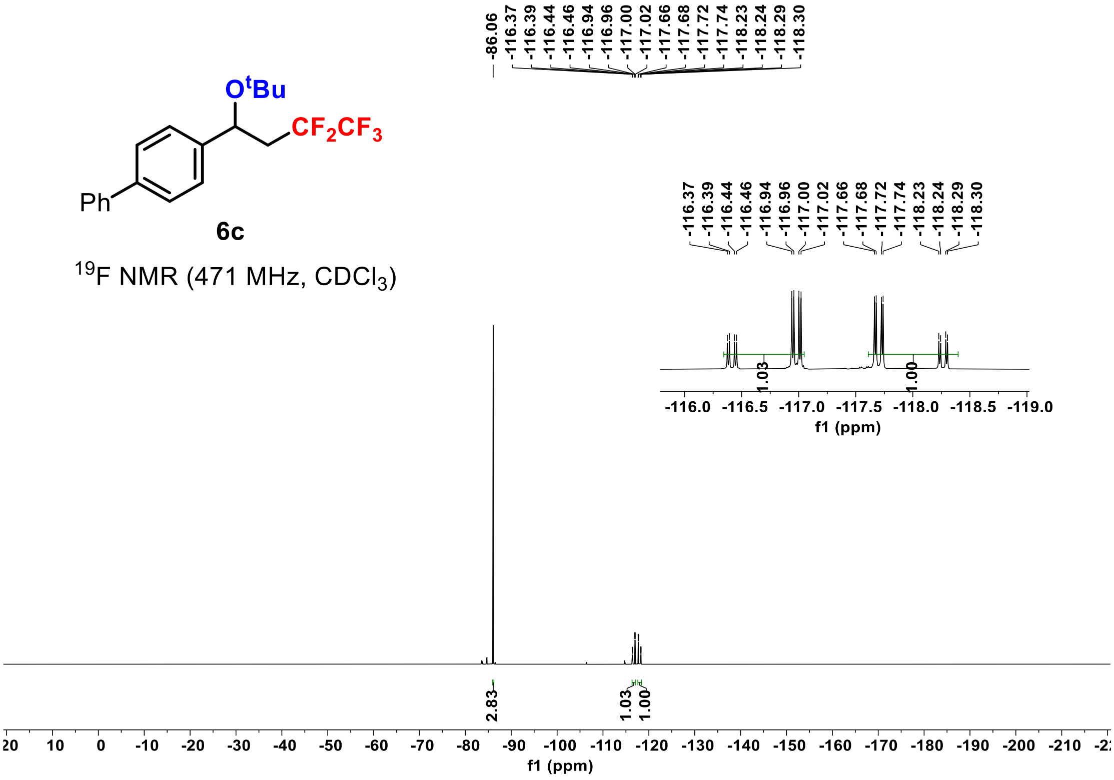
O^tBu
CF2CF3
Ph
6c
19F NMR (471 MHz, CDCl3)
f1 (ppm)

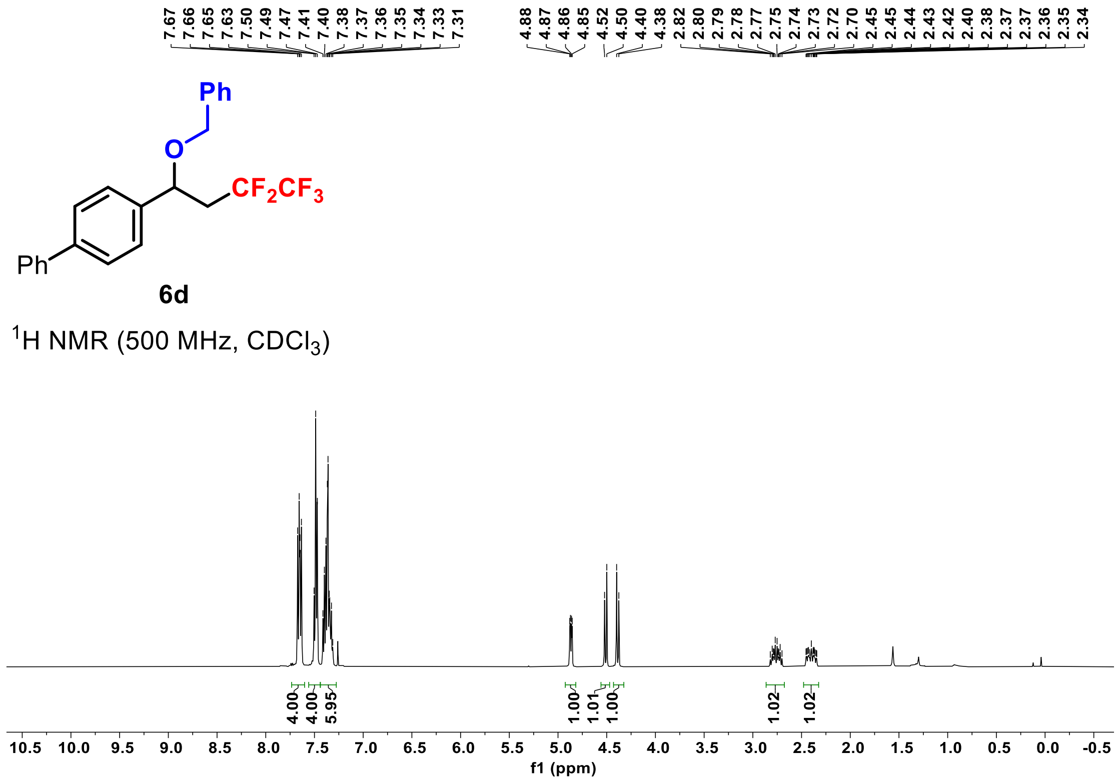
7.67
7.66
7.65
7.63
7.50
7.49
7.47
7.41
7.40
7.38
7.37
7.36
7.35
7.34
7.33
7.31
4.88
4.87
4.86
4.85
4.52
4.50
4.40
4.38
2.82
2.80
2.79
2.78
2.77
2.75
2.74
2.73
2.72
2.70
2.45
2.45
2.44
2.43
2.42
2.40
2.38
2.37
2.37
2.36
2.35
2.34
Ph
O
CF2CF3
Ph
6d
1H NMR (500 MHz, CDCl3)
4.00
4.00
5.95
1.00
1.01
1.00
1.02
1.02
f1 (ppm)

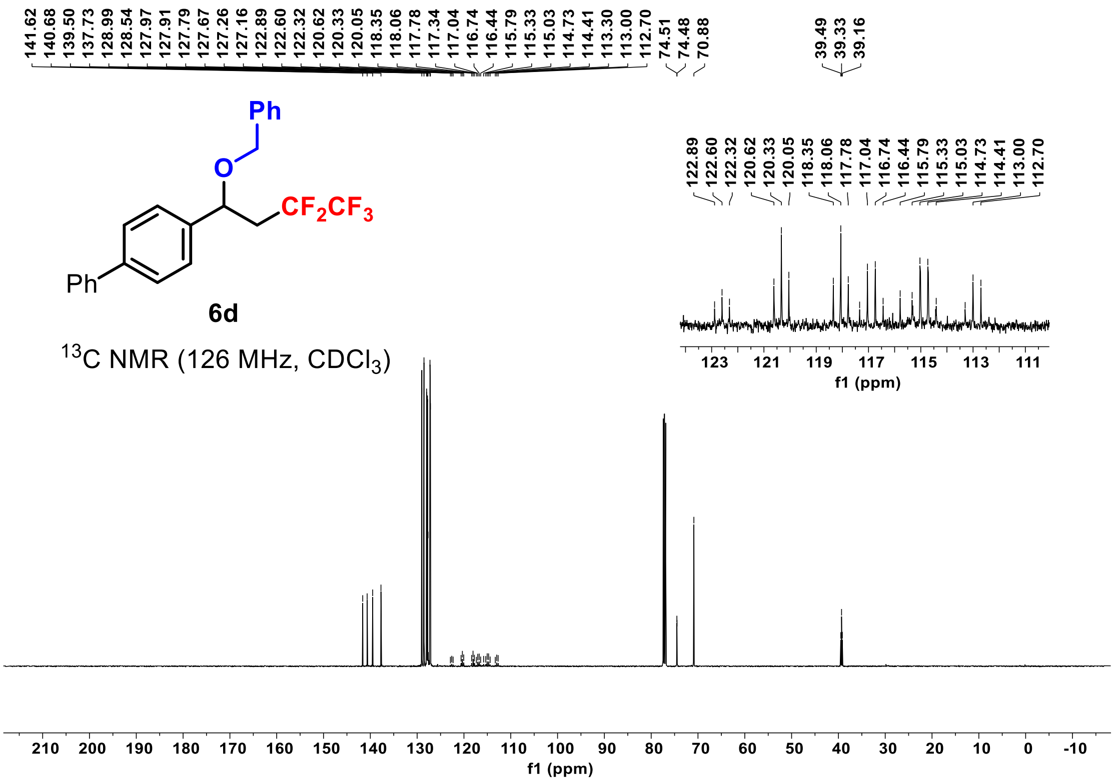
141.62
140.68
139.50
137.73
128.99
128.54
127.97
127.91
127.79
127.67
127.26
127.16
122.89
122.60
122.32
120.62
120.33
120.05
118.35
118.06
117.78
117.34
117.04
116.74
116.44
115.79
115.33
115.03
114.73
114.41
113.30
113.00
112.70
74.51
74.48
70.88
39.49
39.33
39.16
Ph
O
CF2CF3
Ph
6d
13C NMR (126 MHz, CDCl3)
f1 (ppm)

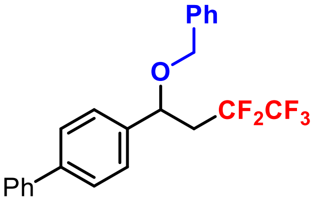

6d

^{19}F NMR (471 MHz, $CDCl_3$)

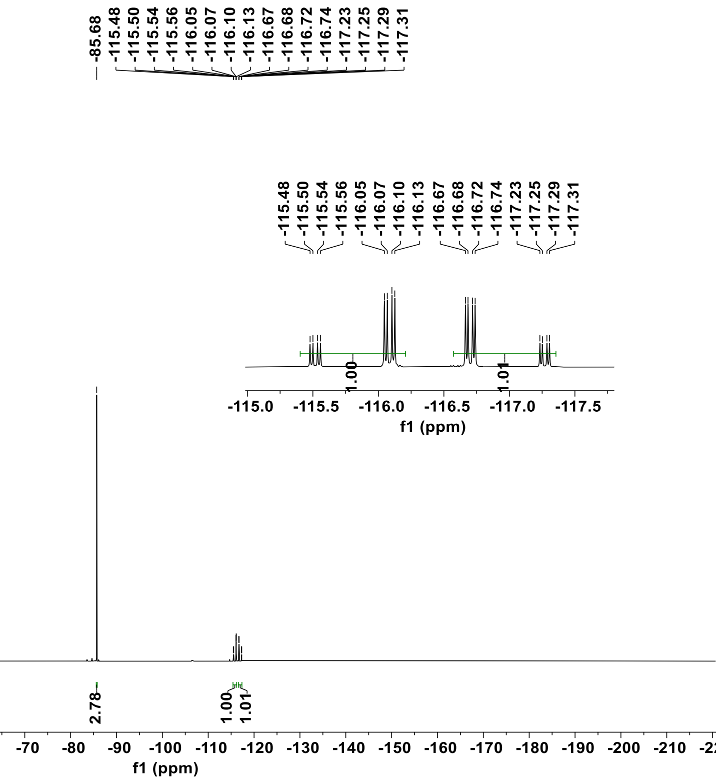

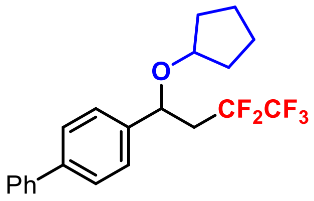

6e

^{1}H NMR (500 MHz, $CDCl_3$)

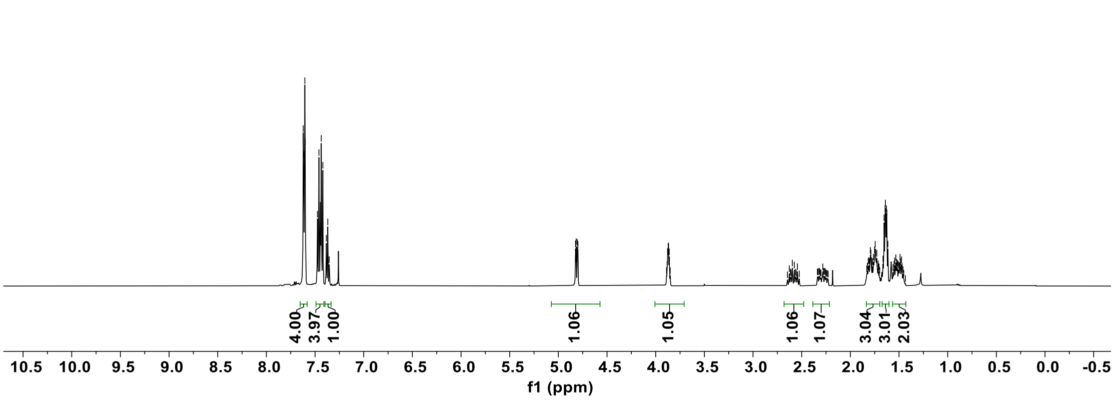

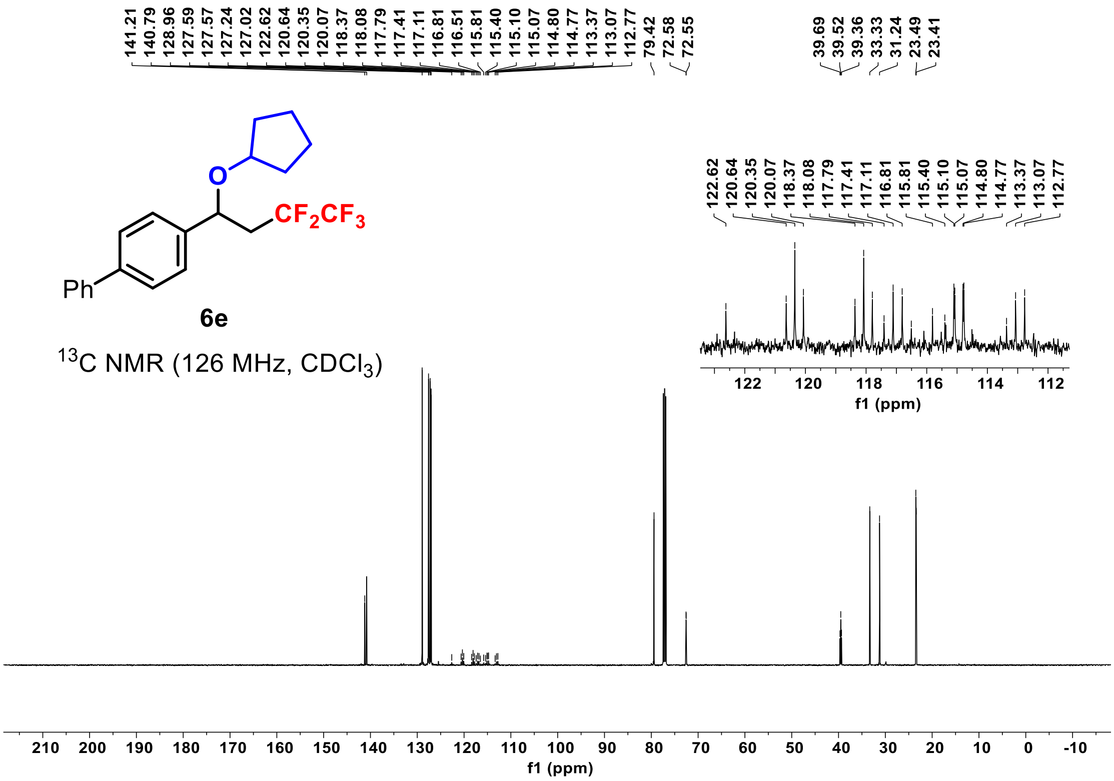
141.21
140.79
128.96
127.59
127.57
127.24
127.02
122.62
120.64
120.35
120.07
118.37
118.08
117.79
117.41
117.11
116.81
116.51
115.81
115.40
115.10
115.07
114.80
114.77
113.37
113.07
112.77
79.42
72.58
72.55
39.69
39.52
39.36
33.33
31.24
23.49
23.41
O
CF2CF3
Ph
6e
13C NMR (126 MHz, CDCl3)
f1 (ppm)

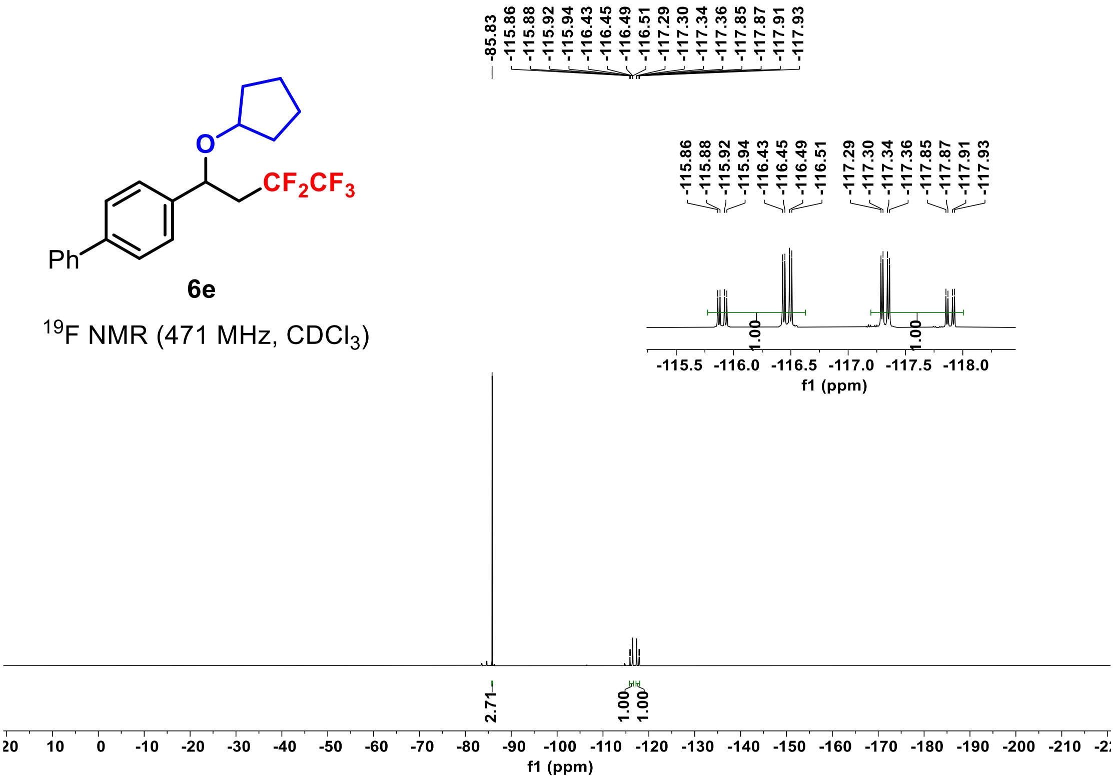
-85.83
-115.86
-115.88
-115.92
-115.94
-116.43
-116.45
-116.49
-116.51
-117.29
-117.30
-117.34
-117.36
-117.85
-117.87
-117.91
-117.93
O
CF2CF3
Ph
6e
19F NMR (471 MHz, CDCl3)
2.71
1.00
1.00
f1 (ppm)

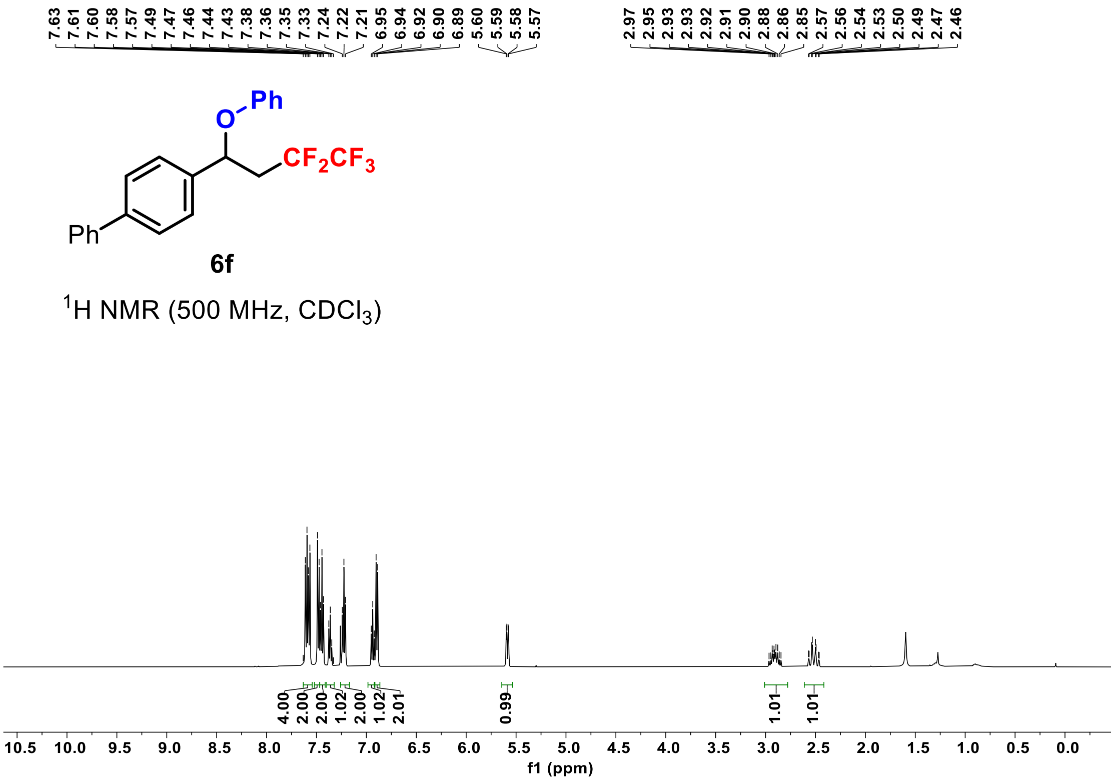

6f

^{1}H NMR (500 MHz, $CDCl_3$)

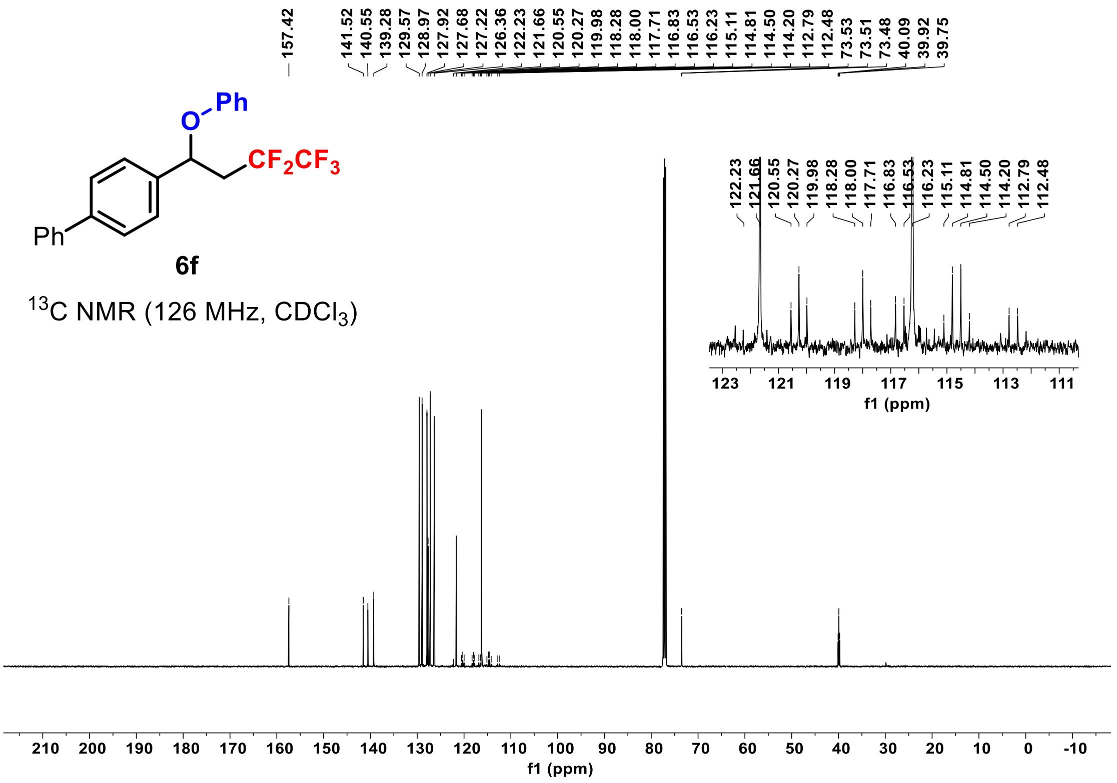

6f

^{13}C NMR (126 MHz, $CDCl_3$)

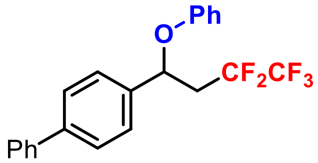

6f

^{19}F NMR (471 MHz, $CDCl_3$)

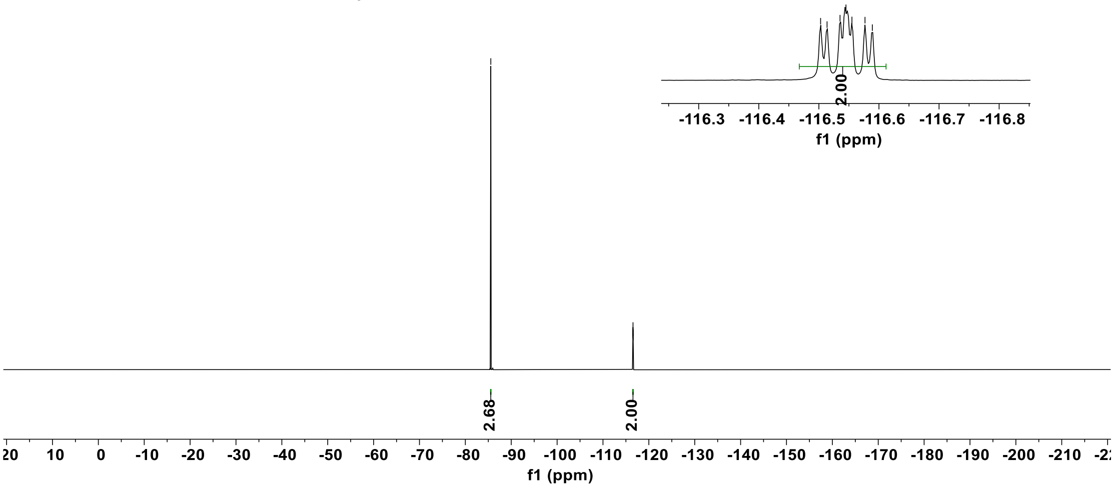

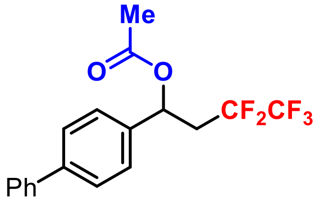

6g

^{1}H NMR (500 MHz, $CDCl_3$)

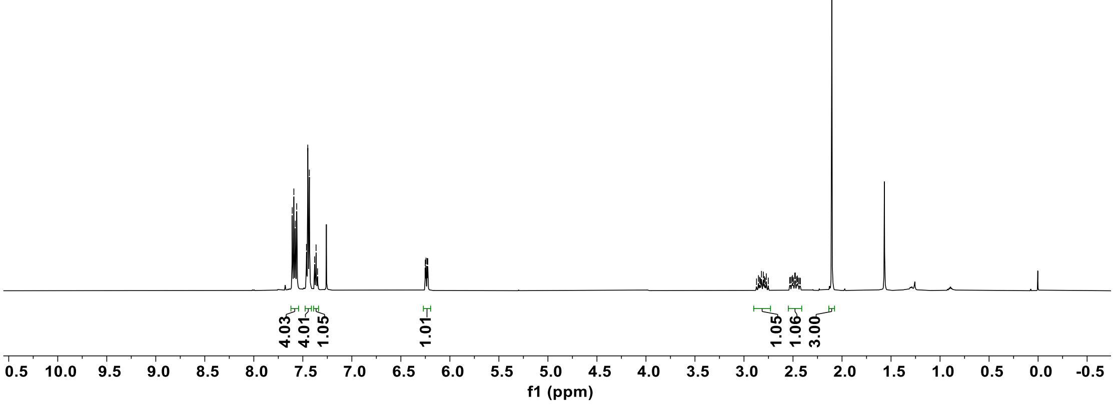

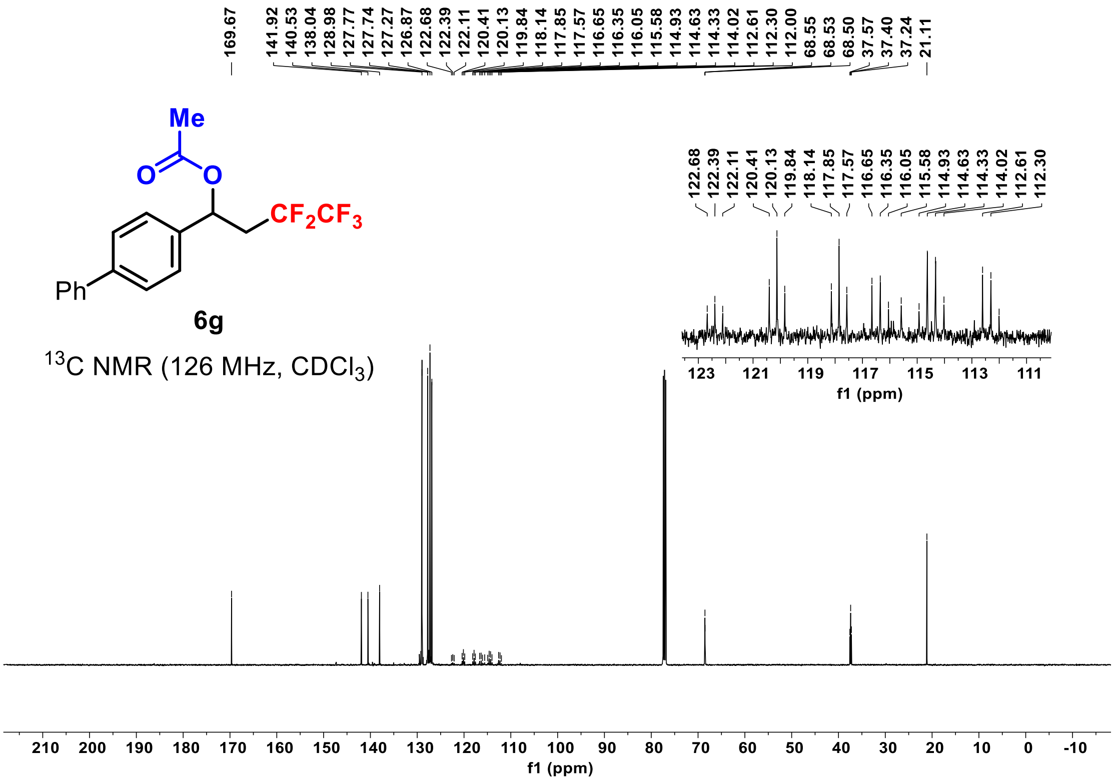
Me
O
O
CF2CF3
Ph
6g
13C NMR (126 MHz, CDCl3)
f1 (ppm)

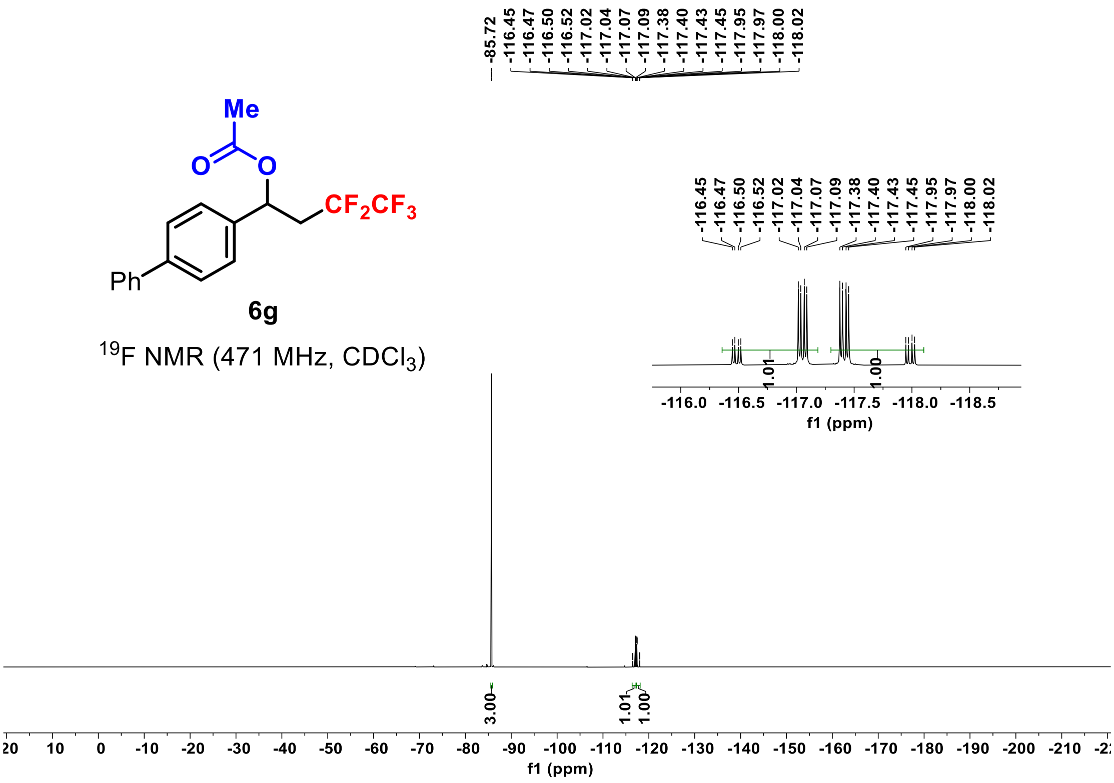
Me
O
O
CF2CF3
Ph
6g
19F NMR (471 MHz, CDCl3)
f1 (ppm)

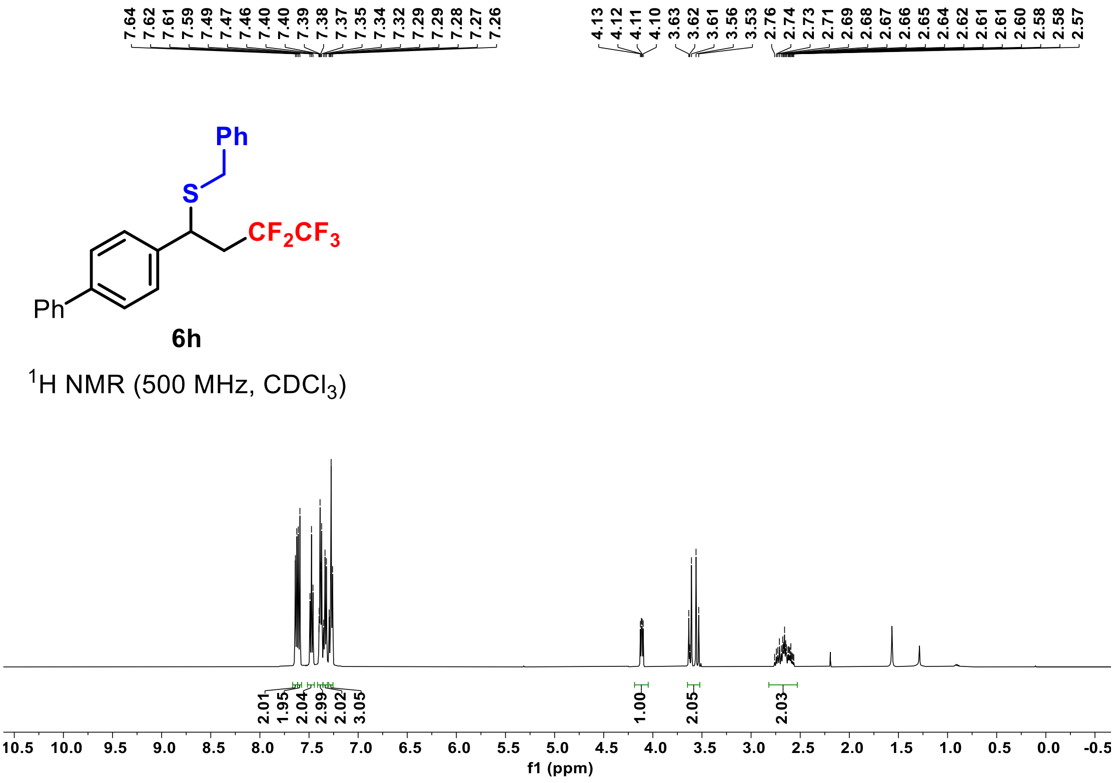

7.64 7.62 7.61 7.59 7.49 7.47 7.46 7.40 7.40 7.39 7.38 7.37 7.35 7.34 7.32 7.29 7.29 7.28 7.27 7.26
4.13 4.12 4.11 4.10 3.63 3.62 3.61 3.56 3.53 2.76 2.74 2.73 2.71 2.69 2.68 2.67 2.66 2.65 2.64 2.62 2.61 2.60 2.58 2.58 2.57
Ph
S
CF2CF3
Ph
6h
1H NMR (500 MHz, CDCl3)
2.01 1.95 2.04 2.99 2.02 3.05
1.00
2.05
2.03
f1 (ppm)

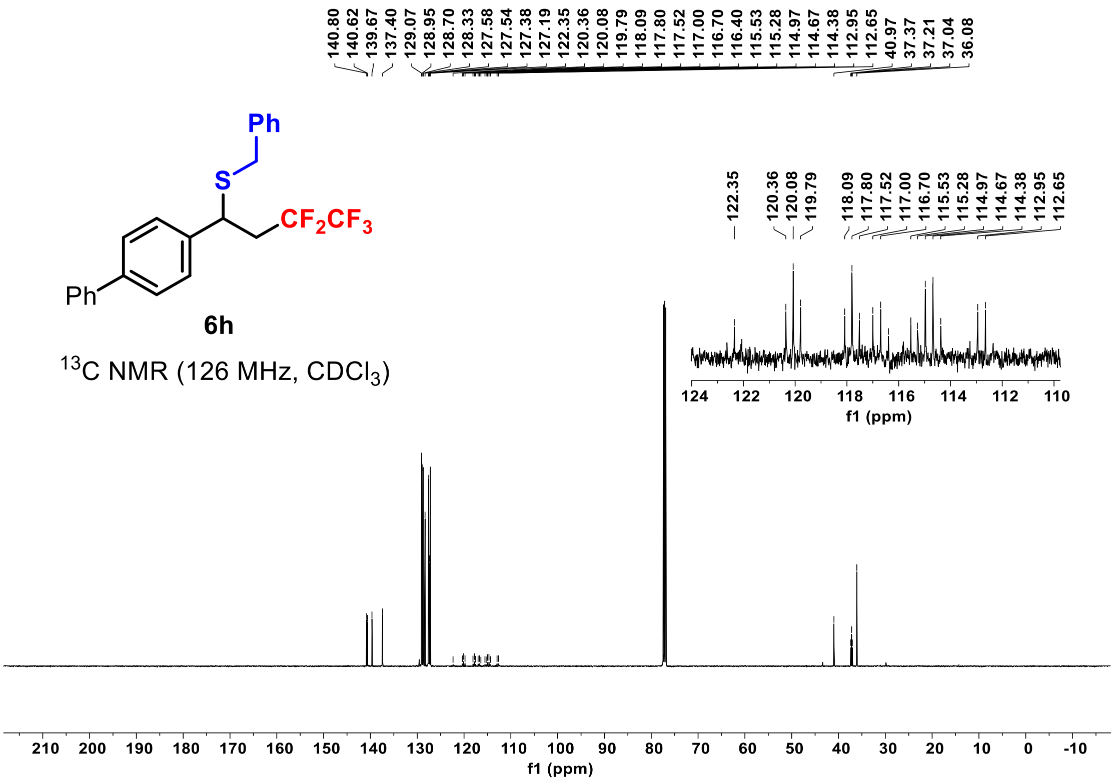

140.80 140.62 139.67 137.40 129.07 128.95 128.70 128.33 127.58 127.54 127.38 127.19 122.35 120.36 120.08 119.79 118.09 117.80 117.52 117.00 116.70 116.40 115.53 115.28 114.97 114.67 114.38 112.95 112.65 40.97 37.37 37.21 37.04 36.08
Ph
S
CF2CF3
Ph
6h
13C NMR (126 MHz, CDCl3)
122.35 120.36 120.08 119.79 118.09 117.80 117.52 117.00 116.70 115.53 115.28 114.97 114.67 114.38 112.95 112.65
f1 (ppm)
f1 (ppm)

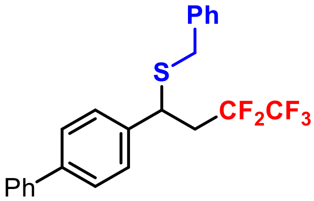

6h

^{19}F NMR (471 MHz, $CDCl_3$)

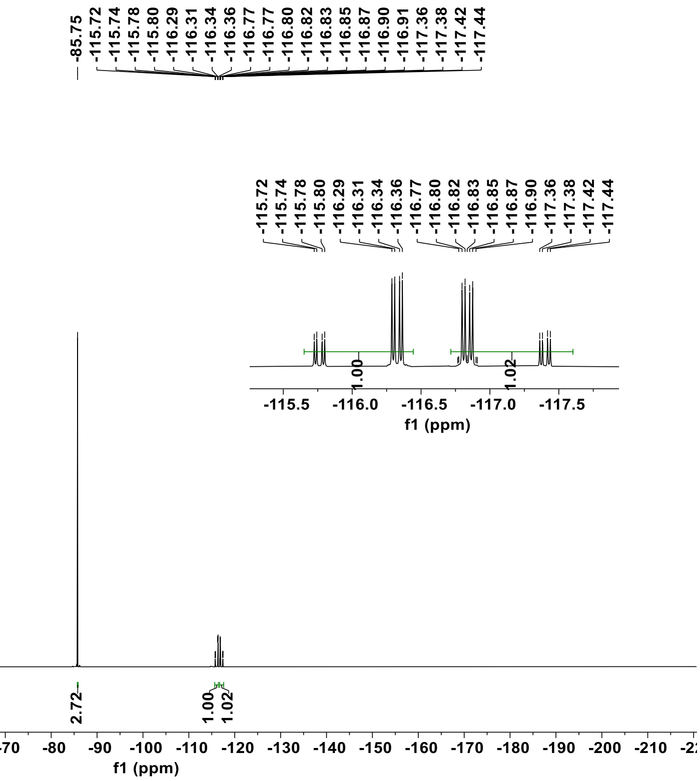

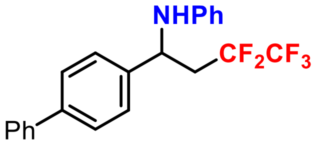

6i

^{1}H NMR (500 MHz, $CDCl_3$)

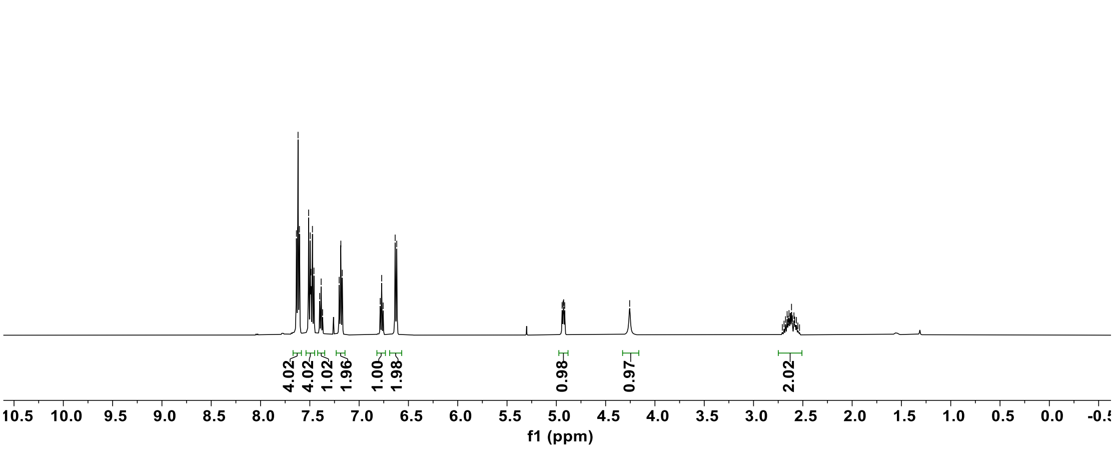

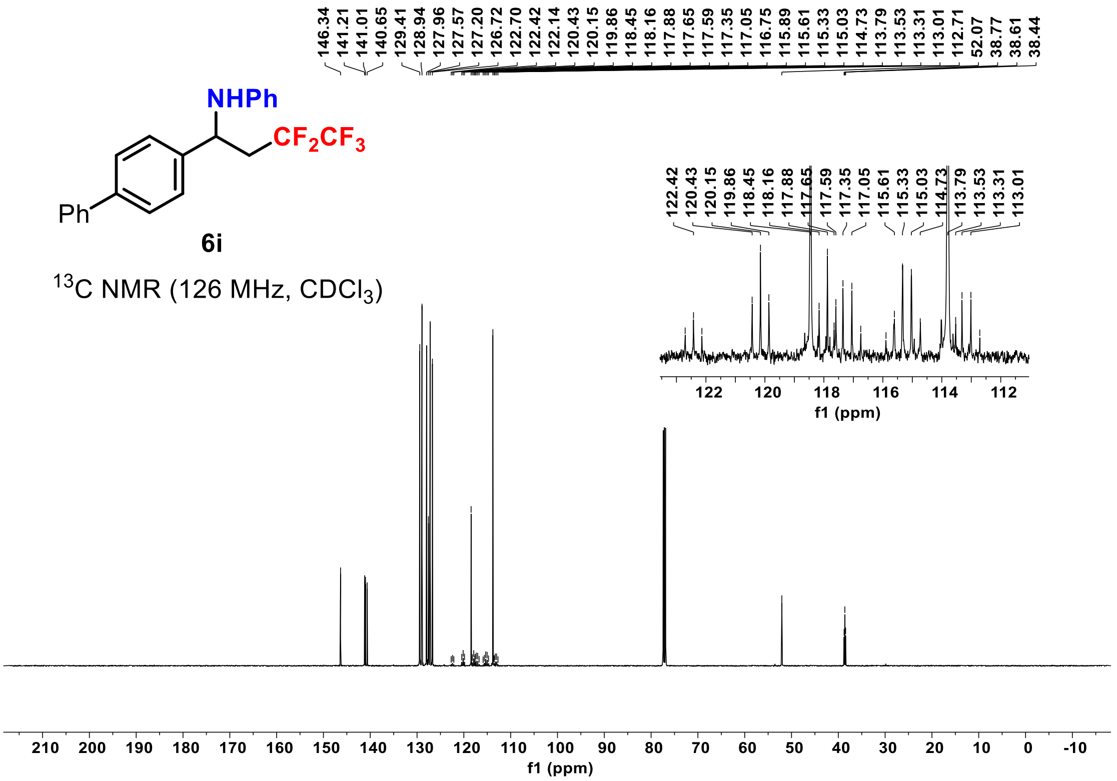

6i

^{13}C NMR (126 MHz, $CDCl_3$)

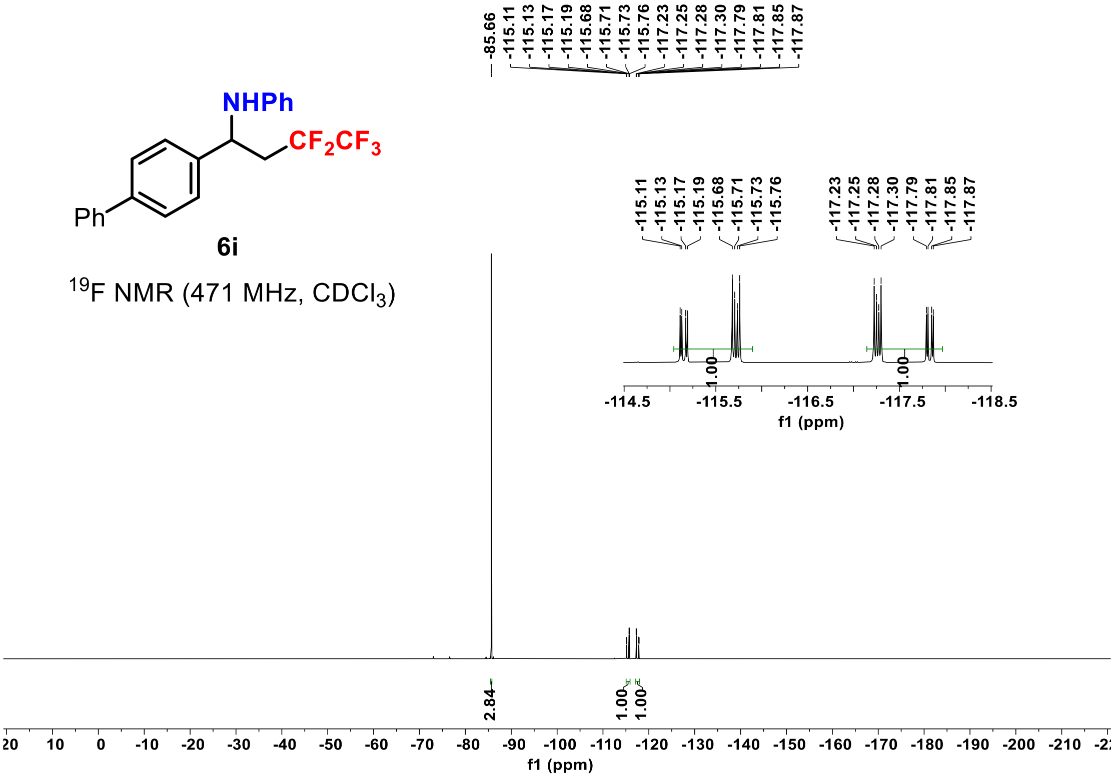

6i

^{19}F NMR (471 MHz, $CDCl_3$)

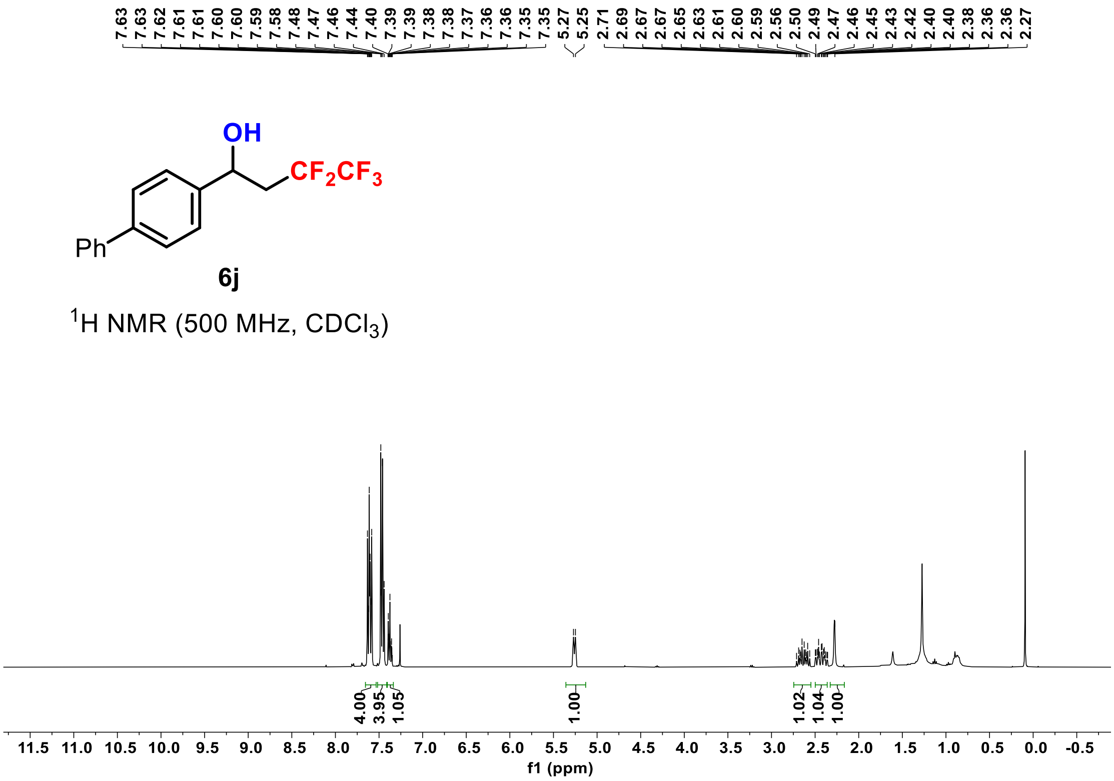

6j

^1H NMR (500 MHz, $CDCl_3$)

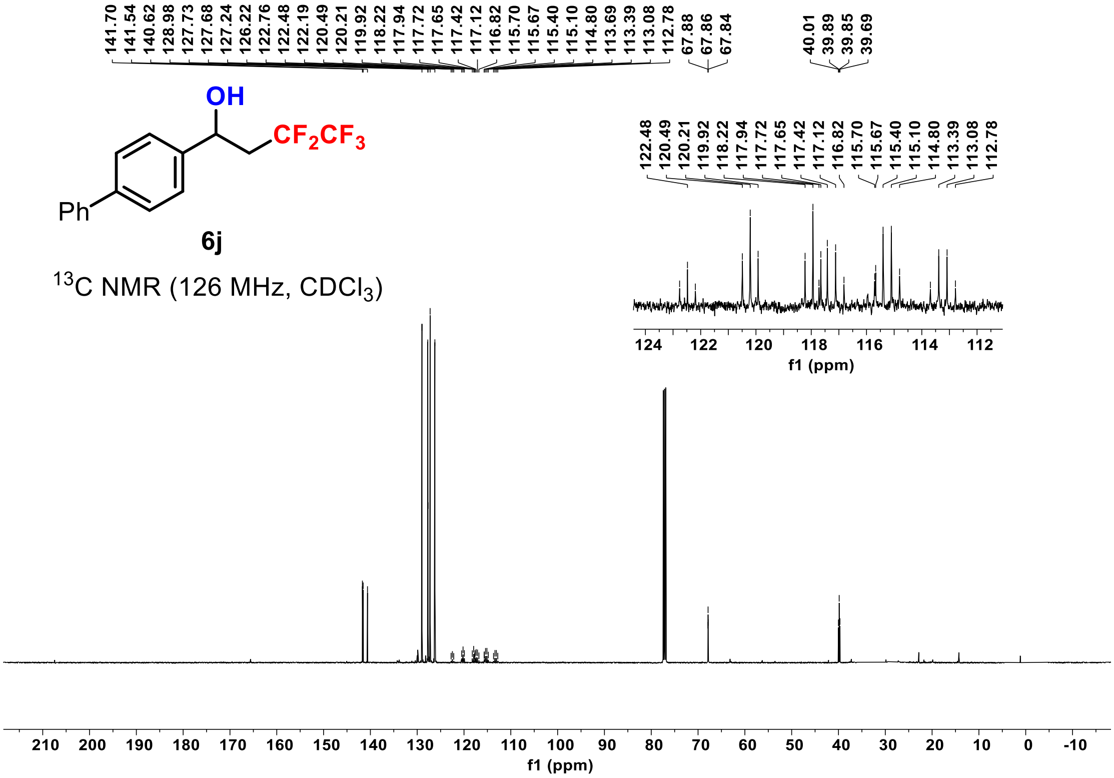

6j

^{13}C NMR (126 MHz, $CDCl_3$)

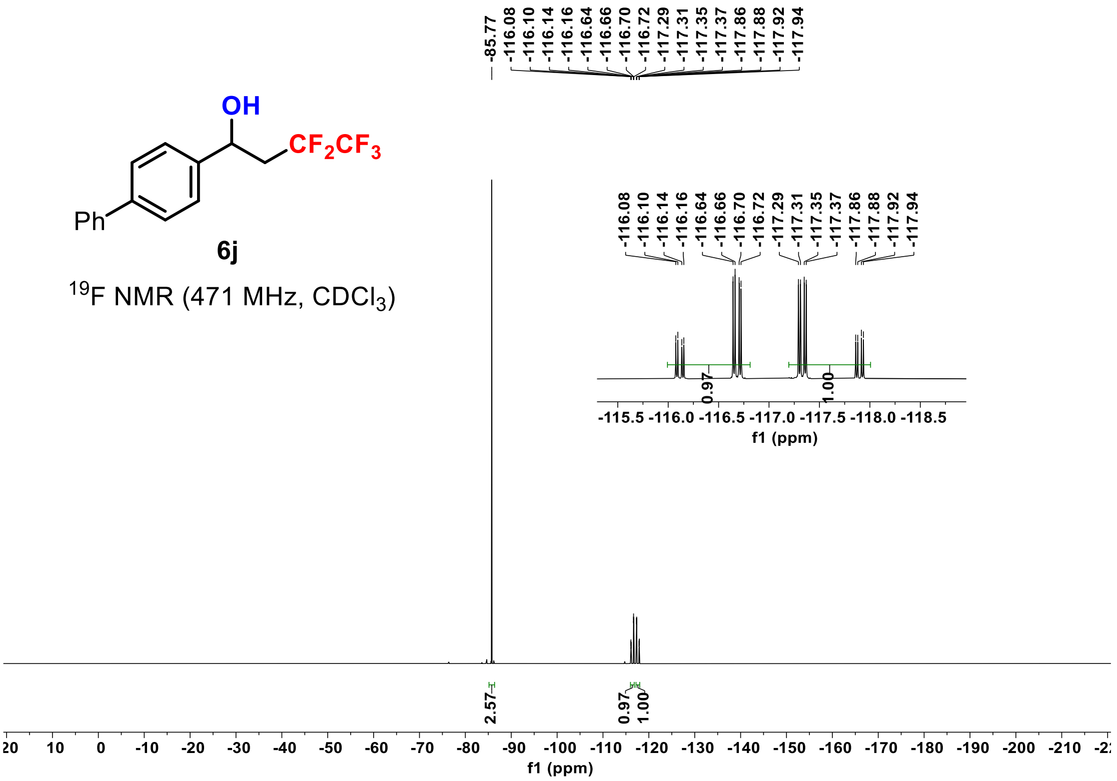
OH
CF2CF3
Ph
6j
19F NMR (471 MHz, CDCl3)
-85.77
-116.08
-116.10
-116.14
-116.16
-116.64
-116.66
-116.70
-116.72
-117.29
-117.31
-117.35
-117.37
-117.86
-117.88
-117.92
-117.94
0.97
1.00
2.57
-115.5 -116.0 -116.5 -117.0 -117.5 -118.0 -118.5
f1 (ppm)

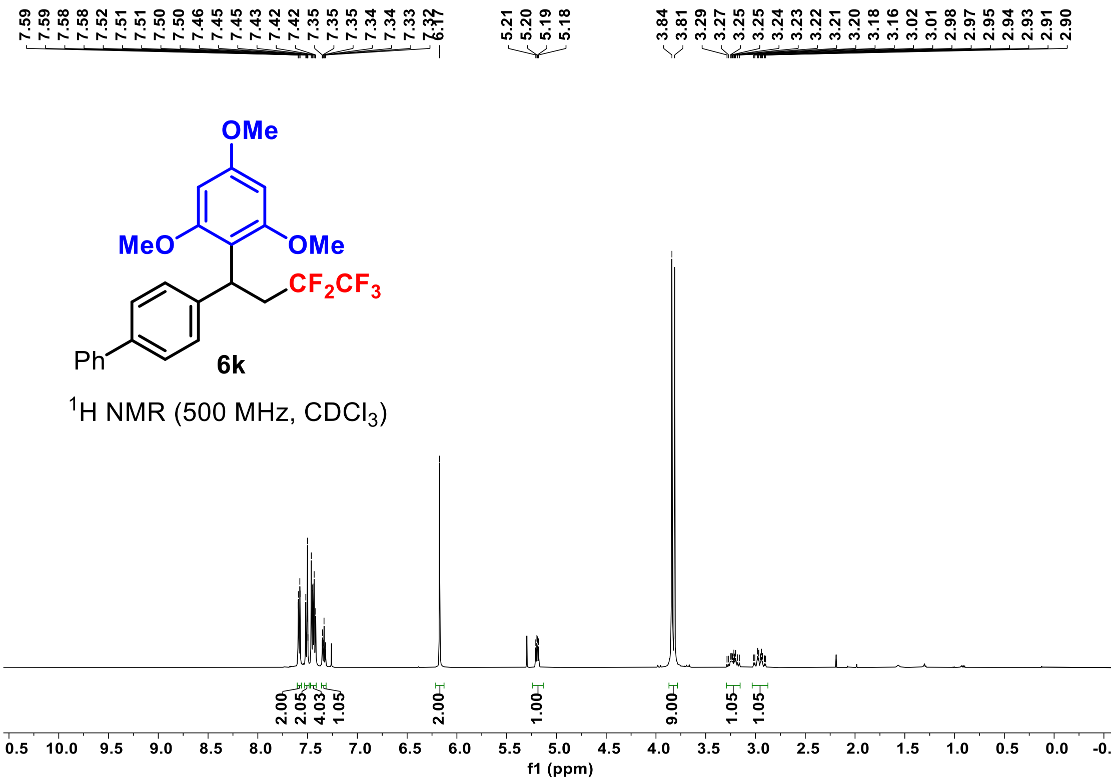
OMe
MeO
OMe
CF2CF3
Ph
6k
1H NMR (500 MHz, CDCl3)
7.59
7.59
7.58
7.58
7.52
7.51
7.51
7.50
7.50
7.46
7.45
7.45
7.43
7.42
7.42
7.35
7.35
7.35
7.34
7.34
7.33
7.32
6.17
5.21
5.20
5.19
5.18
3.84
3.81
3.29
3.27
3.25
3.25
3.24
3.23
3.22
3.21
3.20
3.18
3.16
3.02
3.01
2.98
2.97
2.95
2.94
2.93
2.91
2.90
2.00
2.05
4.03
1.05
2.00
1.00
9.00
1.05
1.05
f1 (ppm)

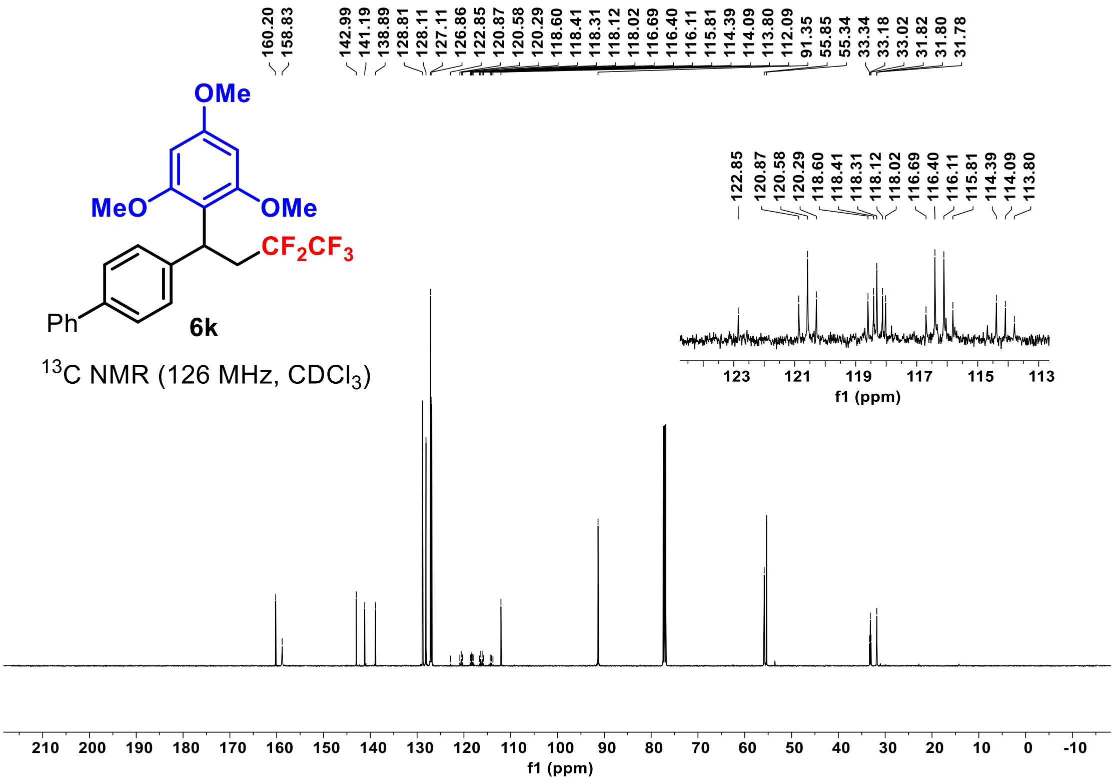

OMe
MeO
OMe
CF2CF3
Ph
6k
13C NMR (126 MHz, CDCl3)
160.20
158.83
142.99
141.19
138.89
128.81
128.11
127.11
126.86
122.85
120.87
120.58
120.29
118.60
118.41
118.31
118.12
118.02
116.69
116.40
116.11
115.81
114.39
114.09
113.80
112.09
91.35
55.85
55.34
33.34
33.18
33.02
31.82
31.80
31.78
f1 (ppm)

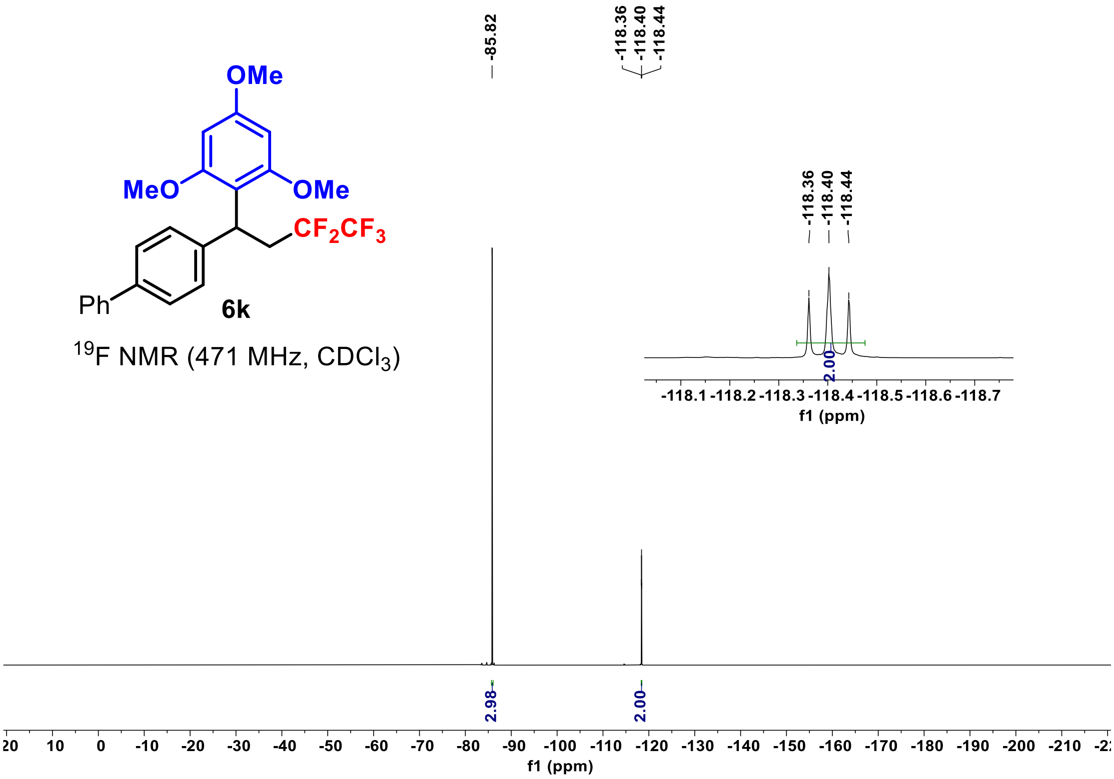

OMe
MeO
OMe
CF2CF3
Ph
6k
19F NMR (471 MHz, CDCl3)
-85.82
-118.36
-118.40
-118.44
2.00
2.98
f1 (ppm)

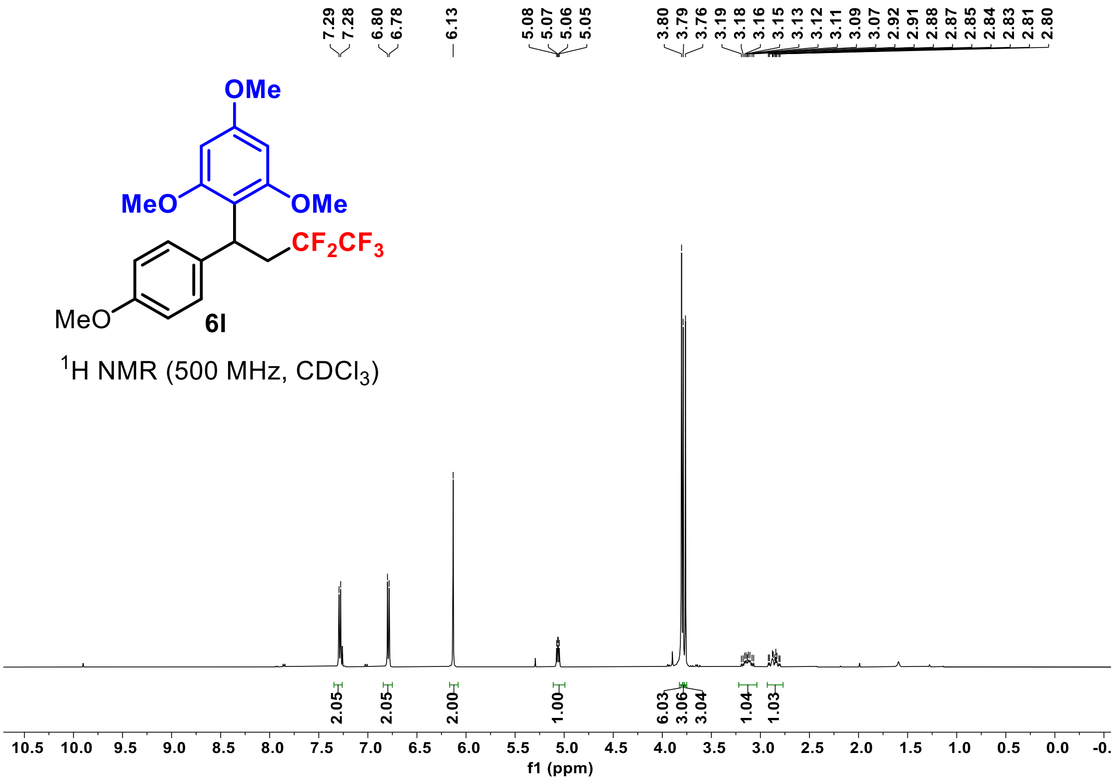
7.29
7.28
6.80
6.78
6.13
5.08
5.07
5.06
5.05
3.80
3.79
3.76
3.19
3.18
3.16
3.15
3.13
3.12
3.11
3.09
3.07
2.92
2.91
2.88
2.87
2.85
2.84
2.83
2.81
2.80
OMe
MeO
OMe
CF2CF3
MeO
6l
1H NMR (500 MHz, CDCl3)
2.05
2.05
2.00
1.00
6.03
3.06
3.04
1.04
1.03
10.5
10.0
9.5
9.0
8.5
8.0
7.5
7.0
6.5
6.0
5.5
5.0
4.5
4.0
3.5
3.0
2.5
2.0
1.5
1.0
0.5
0.0
f1 (ppm)

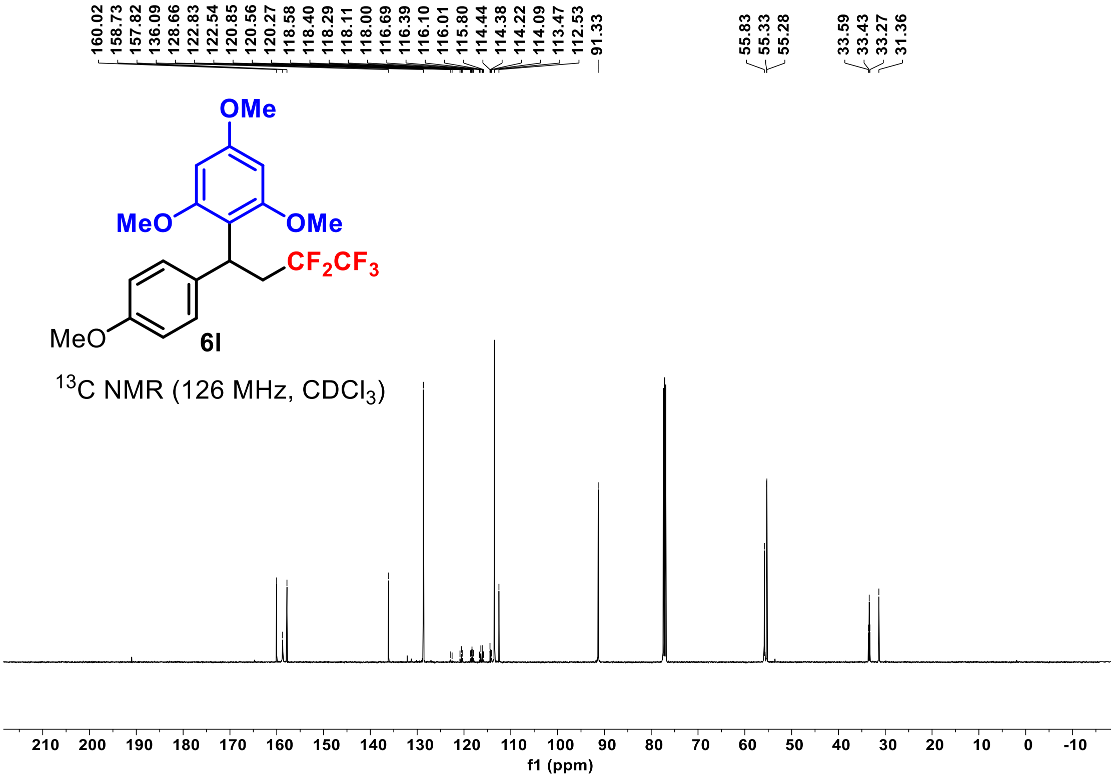
160.02
158.73
157.82
136.09
128.66
122.83
122.54
120.85
120.56
120.27
118.58
118.40
118.29
118.11
118.00
116.69
116.39
116.10
116.01
115.80
114.44
114.38
114.22
114.09
113.47
112.53
91.33
55.83
55.33
55.28
33.59
33.43
33.27
31.36
OMe
MeO
OMe
CF2CF3
MeO
6l
13C NMR (126 MHz, CDCl3)
210
200
190
180
170
160
150
140
130
120
110
100
90
80
70
60
50
40
30
20
10
0
-10
f1 (ppm)

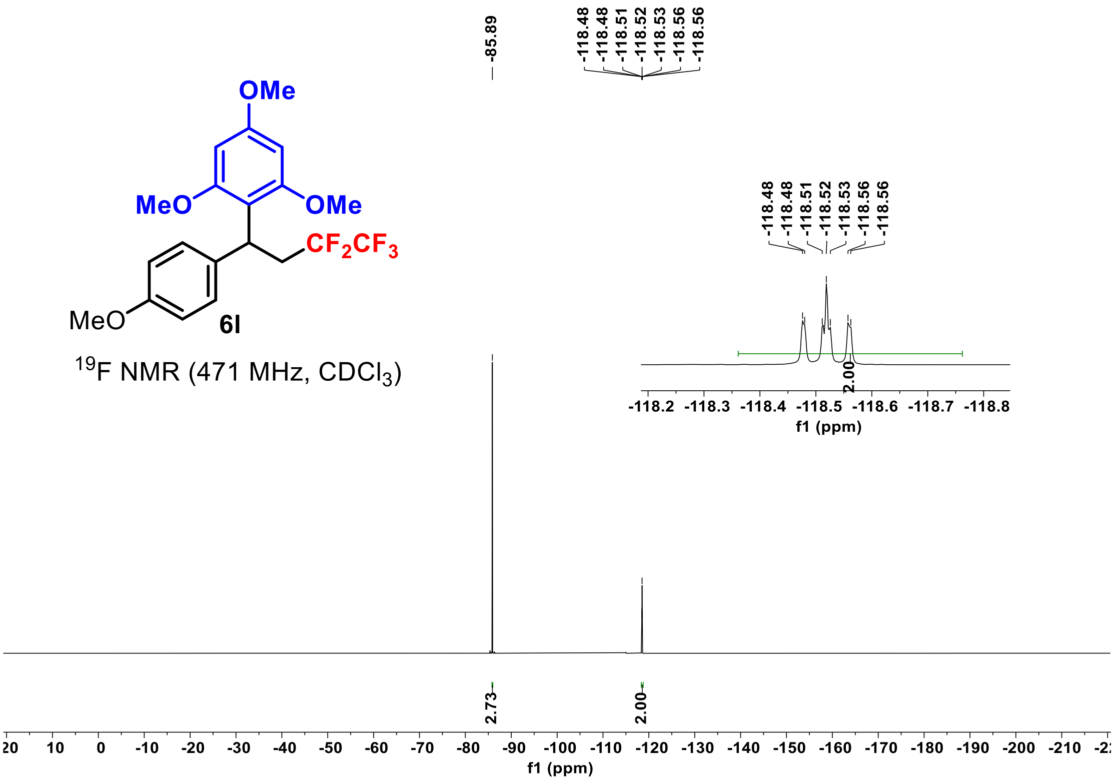

-85.89
-118.48
-118.48
-118.51
-118.52
-118.53
-118.56
-118.56
OMe
MeO
OMe
CF2CF3
MeO
6l
19F NMR (471 MHz, CDCl3)
2.00
f1 (ppm)
2.73
2.00
f1 (ppm)

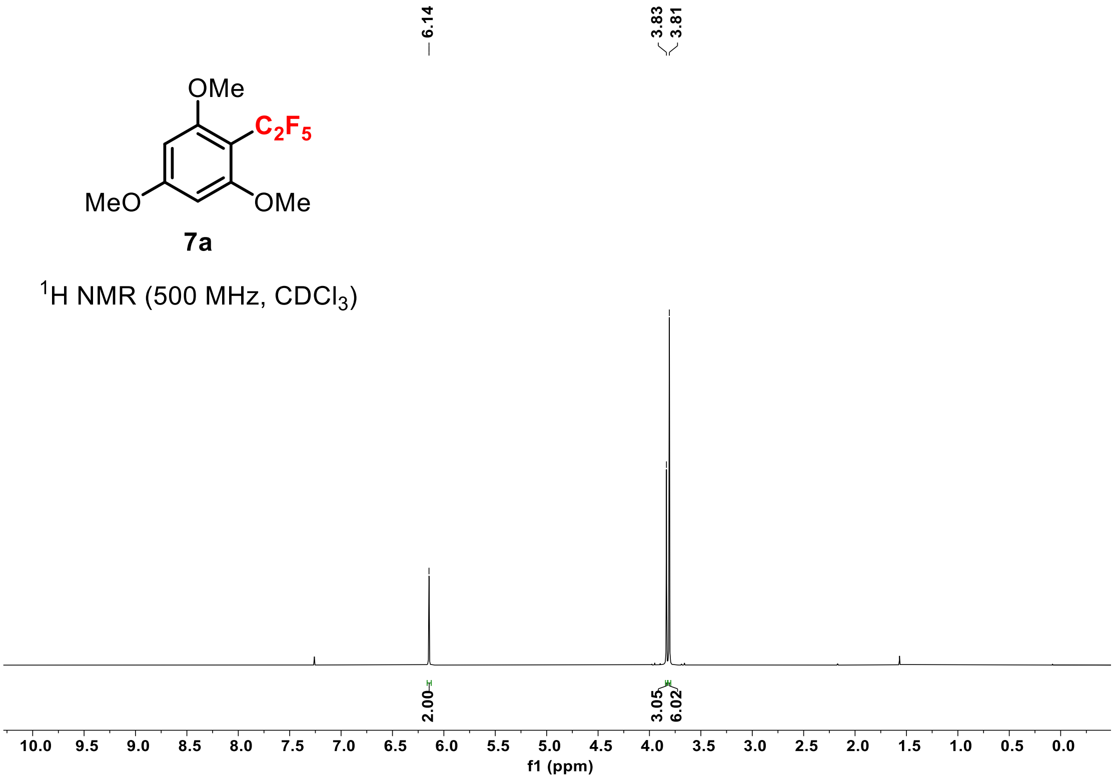

6.14
3.83
3.81
OMe
C2F5
MeO
OMe
7a
1H NMR (500 MHz, CDCl3)
2.00
3.05
6.02
f1 (ppm)

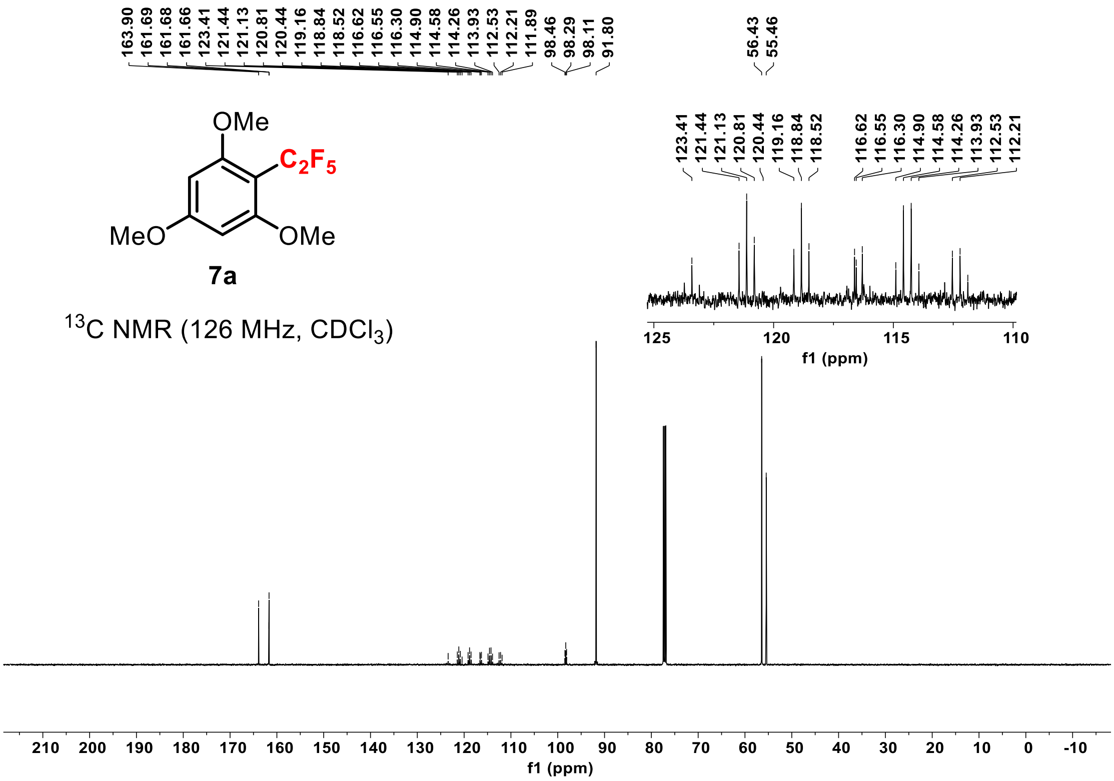
163.90
161.69
161.68
161.66
123.41
121.44
121.13
120.81
120.44
119.16
118.84
118.52
116.62
116.55
116.30
114.90
114.58
114.26
113.93
112.53
112.21
111.89
98.46
98.29
98.11
91.80
56.43
55.46
OMe
C2F5
MeO
OMe
7a
13C NMR (126 MHz, CDCl3)
f1 (ppm)

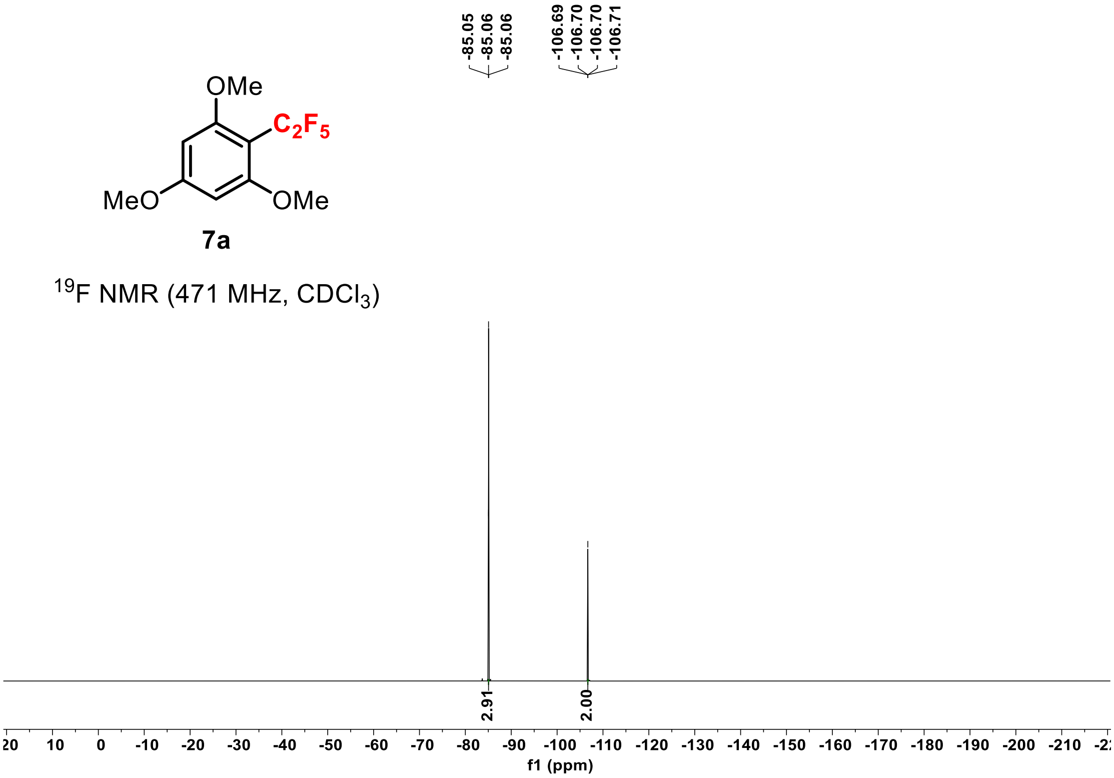
-85.05
-85.06
-85.06
-106.69
-106.70
-106.70
-106.71
OMe
C2F5
MeO
OMe
7a
19F NMR (471 MHz, CDCl3)
2.91
2.00
f1 (ppm)

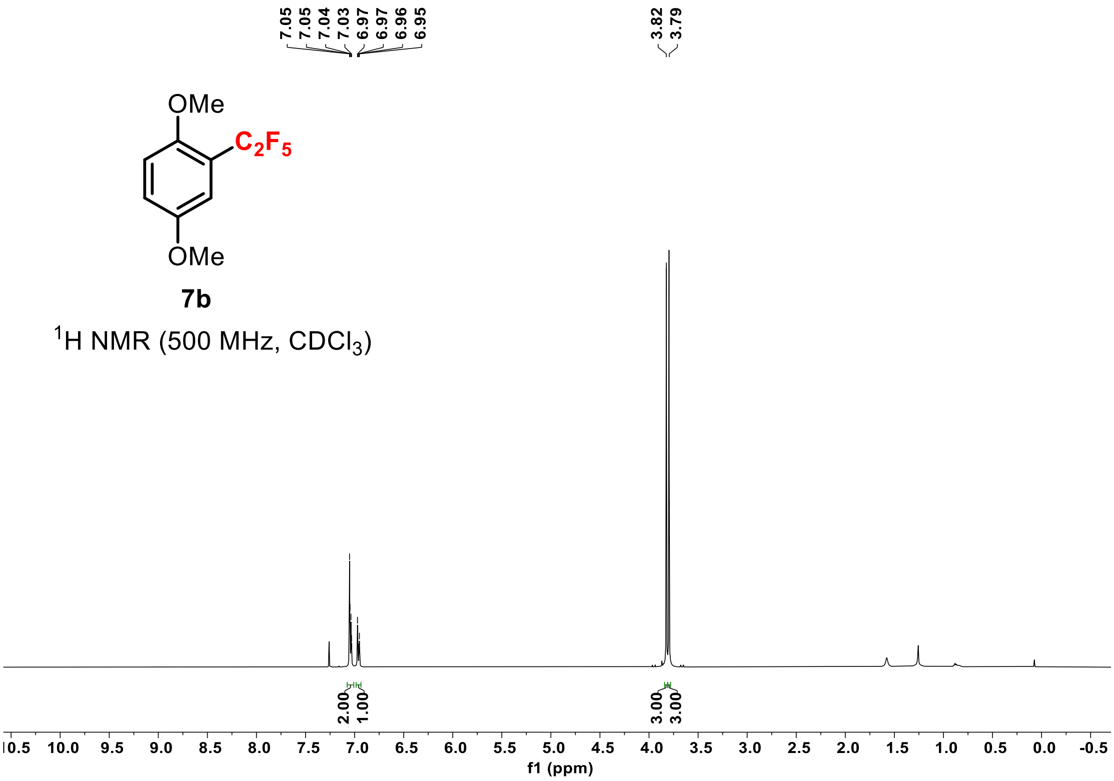

7.05
7.05
7.04
7.03
6.97
6.97
6.96
6.95
3.82
3.79
OMe
C2F5
OMe
7b
1H NMR (500 MHz, CDCl3)
2.00
1.00
3.00
3.00
f1 (ppm)

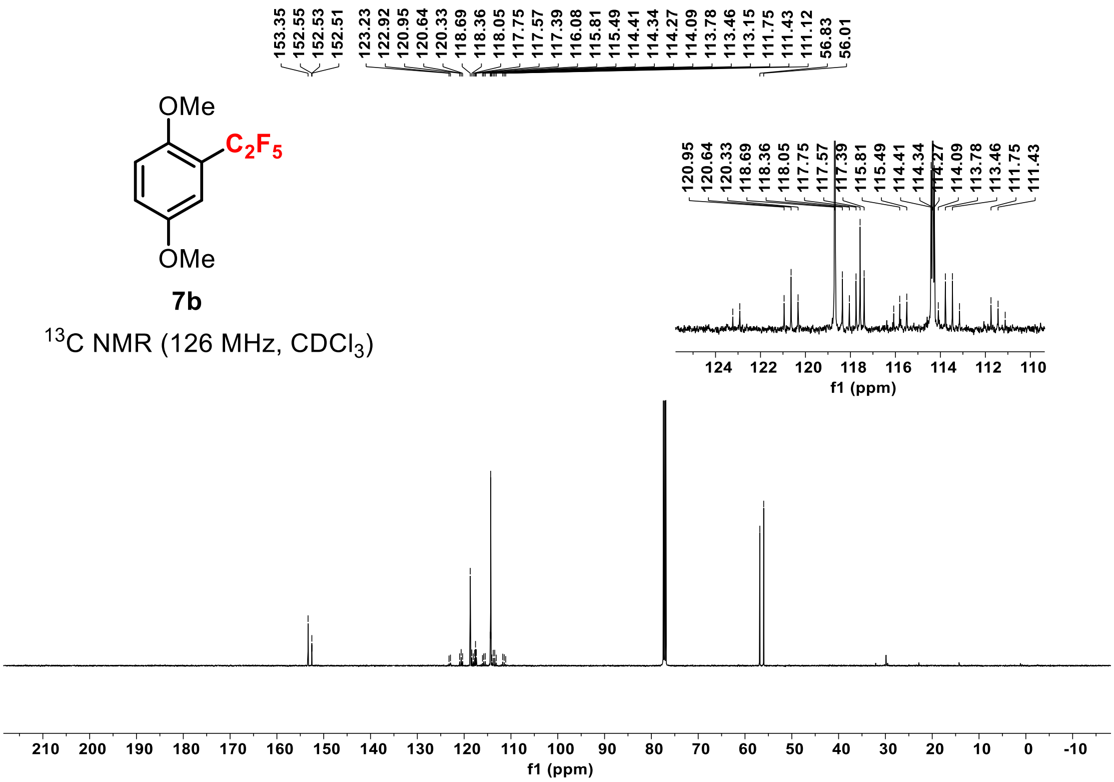

153.35
152.55
152.53
152.51
123.23
122.92
120.95
120.64
120.33
118.69
118.36
118.05
117.75
117.57
117.39
116.08
115.81
115.49
114.41
114.34
114.27
114.09
113.78
113.46
113.15
111.75
111.43
111.12
56.83
56.01
OMe
C2F5
OMe
7b
13C NMR (126 MHz, CDCl3)
f1 (ppm)

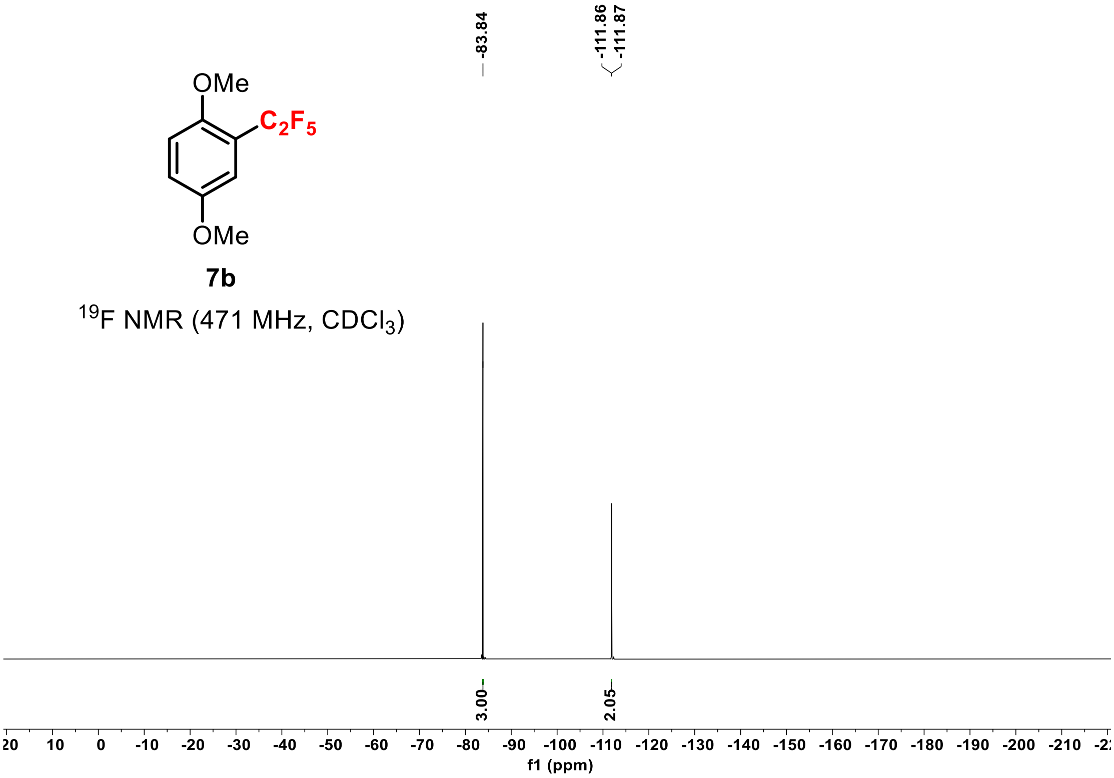

7b

^{19}F NMR (471 MHz, $CDCl_3$)

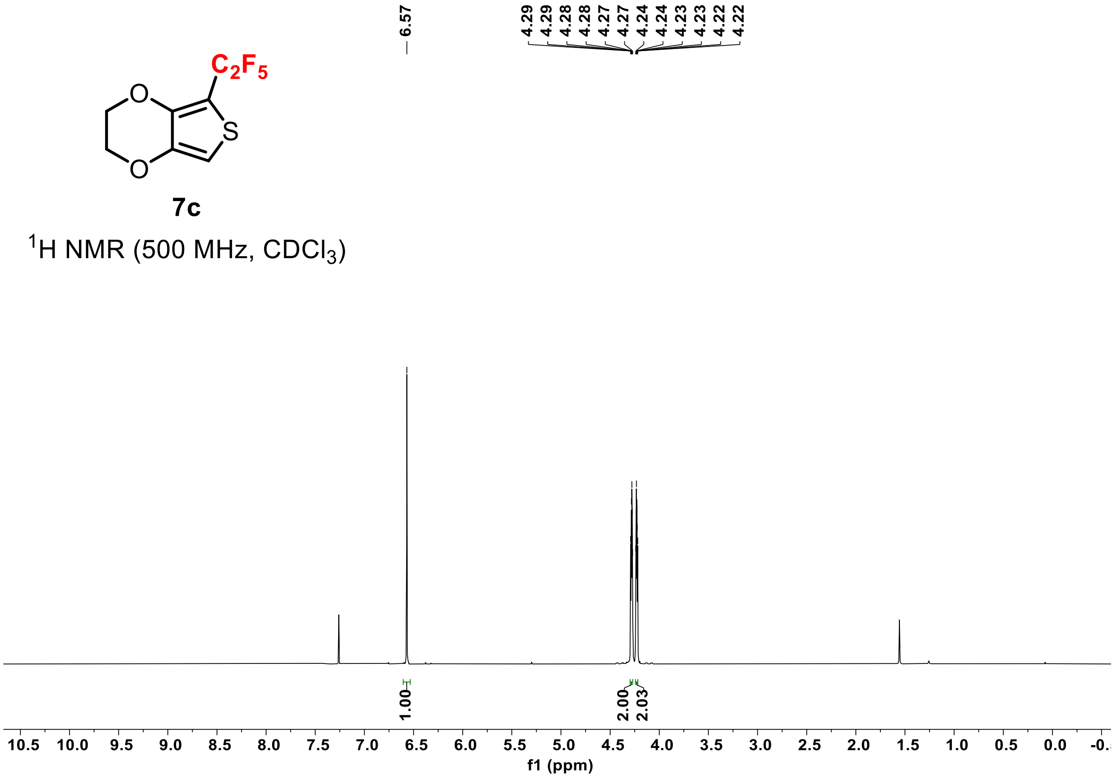

7c

^{1}H NMR (500 MHz, $CDCl_3$)

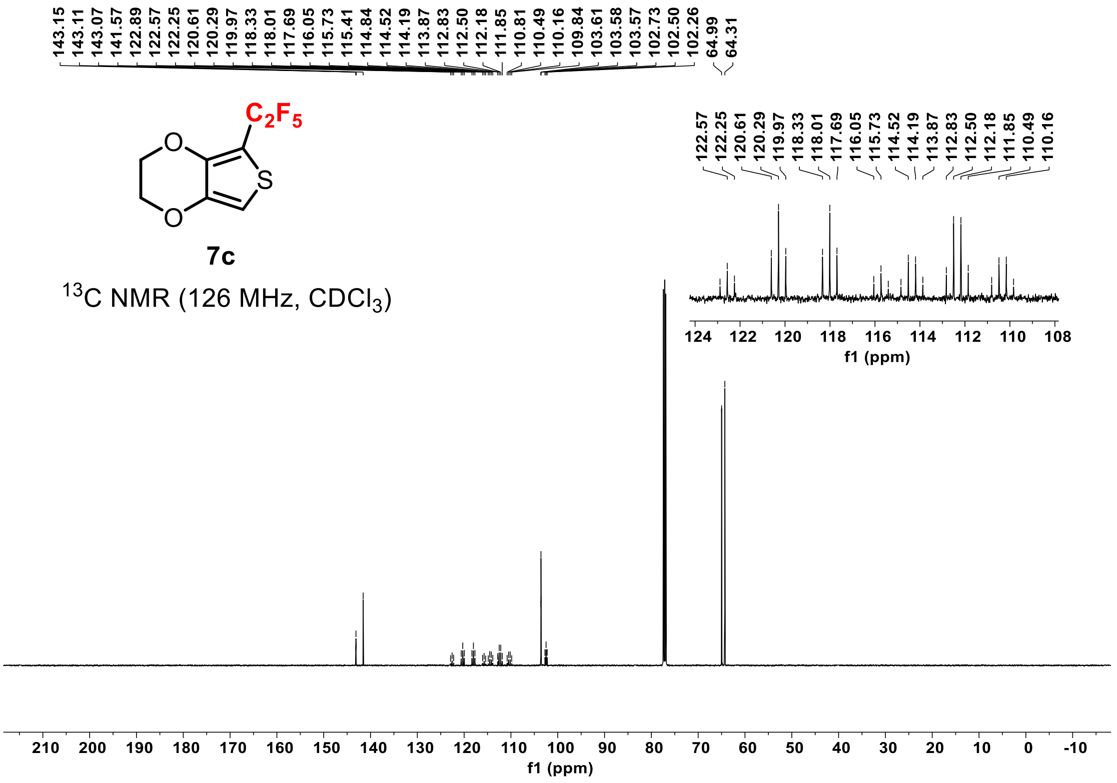
143.15
143.11
143.07
141.57
122.89
122.57
122.25
120.61
120.29
119.97
118.33
118.01
117.69
116.05
115.73
115.41
114.84
114.52
114.19
113.87
112.83
112.50
112.18
111.85
110.81
110.49
110.16
109.84
103.61
103.58
103.57
102.73
102.50
102.26
64.99
64.31
C_2F_5
O
S
O
7c
^{13}C NMR (126 MHz, $CDCl_3$)
f1 (ppm)

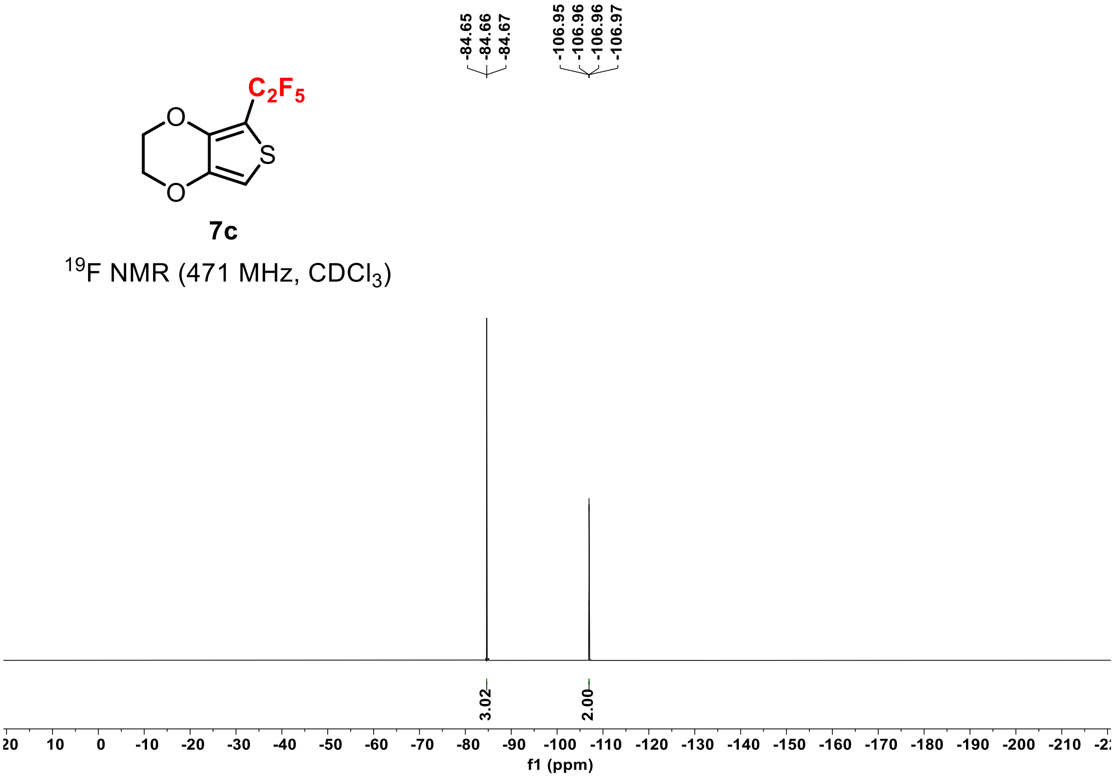
-84.65
-84.66
-84.67
-106.95
-106.96
-106.96
-106.97
C_2F_5
O
S
O
7c
^{19}F NMR (471 MHz, $CDCl_3$)
3.02
2.00
f1 (ppm)

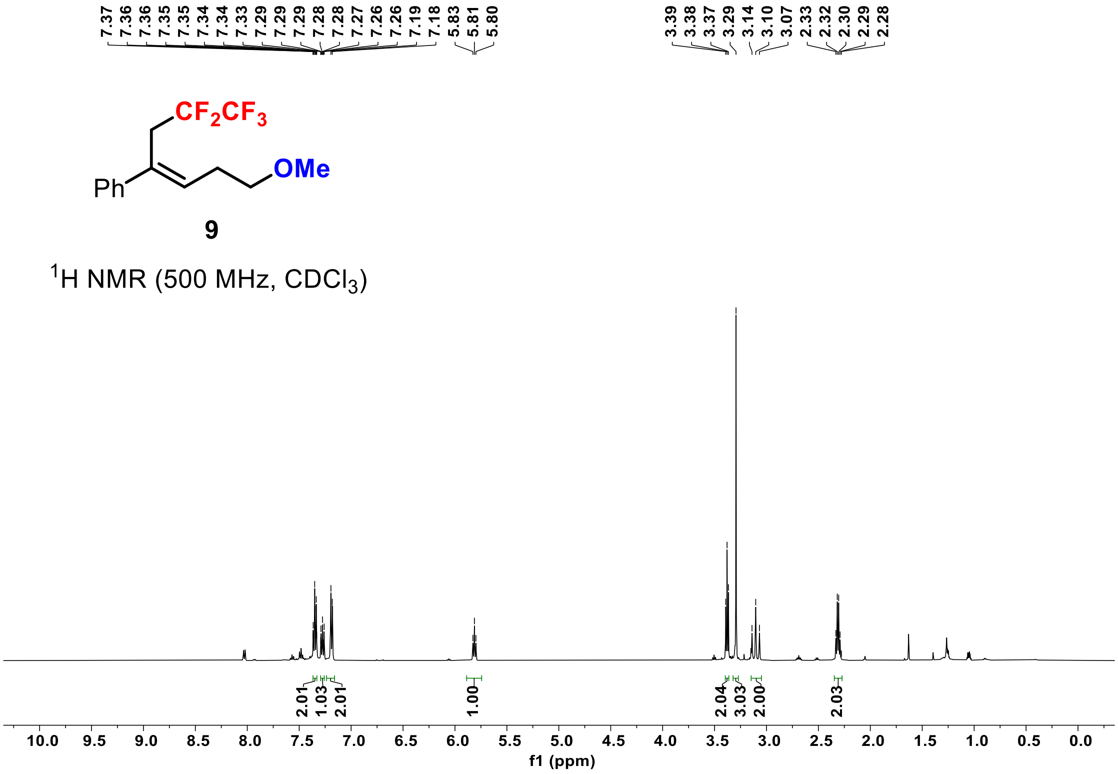

CF2CF3
Ph
OMe
9
1H NMR (500 MHz, CDCl3)
f1 (ppm)

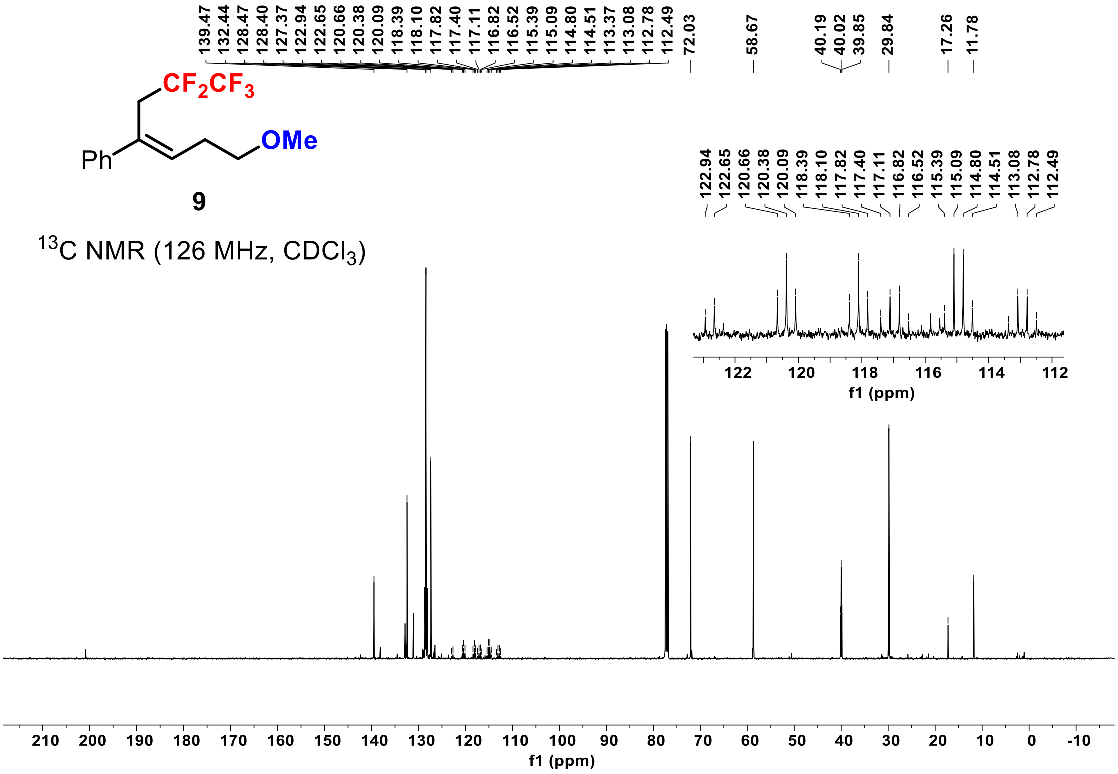

CF2CF3
Ph
OMe
9
13C NMR (126 MHz, CDCl3)
f1 (ppm)

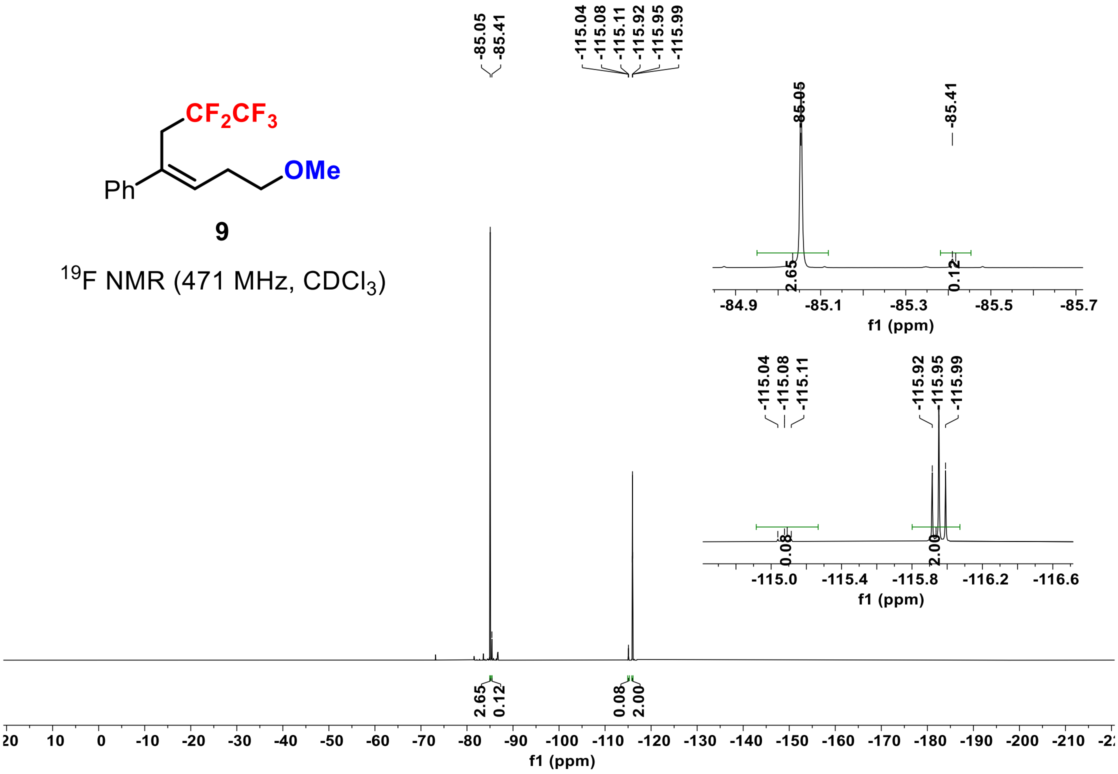

CF2CF3
Ph
OMe
9
19F NMR (471 MHz, CDCl3)
-85.05
-85.41
-115.04
-115.08
-115.11
-115.92
-115.95
-115.99
2.65
0.12
0.08
2.00
f1 (ppm)
